# Regulatory Convergence in Cell and Gene Therapy: Harmonizing Quality, CMC, and Approval Pathways Across the FDA, EMA, PMDA, and Emerging Markets

**DOI:** 10.3390/biotech15040079

**Published:** 2026-09-16

**Authors:** Nagendra Verma, Swati Arora

**Affiliations:** 1 Applied Education in the MedTech Industry, St. Cloud State University, Brooklyn Park, MN 55445, USA; 2 Department of Chemistry, University of Pittsburgh, 219 Parkman Ave, Pittsburgh, PA 15260, USA; 3 Eli Lilly and Company, Indianapolis, IN 46221, USA

**Keywords:** cell and gene therapy, regulatory harmonization, advanced-therapy medicinal products (ATMPs), Chemistry, Manufacturing, and Controls (CMC), FDA, EMA, PMDA, BRIC-M, convergence framework, potency assay

## Abstract

Cell and gene therapies (CGTs) have progressed from proof of concept to an established commercial pipeline, yet global translation remains constrained by fragmented regulatory frameworks; heterogeneous Chemistry, Manufacturing, and Controls (CMC) requirements; and divergent approval pathways. This narrative review compares CGT quality, CMC, and approval-pathway regulation across the US Food and Drug Administration (FDA), European Medicines Agency (EMA), and Japan’s Pharmaceuticals and Medical Devices Agency (PMDA), together with five emerging-market agencies: Brazil, Russia, India, China, and Mexico (BRIC-M), current to June 2026. Four convergence gaps recur across all eight jurisdictions: unstandardized potency assay validation, inconsistent post-change comparability expectations, uneven ICH guideline implementation, and divergent evidentiary thresholds for small-population trials. Convergence readiness varies sharply within the emerging-market group, from ICH Regulatory Membership and internationally benchmarked CMC guidance in China and Brazil to reference-country reliance in Mexico and observer status in Russia and India. On this basis, we propose the Global CGT Regulatory Convergence Framework (GCRC-F), a reference architecture of four independently adoptable pillars: (i) Unified CMC Standards, (ii) a Data Harmonization Layer for long-term follow-up and real-world evidence, (iii) an Adaptive Approval Layer linking accelerated designations across agencies, and (iv) a Manufacturing Standardization Layer that is built on existing regulatory precedents rather than novel instruments, with participation tiered by demonstrated regulatory-science maturity. The framework is offered as a structured proposal for discussion; it has not been evaluated by regulators or industry stakeholders, and its feasibility remains to be tested through the consultation and case-study methods identified.

## 1. Introduction

Cell and gene therapies (CGT) have advanced from proof of concept to an established commercial reality. Since the first authorizations of chimeric antigen receptor (CAR) T-cell and adeno-associated virus (AAV)-mediated products, the field has expanded to encompass ex vivo gene-modified hematopoietic stem-cell therapies and, most recently, clinically authorized genome-edited products, with landmark approvals including tisagenlecleucel, axicabtagene ciloleucel, voretigene neparvovec, and betibeglogene autotemcel. These approvals were regulatory as well as clinical milestones. Tisagenlecleucel and axicabtagene ciloleucel, autologous CD19-directed chimeric antigen receptor T-cell therapies for B-cell malignancies, established the review paradigm for individualized products in which the manufacturing process is inseparable from the product itself. Voretigene neparvovec, an adeno-associated virus vector delivering a functional RPE65 gene for inherited retinal dystrophy, was the first in vivo gene therapy approved in the United States and set a precedent for vector-based Chemistry, Manufacturing and Controls expectations and for long-term follow-up requirements. Betibeglogene autotemcel, an ex vivo lentiviral gene-modified hematopoietic stem-cell product for transfusion-dependent beta-thalassemia, established expectations for integration-site analysis and extended safety surveillance. Each therefore posed a distinct regulatory question, and the divergent ways in which agencies answered those questions form part of the fragmentation this review examines. These modalities offer durable and potentially curative benefits in conditions for which conventional pharmacology has offered only symptomatic management, and the clinical pipeline continues to expand across hematological malignancies, inherited retinal and metabolic disease, and haemoglobinopathies [1,2,3]. This rapid scientific progress has outpaced harmonized regulatory infrastructure. CGTs present a fundamentally different risk–benefit and manufacturing profile than conventional biologics, introducing several layers of complexity. First, many CGTs are personalized or autologous and are manufactured on a per-patient basis, which complicates comparability and quality-control paradigms constructed for large-scale, homogeneous manufacturing [1]. Second, target diseases are frequently rare monogenic disorders affecting small populations, constraining the feasibility of large, randomized trials and increasing reliance on preclinical evidence, first-in-human dosing informed by related therapies, and novel trial designs supporting both approval and long-term pediatric safety monitoring [2]. Third, manufacturing variability arising from differences in starting-cell material, vector production, expansion protocols, and potency assay methodology challenges product comparability across sites and across the product lifecycle, particularly for iPSC-derived and other complex cellular products for which assay robustness remains unresolved [3].

In response to these challenges, major regulators have each developed adaptive pathways: the FDA’s Breakthrough Therapy and Regenerative Medicine Advanced Therapy (RMAT) designations; the EMA’s advanced-therapy medicinal product (ATMP) framework, overseen by the Committee for Advanced Therapies (CAT) with its Conditional Marketing Authorization process; and Japan’s PMDA’s Conditional Time-Limited Approval system, permitting early commercialization paired with rigorous post-marketing surveillance [4]. While sharing the broad goal of accelerating access, comparative analysis across the US, EU, Japan, and BRIC-M over two decades shows that these frameworks differ substantially in classification criteria, evidentiary requirements, and CMC expectations, with gene-editing products in particular requiring specialized long-term follow-up and off-target safety evaluation handled inconsistently across jurisdictions [4]. International technical harmonization has narrowed some gaps: ICH Q5E establishes harmonized comparability principles for biotechnological/biological products before and after manufacturing changes [5]; ICH Q2(R2) and Q14 describe a lifecycle approach linking analytical procedure development with validation, including expanded application to complex biologics [6,7]; and ICH S12 (2023), the first ICH nonclinical guideline dedicated to gene therapies, provides harmonized recommendations for biodistribution study design informing first-in-human dosing and long-term follow-up [8]. Adoption of these guidelines remains uneven, especially in emerging markets; India’s regulatory and ethical framework for first-in-human trials, for example, has recently been the subject of capacity-building efforts aimed at international policy harmonization [9].

The result is a fragmented landscape in which a single CGT product may require jurisdiction-specific potency assays, comparability protocols, and CMC documentation to achieve authorization across regions. This imposes substantial costs on developers, particularly the smaller biotechnology companies and academic centers disproportionately represented among CGT innovators, and contributes to prolonged timelines and barriers limiting competition and patient access [1]. Industry analyses of CMC challenges across novel modalities similarly conclude that more consistent, harmonized global regulatory approaches, supported by regulatory education and mutually understood risk-based scientific tools, are urgently needed [10], and field-wide reviews emphasize regulatory harmonization, real-world evidence integration, and adaptive pathway development as among the most pressing unresolved priorities [4,11].

**Figure 1:**
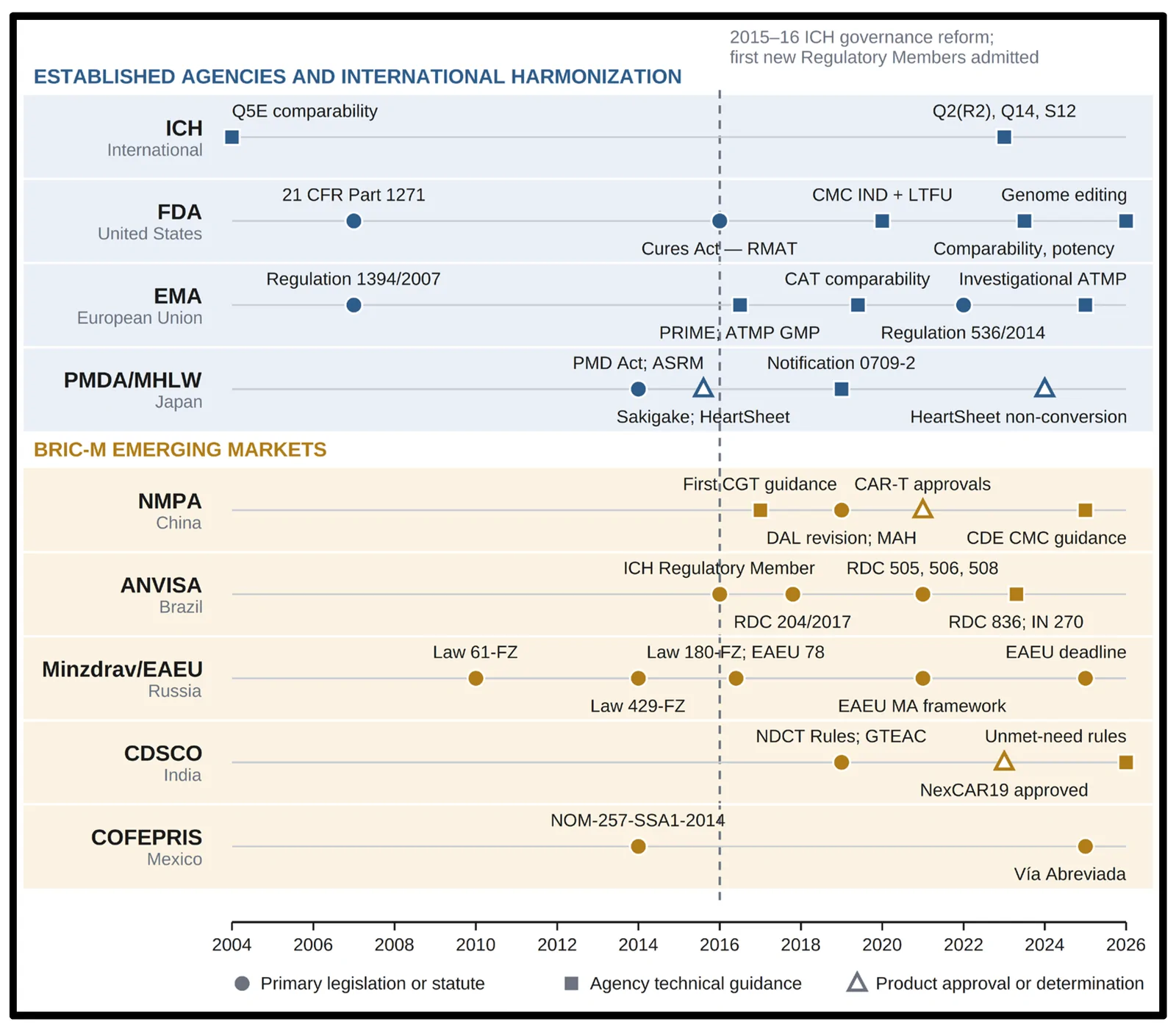
Chronological evolution of cell- and gene-therapy regulatory frameworks across eight regulatory authorities and the International Council for Harmonization, 2004–2026. Marker shape denotes instrument type: primary legislation (filled circle), agency technical guidance (filled square), and product approval or approval-related determination (open triangle). The vertical reference line marks the 2015–2016 reform of ICH governance and the admission of the first cohort of new Regulatory Members. Selected landmark instruments are shown; full document references are given in Section 2 and Section 3. Abbreviations: ASRM, Act on the Safety of Regenerative Medicine; BTD, Breakthrough Therapy Designation; CDE, Center for Drug Evaluation; CTL, conditional and time-limited approval; DAL, Drug Administration Law; EAEU, Eurasian Economic Union; GTEAC, Gene Therapy Advisory and Evaluation Committee; LTFU, long-term follow-up; MAH, Marketing Authorization Holder; NDCT, New Drugs and Clinical Trials; RMAT, Regenerative Medicine Advanced Therapy. The 2004 start point marks the adoption of ICH Q5E, the first internationally harmonized instrument directly applicable to cell- and gene-therapy products; earlier national provisions, including the US Public Health Service Act framework and 21 CFR Part 1271, predate this period and are discussed in Section 2.1. The figure traces the emergence of product-class-specific regulatory architecture rather than the full history of biologics regulation and terminates at the review’s data cutoff of 30 June 2026.

Given this context, this narrative review compares CGT quality, CMC, and approval-pathway regulation across the FDA, EMA, PMDA, and BRIC-M, with particular attention to potency assay development and bridging, platform technology designations, and long-term follow-up requirements. Building on this analysis, it proposes a Global CGT Regulatory Convergence Framework (GCRC-F) to identify proposed harmonization opportunities, reduce redundant regulatory burden, accelerate global access to transformative CGT products, and inform both regulatory policy and sponsor strategy (Figure 1).

### Scope, Sources and Search Strategy

This article is a narrative review. It synthesizes peer-reviewed literature and primary regulatory instruments governing cell- and gene-therapy products across eight jurisdictions, and it does not claim the exhaustive coverage or prespecified synthesis of a systematic review. Peer-reviewed sources were identified through PubMed, Embase, Scopus and Web of Science, combining terms for cell-therapy, gene-therapy and advanced-therapy medicinal products with terms for regulation, harmonization, convergence, reliance, comparability, potency and good manufacturing practice, for the period January 2005 to June 2026. Reference lists of retrieved articles were hand-searched for additional sources. Primary regulatory instruments were retrieved directly from the official portals of the FDA, EMA, PMDA, NMPA, ANVISA, CDSCO, COFEPRIS, Eurasian Economic Commission, ICH, ICMRA and WHO. Sources were included where they described the legal basis, technical requirements, or operational practice of CGT regulation in one or more of the jurisdictions examined. Sources were excluded where they addressed conventional biologics without CGT-specific content, where they were superseded by a later instrument, or where the primary instrument was available and a secondary commentary added no independent analysis. Where an instrument existed in both draft and final form, the version operative as of 30 June 2026 was used, and its status is stated in the text and tables. Selection was purposive rather than exhaustive: the objective was comprehensive coverage of the regulatory architecture in each jurisdiction rather than complete capture of the primary literature. No formal risk-of-bias appraisal was undertaken, as no validated instrument exists for appraising regulatory instruments, which are legal documents rather than intervention studies. The reporting approach follows the quality criteria for narrative reviews described by Baethge et al. [12] and Sukhera [13]; PRISMA reporting was not adopted because its authors state that it is designed primarily for systematic reviews of studies evaluating the effects of health interventions [14]. Statements that a given framework, guidance or threshold does not exist reflect the absence of a publicly available instrument identified through these sources as of 30 June 2026 and should be read accordingly.

## 2. Regulatory Frameworks of the Established Agencies: FDA, EMA, and PMDA

### 2.1. United States: CBER Oversight, RMAT Designation, and Platform CMC Leveraging

In the United States, cell- and gene-therapy (CGT) products are regulated by the Center for Biologics Evaluation and Research (CBER) within the FDA, under the authority of Section 351 of the Public Health Service Act and, for certain minimally manipulated tissue products, under Section 361 and 21 CFR Part 1271 [15]. Products that involve more than minimal manipulation, are intended for non-homologous use, or have a systemic effect dependent on living cell metabolism are regulated as biological products requiring an Investigational New Drug (IND) application and, ultimately, a Biologics License Application (BLA) [16].

To address the distinctive manufacturing and developmental challenges posed by CGTs, CBER has issued a series of product-class-specific guidance documents. The foundational Guidance for FDA Reviewers and Sponsors: Content and Review of Chemistry, Manufacturing, and Control (CMC) Information for Human Gene Therapy Investigational New Drug Applications (INDs), first issued in 2008 and finalized in its current form in January 2020, provides recommendations on the CMC information sponsors must submit to assure the identity, quality, purity, and potency of investigational gene-therapy products, explicitly acknowledging the manufacturing variability challenges introduced in Section 1 of this review as a central regulatory concern [17,18]. This was complemented in July 2023 by draft guidance on Manufacturing Changes and Comparability for Human Cellular and Gene Therapy Products, which articulates a lifecycle-based approach to managing post-approval manufacturing changes and the comparability studies needed to demonstrate that such changes do not adversely affect product quality, safety, or efficacy, a principle directly aligned with the comparability framework established at the international level by ICH Q5E (see Section 1) [19].

The principal expedited approval mechanism for CGTs is the Regenerative Medicine Advanced Therapy (RMAT) designation, established under Section 3033 of the 21st Century Cures Act and codified in Section 506(g) of the Federal Food, Drug, and Cosmetic Act. A product is eligible for RMAT designation if it qualifies as a regenerative-medicine therapy, defined to include cell therapies, therapeutic tissue engineering products, and human cell and tissue products; is intended to treat a serious or life-threatening disease; and is supported by preliminary clinical evidence of the potential to address an unmet medical need [16]. Comparative regulatory analyses have noted that RMAT designation confers all the benefits of Breakthrough Therapy designation, including intensive FDA guidance on an efficient development program and eligibility for accelerated approval based on a surrogate or intermediate endpoint reasonably likely to predict clinical benefit, with the additional possibility of fulfilling post-approval requirements through real-world evidence, including data collected through registries and other observational sources [20]. These two evidentiary concepts operate at different points in development and are worth distinguishing. “Preliminary clinical evidence” is the threshold for granting RMAT designation, and the FDA assesses it case by case against the rigor of data collection, the consistency and persuasiveness of the outcomes, the number of patients and sites contributing, and the severity or rarity of the condition. Critically, and unlike Breakthrough Therapy designation, RMAT does not require the sponsor to show that the product offers substantial improvement over available therapy [16]. A surrogate or intermediate clinical endpoint, by contrast, is the basis on which the product may later receive accelerated approval under Section 506(c) of the FD&C Act. The FDA defines a surrogate endpoint as a marker, such as a laboratory measurement or radiographic image, thought to predict clinical benefit but not itself a measure of it, and an intermediate clinical endpoint as a measurement of a therapeutic effect that can be assessed earlier than an effect on irreversible morbidity or mortality and is reasonably likely to predict it [16]. In practice, CGT-accelerated approvals to date have relied predominantly on surrogate endpoints: brexucabtagene autoleucel (2020, relapsed or refractory mantle cell lymphoma) and axicabtagene ciloleucel (2021, relapsed or refractory follicular lymphoma) were both approved on overall response rate and duration of response [21,22]. The FDA has itself noted limited experience with accelerated approvals based on intermediate clinical endpoints. This RWE-based approach to post-marketing confirmation is particularly significant for products targeting the small patient populations described in Section 1, where conventional confirmatory randomized trials may not be feasible.

Most recently, CBER’s evolving approach to CMC flexibility has extended to genome-editing products specifically. A 2026 draft guidance permits sponsors of somatic genome-editing gene therapies to leverage publicly available information and platform-level CMC, nonclinical, and clinical data, provided a product-specific scientific rationale justifies the applicability of the leveraged data, with early regulatory alignment encouraged through CBER’s Initial Targeted Engagement for Regulatory Advice (INTERACT) and pre-IND meeting programs [23,24]. This platform-based approach applies, in the CGT context, a mechanism that already exists in US law. The Platform Technology Designation Program is established under Section 506K of the Federal Food, Drug, and Cosmetic Act, added by Section 2503 of the PREVENT Pandemics Act within Public Law 117-328 (2022), and was operationalized through FDA draft guidance issued in May 2024 [25]. The statutory definition expressly encompasses a nucleic acid sequence, molecular structure, mechanism of action, delivery method, or vector, and therefore covers the vector platforms central to gene-therapy manufacture. Designation permits a sponsor to leverage data previously submitted on the designated platform in support of subsequent applications. Our claim here is not that platform technology requires further investigation, but that the United States has created a legal mechanism for platform leveraging that most other jurisdictions examined in this review have not, and Section 4 compares the jurisdictions on precisely that point that is examined comparatively across jurisdictions in Section 4 of this review. It reflects the FDA’s broader commitment, reiterated through CBER’s 2026 “flexible approach” communications, to calibrate CMC and comparability expectations to the magnitude of risk posed by a given manufacturing change rather than applying uniform requirements across all CGT product classes [26].

### 2.2. European Union: The ATMP Regulation, the CAT, and the PRIME Scheme

The European system distributes responsibility for advanced-therapy medicinal products across four bodies, and the division of labor is worth stating before the details. The Committee for Advanced Therapies (CAT) is the specialist body: it performs the scientific assessment of an ATMP application and prepares a draft opinion. The Committee for Medicinal Products for Human Use (CHMP) then adopts the formal opinion, taking the CAT draft as its basis. The European Medicines Agency (EMA) hosts both committees and transmits the adopted opinion. The European Commission takes the final legally binding decision granting or refusing the marketing authorization, which is then valid across all Member States. In short, the CAT assesses, the CHMP opines, the EMA coordinates, and the Commission authorizes. All ATMPs must follow this centralized route; national authorization is not available for them. The European Union established a dedicated regulatory category for CGTs, gene-therapy medicinal products, somatic-cell-therapy medicinal products, and tissue-engineered products, collectively termed advanced-therapy medicinal products (ATMPs), under Regulation (EC) No. 1394/2007. The Regulation mandates that all ATMPs be authorized through the centralized procedure coordinated by the EMA. Clinical trial authorization is governed separately by Regulation (EU) No. 536/2014, applicable from 31 January 2022, with the transition period completed on 31 January 2025, under which applications are submitted through the Clinical Trials Information System (CTIS) and subjected to coordinated assessment: Part I is assessed jointly by the Member States concerned under a Reporting Member State, while Part II remains the responsibility of each National Competent Authority [27]. Scientific evaluation of ATMP marketing authorization applications is performed by the Committee for Advanced Therapies (CAT), a multidisciplinary committee established specifically for this purpose, which generates a draft opinion that is subsequently submitted to the Committee for Medicinal Products for Human Use (CHMP) for a final recommendation [27].

For every ATMP application, sponsors must submit comprehensive data and information relating to manufacturing processes and quality control of both the active substance and the final product, alongside non-clinical and clinical safety and efficacy data, with technical requirements further elaborated through a suite of EMA/CAT product-class guidelines [27]. Recognizing the inherent heterogeneity of ATMPs, in particular the autologous, patient-specific manufacturing processes that characterize many cell-based products, Regulation 1394/2007 introduced a risk-based approach permitting adapted regulatory requirements; this flexibility subsequently informed the development of ATMP-specific good manufacturing practice (GMP) guidelines tailored to the small-batch, decentralized manufacturing settings common to autologous products [27]. An earlier account of this framework similarly emphasized that the diversity of ATMPs necessitates a tailored, risk-based regulatory approach, and documented that a 2013 public consultation on the ATMP Regulation, with results published in 2014, proposed several improvements to the regulatory and procedural framework that remain relevant to ongoing convergence discussions [28]. The combination of this risk-based regulatory architecture and enhanced regulatory support mechanisms has contributed to a growing number of successful ATMP marketing authorizations, with the majority of licensed products to date being gene-therapy medicinal products [27].

The EU’s principal expedited regulatory tool for ATMPs is the PRIME (PRIority MEdicines) scheme, introduced by the EMA to provide enhanced support for the development of medicines that target unmet medical needs. As discussed further in Section 2.3 below, PRIME was developed contemporaneously with, and shares conceptual features with, the FDA’s Breakthrough Therapy designation and Japan’s Sakigake designation, though a defining feature distinguishing PRIME is its explicit focus on actively supporting ATMP development by academic institutions and small or medium-sized sponsors, a population of developers that, as noted in Section 1, is disproportionately represented among CGT innovators [20]. At the quality and CMC level, the comparability principles articulated in ICH Q5E and the analytical procedure lifecycle approach of ICH Q2(R2) and Q14, both adopted by the EMA as Step 5 guidelines (see Section 1), apply directly to ATMP marketing authorization dossiers, providing one of the clearest existing points of technical convergence between the EU framework and those of the FDA and PMDA.

### 2.3. Japan: Conditional and Time-Limited Approval, Sakigake, and the HeartSheet Precedent

Japan’s regulatory framework for CGTs is built upon two complementary pieces of legislation that came into effect in November 2014: the Act on the Safety of Regenerative Medicine (ASRM) and the Act on Securing Quality, Efficacy, and Safety of Products, including Pharmaceuticals and Medical Devices (PMD Act). The ASRM establishes the responsibilities of medical institutions in ensuring the safety and transparency of regenerative medicine technologies, while the PMD Act provides sponsors with the option of a conditional and time-limited approval scheme specifically for CGT products [29].

The conditional and time-limited approval pathway is a distinctive feature of the Japanese system, permitting regenerative medical products to reach the market based on demonstrated safety and a reasonable expectation of efficacy, subject to a defined post-marketing data collection period after which the PMDA conducts a formal re-review to determine whether full approval is warranted. The Ministry of Health, Labor and Welfare (MHLW) established the first dedicated guidance on this scheme, the Guidance for Conditional and Time-Limited Approval for Regenerative Medical Products and the Development of Subsequent Efficacy Evaluation Plan, in March 2024, which provides specific examples of products falling within the scope of the scheme and clarifies the considerations applied during the post-marketing approval condition assessment [30]. The efficacy standard applied under this pathway warrants clarification, because it is qualitative rather than quantitative. Articles 23–26 of the PMD Act require that the product be presumed to have the efficacy, effect or performance for the application; no effect size, statistical criterion or numerical benchmark defines that presumption. The March 2024 guidance confirms that prior approvals were determined case by case according to the content of the application data and characterizes qualifying evidence only as information of a certain degree of clinical significance [30]. The assessment is accordingly product-specific and reached through face-to-face consultation with PMDA rather than against a general threshold. The guidance does supply reference sample-size tables based on a two-sided significance level of 10 percent and 80 percent power, but these are expressly planning references and not approval criteria. This absence of a defined evidentiary threshold has attracted sustained critique, and the HeartSheet case discussed below illustrates both the flexibility and the risk it creates [31]. The practical rigor of this pathway was demonstrated by the case of HeartSheet, an autologous human skeletal myoblast-derived cell sheet product and the first product approved under the conditional and time-limited scheme, following PMDA deliberation on 2 September 2015 and conditional approval in September 2015. On 19 July 2024, following PMDA re-review of the post-marketing data package, the MHLW Subcommittee on Regenerative Medicine Products and Biotechnology confirmed that the efficacy criteria established at conditional approval had not been met and that conversion to regular approval was not appropriate; the sponsor discontinued sales the following day. This remains the first and only instance in which post-marketing evidence has terminated a conditionally approved regenerative medical product in Japan, and it establishes that the conditional pathway carries genuine post-marketing accountability rather than functioning as a de facto permanent authorization [30].

Japan’s principal expedited designation mechanism, analogous to the FDA’s RMAT and EMA’s PRIME, is the Sakigake designation, administered by the PMDA. The Sakigake designation shares many features with the breakthrough therapy and RMAT designations originally developed by the FDA, but its defining original feature is the requirement that the product be first developed in Japan, reflecting a national policy objective of positioning Japan as a leading site for early-stage regenerative medicine innovation [20]. The same comparative analysis catalogued all drugs and regenerative medical products that have received conditional approval or Sakigake designation in Japan, providing an empirical basis for cross-jurisdictional comparison of expedited pathway utilization that is revisited in the comparative analysis presented in Section 2.4 [20].

### 2.4. Comparative Synthesis: Evidentiary Philosophy and the Limits of CMC Convergence

Taken together, the FDA, EMA, and PMDA frameworks summarized in Table 1 reveal a recurring pattern: each agency has independently developed an expedited pathway—RMAT, PRIME, and Sakigake, respectively—rooted in a shared premise that conventional approval timelines are poorly suited to CGTs targeting serious unmet needs in small patient populations, yet each diverges in eligibility criteria and procedural emphasis [20]. Beneath this structural parallel lies a philosophical spectrum in acceptable evidentiary risk. The FDA requires substantial evidence of effectiveness but permits RMAT-designated products to rely on surrogate endpoints, with post-approval confirmation potentially satisfied through real-world evidence [16]. The EMA’s Conditional Marketing Authorization treats conditionality as an explicitly codified approval state rather than an exception, subject to obligatory confirmatory studies [27,28]. Japan’s PMD Act goes furthest in permitting early market access on limited efficacy evidence while enforcing genuine post-marketing accountability, as illustrated by the 2024 HeartSheet non-renewal [30]. This substantial-evidence-to-time-limited-access spectrum, detailed in full in Table 1, is the essential baseline against which the wider BRIC-M spectrum examined in Section 3 must be assessed, where, as will be shown, emerging-market frameworks span an even wider range, from China’s multi-pathway system to Mexico’s near-total reference-country dependence.

At the CMC and quality level, all three jurisdictions have converged in direction, if not yet in operational detail, toward risk-based, lifecycle-oriented comparability frameworks anchored to ICH Q5E, Q2(R2)/Q14, and S12 [5,6,7,8,17,27,29]. Whether this directional convergence has translated into practical harmonization, particularly for potency assay acceptance criteria, platform technology recognition, and long-term follow-up design, remains uneven and is examined in Section 4.

## 3. CGT Regulatory Landscape Across the BRIC-M Emerging Markets (Brazil, Russia, India, China, Mexico)

The FDA-EMA-PMDA spectrum established in Section 2.4, from substantial pre-approval evidence, through benefit–risk conditionality, to time-limited early access, provides the baseline against which emerging-market regulatory posture must be assessed. Brazil, Russia, India, China, and Mexico (BRIC-M) form an analytically coherent unit not merely as major emerging economies, but also because each hosts, or is developing, a domestic CGT sector of sufficient scale to require a dedicated regulatory response while confronting structural limitations in regulatory maturity, enforcement capacity, and harmonization infrastructure relative to Section 2’s established agencies. A systematic curation of CGT regulations across countries has documented that frameworks lack standardization in terminology, format, and regulatory category, creating challenges for multinational supply chains and simultaneous multi-market development [50].

The five jurisdictions examined in this section were selected as a purposive sample rather than as a regulatory grouping. Brazil, Russia, India, China and Mexico correspond to the “pharmerging” markets first identified by IMS Health, now IQVIA, a classification based on pharmaceutical market size and growth trajectory rather than on regulatory maturity [51]. They were chosen because they represent the largest middle-income pharmaceutical markets outside the ICH founding regions, and because together they illustrate the full range of convergence challenges such markets face. They are not a homogeneous group, and this review does not treat them as one. According to the WHO Global Benchmarking Tool assessment, India and China hold Maturity Level 3 for vaccines only, while Brazil, Russia, and Mexico have not currently published ML3 or ML4 designation [52]. Two are ICH Regulatory Members, one is a Regulatory Member with limited implementation, and two participate only as observers. The heterogeneity within the group is itself analytically informative, and Section 3.6 examines it directly.

We emphasize that this is a sampling frame and not a claim of regulatory similarity. The pharmerging classification tiers these markets by pharmaceutical spending, placing China, Brazil and India in the first tier and Russia and Mexico in the second, and it does not imply regulatory convergence capacity. On regulatory indicators, the five diverge sharply, spanning full ICH Regulatory Membership to Observer status, PIC/S participation to non-participation, and dedicated CGT legislation to none, as Supplementary Table S1 sets out. It is precisely this heterogeneity within a group of comparably scaled markets that makes the comparison analytically useful, and no part of the framework proposed in Section 8 assumes the five would converge at the same rate or through the same mechanisms.

### 3.1. China: The NMPA Regulatory Framework, Expedited Pathways, and Convergence Trajectory

#### 3.1.1. Legislative and Institutional Framework

China represents the most advanced BRIC-M jurisdiction in CGT regulation, driven by an extraordinary volume of clinical trial activity, deliberate national policy investment in a globally competitive biopharmaceutical industry, and the largest CGT pipeline outside the United States and Europe. The primary regulatory authority is the National Medical Products Administration (NMPA), established in 2018 following a sweeping institutional restructuring that replaced the China Food and Drug Administration (CFDA) and integrated oversight under the State Administration for Market Regulation (SAMR) [53]. The NMPA operates under the revised Drug Administration Law (DAL) of 2019, which replaced the previous product-specific registration mechanism with a risk-based classification system more closely aligned with ICH principles and introduced the Marketing Authorization Holder (MAH) framework, enabling decoupling of manufacturing from approval authorization, a structural shift directly relevant to the scale-up challenges of autologous and complex allogeneic CGT products [53,54].

CGTs are regulated as a distinct sub-category of biological products, with primary technical evaluation conducted by the NMPA’s Center for Drug Evaluation (CDE) and laboratory testing and reference standard development supported by the National Institutes for Food and Drug Control (NIFDC). The NMPA’s own CDE investigators documented that China issued its first dedicated guidance for cellular-therapy products in 2017 and has since produced a series of technical guidance documents covering CAR-T manufacturing requirements, viral vector characterization, in vivo gene-therapy CMC standards, and long-term follow-up design [53]. In 2022, CDE issued dedicated CMC guidance for both in vivo gene-therapy products and gene manipulation systems used in ex vivo gene-therapy manufacturing, and in July 2025, it released draft guidance on pharmaceutical change research and evaluation for cell-therapy products that introduces a three-tier risk classification system for manufacturing changes [54]. This 2025 draft guidance is particularly significant for the convergence objectives of this review, as it explicitly references ICH Q5E, the FDA’s 2023 CGT comparability draft guidance, and the EMA ATMP Q&A framework as international benchmarks, representing the clearest signal to date that the NMPA is deliberately aligning its CMC lifecycle expectations with those of the established agencies described in Section 2 [5,54,55].

#### 3.1.2. Expedited Approval Pathways

China’s toolkit includes Breakthrough Therapy Designation (BTD, formalized in 2020) for innovative drugs with meaningful clinical advantage, conferring priority communication, rolling review, and a dedicated review team [53]. By the end of 2022, eight CAR-T and two AAV gene-therapy products had received BTD, and six CAR-T products had received Priority Review, while axicabtagene ciloleucel (Axi-cel) and relmacabtagene autoleucel (Relma-cel) were conditionally approved in 2021 [50]. China’s approval trajectory shows year-over-year growth and flexible use of surrogate endpoints and single-arm trials, though drug lags relative to the US and EU remain the longest among the four regions analyzed [56]. A pooled analysis of Chinese conditional approvals granted between 2020 and 2023 found that no conditionally approved product had been withdrawn from the market by the NMPA, and concluded that the approval procedures, clinical evidence evaluation systems, pharmacovigilance arrangements and confirmatory trial requirements within the conditional approval framework require further enhancement [57]. Notably, on timeliness of confirmatory trial completion, the comparison does not run in one direction: an analysis of anticancer conditional and accelerated approvals supported by single-arm trials found that 26.9 percent of US approvals under verification had exceeded their confirmatory trial deadline, against 5.1 percent in China [58]. The standard NMPA marketing-authorization review clock is 200 working days, reduced to 130 under Priority Review, and 70 for rare-disease drugs with urgent clinical need already marketed overseas [53]. A distinctive dual-track architecture allows clinical development via investigator-initiated trials (IITs, regulated by the NHC) alongside formal NMPA IND pathways [54]. The complexity created by this architecture lies at the transition between the two tracks rather than in any absence of oversight. Investigator-initiated trials are supervised by the NHC as medical technology within accredited institutions, while registrational development proceeds through the NMPA as a biologic, and as of August 2024, investigator-initiated trials accounted for approximately 90 percent of registered Chinese cell-therapy trials [59]. Data generated on the investigator-initiated track frequently cannot support an NMPA marketing application without bridging, because the investigational product may not have been manufactured under commercial GMP conditions and the trial may not have been conducted to ICH GCP standards. Published analysis of the dual-track system concludes that it facilitates translational research but raises concerns regarding participant risk and the reliability of research data [54]. This gap is being addressed: the State Council’s Regulations on the Administration of Clinical Study of New Biomedical Technologies (Order No. 818), released in October 2025 and in force from 1 May 2026, establish a national framework for such studies and extend the right to initiate them beyond medical institutions. Foreign multi-regional clinical trial (MRCT) data acceptance that provisions exist, but implementation in pivotal CGT trials has been limited, with drug lags persisting partly due to inconsistent foreign-data acceptance and continued domestic bridging-study requirements, compounded by an underdeveloped orphan designation framework [56].

#### 3.1.3. Clinical Trial Environment and Manufacturing Infrastructure

China has registered the largest national share of CAR-T clinical trials worldwide. However, an analysis of 1580 CAR-T trials registered on ClinicalTrials.gov discovered proportionally few studies from China. The direction of this undercount is systematic rather than incidental. Registration on ClinicalTrials.gov is statutorily required for applicable US trials, so US activity is captured close to completely, whereas many Chinese sponsors register in the Chinese Clinical Trial Registry (ChiCTR), the national registry, which ClinicalTrials.gov does not index. An assessment covering the period to 30 June 2020 found 357 Chinese CAR-T trials registered on ClinicalTrials.gov alongside as many as 150 further trials registered only on ChiCTR [60]. Comparisons drawn from a single registry should therefore be read as a lower bound on Chinese activity, and we state registry scope and data cut-off wherever counts are reported. Through April 2024, China led in cumulative registrations, although Chinese registrations declined between 2022 and 2023, while United States registrations rose steadily; because that analysis excludes the Chinese Clinical Trial Registry, it is likely to understate Chinese activity. Trial activity is concentrated predominantly in hematological malignancies and, increasingly, in solid tumors [61,62]. The clinical trial environment is supported by a growing network of GCP-certified hospital research centers, large patient pools for rare-disease trials, and a relatively efficient institutional review process [53,54]. Manufacturing infrastructure has developed substantially, with Chinese-owned CDMOs such as WuXi Advanced Therapies offering viral vector production and cell-therapy manufacturing at scale. CDE investigators confirmed that GMP compliance across the sector has improved significantly, though independent assessments have identified persistent gaps in process validation documentation, environmental monitoring, and quality management system maturity relative to FDA and EMA expectations, representing the main manufacturing-level gap for sponsors seeking simultaneous regulatory submissions in China and Western markets [53]. Post-marketing surveillance for conditionally approved products remains a recognized developmental gap. A systematic comparative study of CGT post-marketing surveillance frameworks across the EU, the US, Japan, South Korea, and China concluded that China’s PMS regulations remain general in nature and lack the detailed product-specific guidance characterizing the EU, US, and Japanese frameworks: notably, 88% of EU-approved CGTPs are under additional monitoring, the FDA has released 34 CGT-specific guidelines spanning the product lifecycle, 22 percent of approved US CGT products carry a REMS, and Japan applies all-case surveillance to 79% of approved products, while China’s framework is currently composed of general statements without equivalent product-specific requirements [41]. This PMS gap is a primary area in which China’s otherwise sophisticated regulatory architecture most clearly lags behind the established agencies and represents a priority convergence challenge.

#### 3.1.4. Convergence Readiness and Harmonization Trajectory

China is the most advanced BRIC-M jurisdiction in convergence readiness with the established regulatory agencies. The NMPA joined the ICH as a full Regulatory Member in 2017 and was subsequently elected to the ICH Management Committee in 2018, 2021, and 2024, with the 2024 re-election explicitly recognized as demonstrating that China’s new drug R&D and registration technical standards are aligned with international rules [63]. As of 2021, China had transformed and implemented 46 ICH guidelines through formal announcements, and its CTD dossier format is aligned with ICH M4, reducing the reformatting burden for sponsors filing simultaneously in China and Western markets [53,63]. The NMPA participates in the International Pharmaceutical Regulators Program (IPRP) and has engaged in bilateral scientific exchange programs with both the FDA and the EMA, including a January 2025 FDA-NMPA bilateral meeting [63]. Significant challenges remain at the technical level. NMPA guidance for CGTs is less harmonized with EMA and FDA standards in potency assay validation specifics, comparability protocols for post-approval manufacturing changes, and LTFU design requirements, deficiencies that are structurally documented by the post-marketing surveillance gap identified by Cai et al. and the drug-lag and confirmatory-trial oversight gaps identified by Huang et al. [41,53,56]. Geopolitical factors affecting technology transfer and data-sharing have introduced uncertainty into China’s convergence trajectory, and the absence of a fully operationalized orphan designation framework for CGTs limits incentive alignment with the FDA and EMA systems [56]. Nonetheless, the combination of the NMPA’s ICH membership and management committee participation, its explicit alignment of the 2025 CMC draft guidance with international standards, and the scale and maturity of its CGT clinical and manufacturing infrastructure position China as the most credible and consequential BRIC-M partner for a structured regulatory convergence initiative.

### 3.2. Brazil: The ANVISA Advanced-Therapy Framework and RDC 505/2021

#### 3.2.1. Legislative and Institutional Foundations

Brazil’s regulatory framework for advanced-therapy medicinal products (ATMPs) is administered by the Agência Nacional de Vigilância Sanitária (ANVISA) and is among the most structurally developed CGT regulatory systems in Latin America, having been explicitly modeled on elements of the EU ATMP framework while remaining earlier in overall maturity relative to the FDA and EMA. ANVISA’s role as the principal architect of CGT regulation in Brazil was already documented in 2015, when the foundational regulatory analysis identified persistent structural obstacles: constitutional and legislative constraints on the commercialization and patenting of human substances, jurisdictional complexity spanning up to four different institutions, and an unresolved classification raise the question of whether these products would be regulated under the assumptions adopted for drugs and biological products or for human blood and tissues [64]. These foundational tensions between Brazil’s constitutional framework and the requirements of commercial CGT regulation mark a structural divergence from the EU’s Regulation 1394/2007 and the FDA’s Section 351 BLA framework, both of which provide unambiguous primary legal authority for CGT as a biologic or medicinal product [32]. ANVISA issued Resolução RDC No. 204/2017 as the first regulatory framework specifically addressing ATMPs in Brazil, defining the category to include gene-therapy products, somatic-cell-therapy products, and tissue-engineered products, a taxonomy borrowed directly from EU Regulation 1394/2007 [65].

#### 3.2.2. Current Regulatory Architecture: RDC 505/2021, 506/2021, 508/2021, and IN 270/2023

RDC 204/2017 was substantially superseded in 2021–2023 by a four-instrument architecture: RDC 505/2021 (27 May 2021) establishes minimum ATMP registration requirements as the primary marketing-authorization pathway; RDC 506/2021 (same date) sets clinical trial rules, including CEP/CONEP ethics oversight and import/export authorization; and RDC 836/2023 with IN 270/2023 (both December 2023) complete the manufacturing-quality architecture, establishing GMP for human cells and supplementary ATMP-specific GMP requirements, respectively, together narrowing RDC 508/2021 to govern only conventional, non-ATMP cell therapy [66,67,68,69,70]. ANVISA describes this framework as adapting approval models to each product and population, incorporating accelerated analyses, emergency-use risk-control mechanisms, and rare-disease-focused monitoring that parallels, without fully replicating, the EMA’s Conditional Marketing Authorization (Section 2.2) [71].

A structural feature distinguishing Brazil’s framework from the FDA, EMA, and PMDA systems is the explicit, mandatory integration of long-term follow-up (LTFU) and adaptive pharmacovigilance requirements directly into the marketing-authorization framework for all ATMPs, alongside traceability obligations for starting materials, products, and patients. In 2023, comparative assessment of post-marketing safety and efficacy surveillance across Brazil, the EU, Japan, and the United States found that all four jurisdictions’ regulatory agencies have developed guidelines requiring post-marketing surveillance plans for ATMPs, but that the terminology and regulatory mechanisms used to impose these requirements differ by jurisdiction [71]. In the US system, by contrast, LTFU obligations are articulated through separate, product-specific REMS and post-marketing commitments rather than a uniform statutory requirement [37].

#### 3.2.3. Expedited Pathways and International Engagement

ANVISA operates a priority-review mechanism for ATMPs addressing serious diseases without existing therapeutic alternatives, with accelerated technical-review timelines available under the current framework. ANVISA does not yet operate a PRIME-equivalent designation offering prospective, iterative scientific advice comparable to the EMA mechanism described in Section 2.2 [34]. ANVISA was admitted as a full Regulatory Member of the International Council for Harmonization (ICH) in November 2016 alongside South Korea’s Ministry of Food and Drug Safety, as part of the ICH’s first cohort of new Regulatory Members following the 2015 reform of its governance structure, representing the most advanced BRIC-M integration into the ICH system [72,73]. Since accession, ANVISA has engaged in bilateral exchange with EMA and FDA reviewers through the ICH framework and has begun formal domestic adoption of selected ICH quality guidelines [74].

#### 3.2.4. Manufacturing Capacity and Convergence Readiness

Commercial-scale GMP-certified CGT manufacturing capacity in Brazil remains comparatively limited relative to the United States, the EU, and China, though the regulatory pathway itself is now mature. A 2025 review of CAR-T-cell manufacturing across Latin America found that Brazil has paired its regulatory pathway with accredited treatment centers and an emerging national platform for viral vector and plasmid production, positioning it, alongside Colombia and Mexico, among the region’s more advanced jurisdictions for domestic CGT manufacturing capability, even as cost, fragmented regulation in neighboring markets, and constrained manufacturing capacity continue to limit regional access overall [75]. Brazil, alongside China, is the most convergence-ready BRIC-M jurisdiction. ANVISA’s full ICH membership, its structurally developed and recently modernized ATMP framework, and its active participation in international regulatory-exchange programs provide a strong foundation for harmonization dialogue with the EMA and FDA. Remaining challenges include a limited technical workforce within ANVISA’s ATMP review division, tension between the ATMP framework and Brazil’s public healthcare financing infrastructure for high-cost therapies, and continued development of inspection capacity for complex advanced-therapy manufacturing environments consistent with EMA and FDA expectations [71].

### 3.3. Russia: Federal Law 180-FZ and the Biomedical Cell Product Framework

#### 3.3.1. Legislative and Institutional Framework

Russia established a dedicated legislative framework for cell-therapy products through Federal Law No. 180-FZ On Biomedical Cell Products, adopted on 23 June 2016 and effective from 1 January 2017 [76]. BCPs regulate development, pre-clinical and clinical trials, state registration, manufacture, sale, storage, transportation, use, and disposal, and include the donation of biological material for their production [76]. Minzdrav is the primary authority, with enforcement delegated to Roszdravnadzor [77]. Gene-therapy products are regulated separately under Federal Law No. 61-FZ of 12 April 2010 on the Circulation of Medicines, into which a definition of gene-therapy medicinal products was introduced by Federal Law No. 429-FZ of 22 December 2014, effective 1 July 2015 [78], creating a bifurcated architecture that partially parallels, without fully replicating, China’s dual-track system (Section 3.1). BCP trials must occur at Minzdrav-accredited institutions [79], narrowing the eligible site pool relative to jurisdictions without such accreditation requirements. Manufacturing operates under EAEU GMP standards; since January 2021, marketing authorization has been subject to the supranational EAEU framework (Eurasian Economic Commission Council Decision No. 78, 2016), adding a regional harmonization layer structurally distinct from the EMA’s centralized procedure [80,81]. The EAEU’s decentralized and mutual-recognition procedures are modeled partly on the EU approach but remain in early implementation, with pre-2021 national authorizations required to reach EAEU compliance by the end of 2025 [82].

#### 3.3.2. Expedited Pathways and the Absence of a Hospital Exemption Mechanism

A distinctive feature of Russia’s regulatory framework, by comparison with both the EU hospital exemption mechanism under Regulation 1394/2007 and the FDA’s minimal manipulation/homologous use pathway under 21 CFR Part 1271, is the principle of unified marketing-authorization requirements applying equally to autologous, allogeneic, and combined biomedical cell products. A comparative regulatory analysis by scientists at Russia’s Scientific Centre for Expert Evaluation of Medicinal Products found that the central distinguishing feature of Russian cell-therapy regulation is this principle of unified requirements, together with the absence of a hospital-exemption mechanism, present in many other jurisdictions, that permits the prescription and use of a personalized autologous medicine produced in a medical institution’s own laboratory for a specific named patient [83]. The same analysis found that priority-review mechanisms for cell-therapy products, such as accelerated assessment, accelerated approval, or conditional marketing authorization, are currently absent in Russia, given the novelty of the regulatory framework and the biological properties of these products [83].

#### 3.3.3. Clinical and Manufacturing Landscape

Russia possesses substantial academic research capacity in cell biology, molecular genetics, and hematology, but the clinical translation pipeline under the BCP framework remains comparatively early stage relative to the volume of underlying basic-science output [76,83]. Manufacturing capacity for CGTs is concentrated in a small number of academic research institutes and state biomedical enterprises operating under EAEU GMP certification; specialized inspection expertise for viral vector production and complex cell-manipulation environments is an area of continuing development. This constraint is not unique to Russia; Brazil (Section 3.2.4) and India face comparable challenges independently, and none of the three has been the subject of a published capacity assessment specific to advanced-therapy manufacturing. One verifiable indicator of inspection-system integration is participation in the Pharmaceutical Inspection Co-operation Scheme: ANVISA became a participating authority on 1 January 2021, whereas neither Roszdravnadzor nor CDSCO currently participates [84].

#### 3.3.4. Convergence Readiness and Harmonization Trajectory

Russia’s convergence trajectory is the most constrained among the BRIC-M jurisdictions surveyed in this review. Russia participates in the International Council for Harmonization only as an Observer, through Roszdravnadzor (the Federal Service for Surveillance in Healthcare), and holds no Regulatory Member status; neither the Eurasian Economic Union nor the Eurasian Economic Commission holds ICH status. Russia is therefore the least ICH-integrated of the BRIC-M jurisdictions, in contrast to Brazil’s ANVISA, China’s NMPA, and Mexico’s COFEPRIS, all of which hold full Regulatory Member status [73]. This limits the routes through which ICH technical standards enter Russian regulatory practice. Russia does, however, participate in some multilateral regulatory fora: Roszdravnadzor is a member of the International Pharmaceutical Regulators Program [85]. The more immediate path toward regional regulatory convergence runs through continued development of the EAEU’s common pharmaceutical market, which has already introduced harmonized marketing-authorization pathways modeled in part on the EU approach, operating in parallel with selective Russian reference to ICH quality and safety standards even absent formal ICH membership [82,86]. Direct engagement between Russia and the FDA-EMA bilateral convergence mechanisms described in Section 7 is not presently a realistic near-term pathway; any future inclusion of Russia in a broader Global CGT Regulatory Convergence Framework would more plausibly proceed through engagement with the EAEU as a regional proxy structure than through direct bilateral channels.

### 3.4. India: CDSCO, ICMR, and the Emerging CGT Regulatory Infrastructure

#### 3.4.1. Legislative and Institutional Framework

India’s regulatory framework for CGTs is administered primarily by the Central Drugs Standard Control Organization (CDSCO) under the Drugs and Cosmetics Act 1940 and its New Drugs and Clinical Trials (NDCT) Rules 2019, supported by the Indian Council of Medical Research (ICMR) for clinical trial ethics and quality standards [87]. The CDSCO, operating under the Ministry of Health and Family Welfare, is the primary drug regulator; the Department of Biotechnology (DBT), under the Ministry of Science and Technology, separately oversees gene-therapy research through the Review Committee on Genetic Manipulation (RCGM) [88].

This bifurcated governance structure is a defining feature of India’s CGT regulatory landscape. A product involving genetic manipulation falls under DBT/RCGM oversight during research and early development, then transitions to CDSCO jurisdiction for clinical trials and marketing authorization. The multi-institutional approval pathway, which may require sequential authorization from the institutional biosafety committee, RCGM under DBT, the Gene Therapy Advisory and Evaluation Committee (GTEAC) established in 2019, ethics committees, and the CDSCO, creates potential ambiguity in pre-IND interactions, risk-based product classification, and the definition of applicable manufacturing standards [88,89]. Resolving this jurisdictional split and consolidating CGT regulatory authority is identified in the Indian scientific literature as the most important structural reform needed to advance regulatory readiness [88].

#### 3.4.2. Expedited Approval Pathways, the Phase-Lag Policy, and Clinical Landscape

The NDCT Rules 2019 classify substantially manipulated or genetically engineered products as new drugs and include accelerated-review provisions for conditions of serious or unmet medical need [87]. In practice, however, India’s regulatory framework for CGTs remains at an early developmental stage, with the principal constraints lying in execution and enforcement rather than in the rules themselves [90,91]. No formal Breakthrough Therapy, RMAT, or PRIME-equivalent designation exists for CGTs specifically, and the accelerated pathway created under the NDCT Rules has so far been applied almost exclusively to small molecules and conventional biologics; no CGT product has yet reached full marketing authorization through that accelerated route specifically. Dedicated CGT technical guidance does exist, though it remains fragmented across advisory and draft instruments rather than consolidated into a binding framework. The Indian Council of Medical Research and the Department of Biotechnology issued National Guidelines for Gene Therapy Product Development and Clinical Trials in 2019, which prohibit germline modification, restrict research to somatic-cell therapy and mandate long-term follow-up, but are advisory and operate alongside the legally binding NDCT Rules [92]. The CDSCO released draft guidelines specific to CAR-T therapies in 2024, addressing starting-material characterization, manufacturing controls, quality testing, and post-market surveillance. Institutionally, the Review Committee on Genetic Manipulation (RCGM), under the Department of Biotechnology, holds statutory oversight of genetically modified organisms, and its approval is mandatory before CAR-T clinical activity may proceed, while the Gene Therapy Advisory and Evaluation Committee (GTEAC) was proposed under the 2019 guidelines as an apex technical body advising the CDSCO’s Subject Expert Committee. What remains absent is a consolidated, binding CGT-specific technical framework covering CMC, potency assay development, and comparability. A direct input from the CDSCO delivered at the 7th Asia Partnership Conference on Regenerative Medicine (April 2024) clarified the agency’s current posture: India requires local clinical data to support new drug approval and permits phase IV studies in lieu of additional local data for orphan indications or conditions of unmet medical need where the product is already approved in a reference jurisdiction, but has not established a formal reliance mechanism that aligns Indian regulatory decisions with those of foreign authorities [93].

A distinctive and long-standing feature of India’s clinical development environment for CGT is the so-called “phase-lag policy,” under which first-in-human studies historically required prior overseas safety data before the CDSCO would authorize an Indian trial, a direct expression of the reference-country reliance model discussed more broadly in Section 3.5. The NDCT Rules 2019 partially addressed this constraint by permitting waivers of Indian Phase I data for products already approved by specified reference regulatory authorities (the FDA, the EMA, the UK MHRA, Health Canada, the TGA, or the PMDA), although such waivers follow a full dossier review rather than a streamlined reliance procedure [87]. This reform trajectory, discussed extensively at a CDSCO/ICMR capacity-building workshop as part of India’s broader ambition to move from reference-country dependence toward independent early-phase CGT trial authorization, is treated in the Indian CGT regulatory literature as a concrete marker of the country’s evolving convergence practice [88,89].

Despite these structural constraints, India has produced its first domestically developed CGT product to reach the market. Unlike China, which has supported commercially available CAR-T therapies since 2021, India’s first domestic CGT marketing authorization was granted to talicabtagene autoleucel (NexCAR19; formerly actalycabtagene autoleucel), developed by ImmunoACT in partnership with IIT-Bombay and approved by the CDSCO on 13 October 2023 for relapsed/refractory B-cell non-Hodgkin lymphoma and relapsed/refractory B-cell acute lymphoblastic leukemia [93,94,95,96]. This approval is consistent with the regulatory position described above rather than in tension with it: NexCAR19 was authorized through the standard new drug route under the NDCT Rules 2019, not through the accelerated pathway, and the review drew on RCGM biosafety clearance, GTEAC technical input, and evaluation by the CDSCO’s Subject Expert Committee. The marketing authorization was granted by the CDSCO. Beyond this single approval, India’s overall CGT clinical trial portfolio remains substantially smaller than China’s, reflecting structural differences in trial-site preparedness, regulatory submission experience, and manufacturing infrastructure [88,89]. A capacity-building workshop convening the CDSCO, the ICMR, and academic and industry stakeholders has documented these persistent gaps and underscored the continuing need for policy harmonization and investment [9].

#### 3.4.3. Manufacturing Infrastructure

Manufacturing infrastructure for CGTs in India is nascent. The NDCT Rules 2019 require manufacturers to secure GMP facility approval and pass inspection before manufacturing test batches [87]. A small number of academic institutions have developed early-stage viral vector and cell-therapy manufacturing capabilities, but none currently operate at the scale or GMP maturity required for commercial supply [88]. The absence of a GMP-certified viral vector manufacturing ecosystem remains a significant constraint on India’s ability to serve as a manufacturing base or clinical trial site for advanced gene-therapy programs [89].

#### 3.4.4. Convergence Readiness and Harmonization Trajectory

India participates in the International Council for Harmonization as an Observer, with the CDSCO listed among the Legislative or Administrative Authorities; it does not hold Regulatory Member status [73]. The CDSCO has begun issuing guidance documents referencing ICH principles, but has not yet formally adopted ICH Q5, Q6B, Q10, or Q12, the quality guidelines most directly relevant to CGT CMC requirements as binding domestic standards [88]. A 2026 regulatory update from the Asia Partnership Conference reported that India has introduced new CDSCO rules to expedite treatments for areas with unmet medical needs, while the 2019 ICMR document titled “Evidence-Based Status of Stem Cell Therapy for Human Diseases” provides a framework for recommending approved indications [93]. Compared with Brazil and China, India presents the most structurally complex pre-submission pathway, the least developed manufacturing ecosystem, and the greatest distance from ICH guideline adoption among the BRIC-M jurisdictions with substantive CGT regulatory frameworks. The most consequential near-term structural reform would be the consolidation of CDSCO-DBT jurisdictional authority into a dedicated advanced-therapy oversight structure [88,89], modeled on the EMA’s Committee for Advanced Therapies (Section 2.2), which would reduce multi-institutional approval burden and provide a single point of scientific engagement for CGT sponsors.

### 3.5. Mexico: COFEPRIS and the 2025 vía Abreviada Reliance Pathway

#### 3.5.1. Legislative and Institutional Framework

COFEPRIS, operating under the Ministry of Health within the General Health Law and Health Supplies Regulation, was created in 2001 with a biotherapeutic framework aligned to the ICH CTD structure [97,98]. Mexico lacks a dedicated CGT classification, CMC guidance, or an expedited pathway. Biotechnology-derived medicines, including cell-based products, are regulated under Article 222 Bis of the General Health Law and its implementing standard, NOM-257-SSA1-2014. In 2024, COFEPRIS reported that a dedicated advanced-therapies framework remained under development [97,98] and was the most significant structural gap in Mexico’s framework [97,99]. According to a June 2025 account published by the Tecnológico de Monterrey science publication, Commissioner Rafael Hernández of COFEPRIS has stated publicly that “science goes one step ahead of regulation” and that regulatory adjustments are needed, indicating that COFEPRIS is close to completing a comprehensive framework to govern advanced medical therapies modeled on European precedents, with the EU’s ATMP regulatory architecture described in Section 2.2 as the principal international reference point [99]. The proposal had received approval from COFEPRIS’s legal departments and was technically ready for implementation as of mid-2025, but had been temporarily paused to align with the government’s broader “Plan México” pharmaceutical competitiveness initiative [99].

#### 3.5.2. The 2025 Abbreviated Regulatory Pathway and Its Relevance to CGTs

The Vía Abreviada, established by the COFEPRIS agreement (Diario Oficial, 18 July 2025; effective 1 September 2025), enables sponsors to leverage prior authorizations from designated Reference Regulatory Authorities (RRAs) and FDA-, EMA-, and WHO-listed authorities for drugs; IMDRF/MDSAP membership for devices is the primary evidentiary basis, with a maximum review of 45 business days for drugs (30 for devices), excluding emergency, accelerated, or conditional foreign approvals as qualifying references [100,101,102]. While not CGT-specific, this pathway has direct practical relevance for CGT sponsors: it codifies at the regulatory level the reference-country reliance model that had previously operated informally through the older “Equivalence Route” (previously limited to the FDA, Health Canada, and the PMDA only) and moves Mexico meaningfully closer to the convergence aspiration described in this review’s introductory aim [100,101]. For products such as tisagenlecleucel and axicabtagene ciloleucel, which hold FDA and EMA marketing authorizations obtained through standard procedures, the abbreviated pathway provides a structurally cleaner and faster route to COFEPRIS authorization than was previously available [97]. The critical caveat, however, is that COFEPRIS’s technical capacity for independent CMC and clinical review of novel CGTs, including domestically developed products without prior reference-country authorization, remains constrained by staffing and scientific expertise limitations not addressed by the reliance mechanism itself [99].

#### 3.5.3. Clinical Trial Environment and Manufacturing Infrastructure

Mexico’s pharmaceutical clinical trial sector has an established infrastructure anchored at the Instituto Nacional de Cancerología (INCan) and major academic medical centers, but CGT-specific clinical trial activity remains very limited. Patient access has been primarily through individual named-patient access or compassionate-use provisions rather than systematic CGT trial enrollment. Commercial-scale GMP-certified CGT manufacturing infrastructure is effectively absent in Mexico: there are no GMP-certified viral vector manufacturing facilities, and cell-therapy manufacturing is confined to basic academic laboratory settings, placing Mexico well behind Brazil, India, and China in manufacturing development among the BRIC-M group [97,99].

#### 3.5.4. Convergence Readiness and Harmonization Trajectory

COFEPRIS holds full ICH Regulatory Member status, placing Mexico in the same formal membership tier as Brazil’s ANVISA and China’s NMPA and ahead of India and Russia, both of which participate only as Observers [73]. Mexico’s convergence constraint is therefore not one of multilateral standing but of domestic technical capacity and the absence of a dedicated ATMP framework [100,101]. The most impactful near-term interventions would be, first, finalization of a dedicated ATMP regulatory framework modeled on EU Regulation 1394/2007, as COFEPRIS has indicated is its intent; second, domestic transposition of the ICH quality guidelines most directly relevant to CGT CMC, consistent with the obligations already attaching to Regulatory Member status; and third, technical assistance for COFEPRIS reviewer training through PAHO, the WHO and the ICH [73,99]. Mexico’s participation in PANDRH (the Pan American Network for Drug Regulatory Harmonization) provides a regional platform that could be leveraged to develop shared Latin American CGT regulatory standards, building on Brazil’s more developed ANVISA framework described in Section 3.2 [103]. In combination with Brazil’s full ICH membership and more complete ATMP infrastructure, a PANDRH-facilitated Latin American CGT regulatory convergence dialogue would represent a meaningful step toward the regional-level harmonization aspiration of this review.

### 3.6. The BRIC-M Convergence-Readiness Spectrum

**Figure 2:**
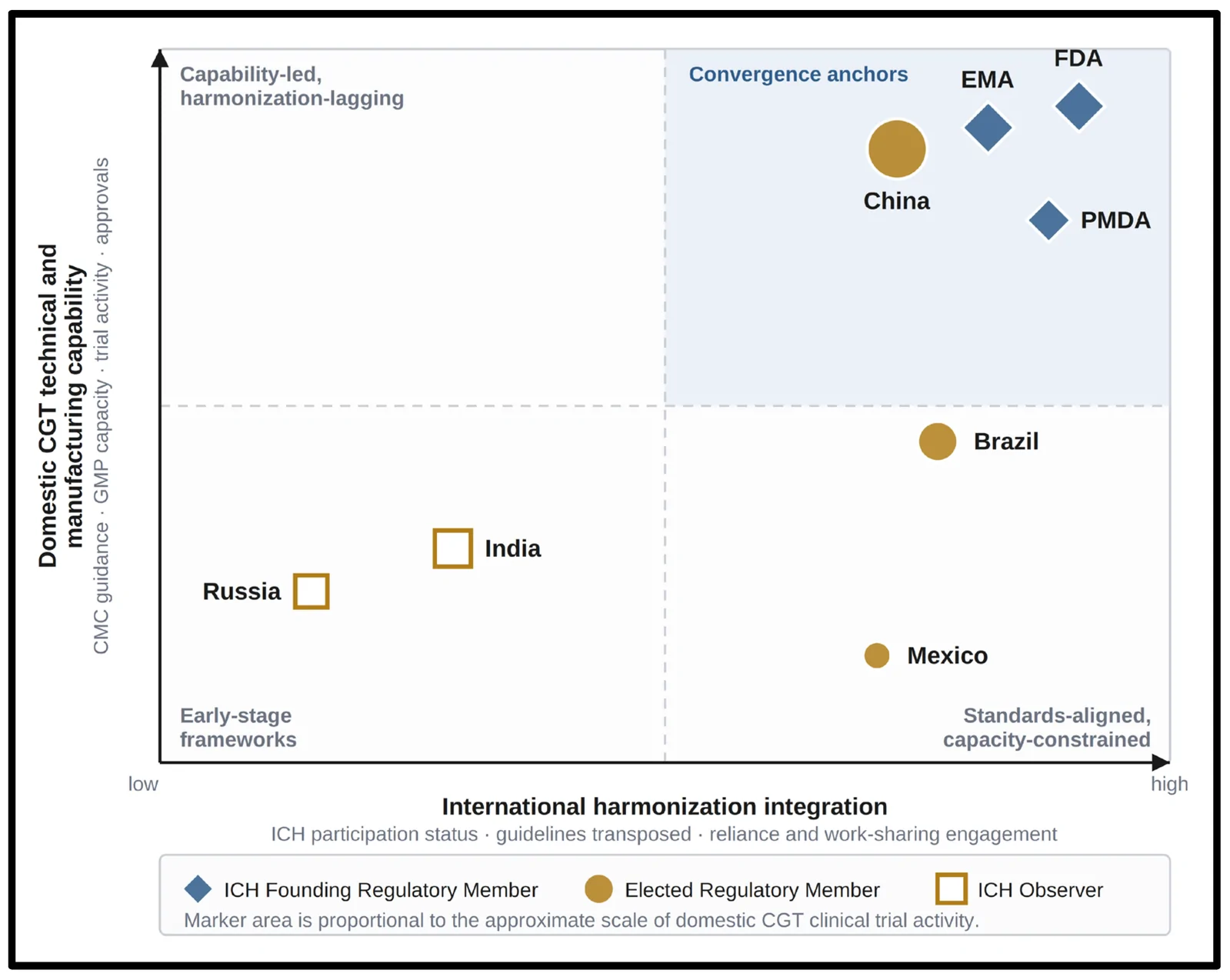
Convergence-readiness map positioning the eight jurisdictions examined in this review against two independent dimensions: international harmonization integration (horizontal) and domestic CGT technical and manufacturing capability (vertical). Marker shape denotes ICH participation status, marker size approximates the scale of domestic CGT clinical trial activity, and fill distinguishes established agencies from BRIC-M jurisdictions. The separation of the two axes clarifies why formal ICH membership alone is an insufficient indicator of Convergence Readiness: Mexico holds full Regulatory Member status yet lacks a dedicated advanced-therapy framework and commercial manufacturing capacity, whereas India possesses an emerging domestic capability without corresponding harmonization and integration. Positions reflect a qualitative assessment against the criteria defined in the footnote to Table 2 and are intended for comparative interpretation rather than as absolute measures.

As Table 2 and Figure 2 illustrate, the five BRIC-M frameworks span a wide regulatory spectrum that extends the FDA–EMA–PMDA continuum established in Section 2.4. China is the most convergence-ready jurisdiction, with full ICH membership, explicit 2025 CMC guidance benchmarked against FDA/EMA standards, and the largest BRIC-M CGT pipeline, with post-marketing surveillance maturity as its principal remaining gap [41,53,56,63]. Brazil has built the most structurally complete non-China ATMP framework, with primary legislation, GMP, and mandatory long-term follow-up all in place, though manufacturing scale and technical-workforce constraints persist [64,71,72,74]. Russia has a dedicated cell-product statute and an EAEU harmonization layer but lacks expedited pathways and ICH membership [76,80,82,83]. India remains in the earliest stage, prioritizing first-in-human trial authorization and CDSCO–DBT jurisdictional consolidation over marketing-pathway development [87,88,89,93]. Mexico relies most heavily on reference-country reliance and has no dedicated CGT framework, though its 2025 Abbreviated Pathway is a meaningful step toward systematic reliance [97,99,100,101]. Consistent with Cai et al.’s comparative post-marketing surveillance analysis, the gap between established and emerging markets is most pronounced in post-approval surveillance, CMC lifecycle management, and potency assurance rather than in basic approval-pathway architecture [41], the specific gaps examined comparatively in Section 4.

### 3.7. Highly Mature CGT Regulators Outside the Study Frame

The purposive sample described in Section 3 does not include several jurisdictions whose CGT frameworks are among the most developed internationally. Their omission reflects the sampling rationale rather than any judgement about their significance, and a brief account is warranted. The Republic of Korea regulates advanced therapies under the Act on the Safety of and Support for Advanced Regenerative Medicine and Advanced Biological Products, enacted in August 2019 and implemented in August 2020, which established a dedicated approval route and a conditional approval mechanism [104]. Singapore regulates cell-, tissue- and gene-therapy products under the Health Products (Cell, Tissue and Gene Therapy Products) Regulations 2021, in force from 1 March 2021, applying a two-class risk framework that distinguishes minimally manipulated products for homologous use from all other products [105,106]. Both the MFDS and the HSA hold WHO Maturity Level 4 and were among the first three authorities designated as WHO-Listed Authorities in October 2023 [52], making them the most convergence-ready CGT regulators outside the ICH founding members. Canada regulates CGTs as Schedule D biologic drugs under the Food and Drugs Act and Food and Drug Regulations, with the Notice of Compliance with conditions pathway available for products addressing serious conditions; the Agile Regulations amendments, in force from 1 July 2025, restructured the biologics requirements [107]. Australia has regulated biologicals under a dedicated four-class, risk-based framework within the Therapeutic Goods Act 1989 since 31 May 2011 [108]. The United Kingdom has licensed ATMPs for Great Britain independently since 1 January 2021 under the Human Medicines Regulations 2012 as amended, supported by the Innovative Licensing and Access Pathway, which integrates regulatory and health technology assessment input from an early stage [109]. The UK has also introduced the first dedicated framework for point-of-care and modular manufacture of medicines, in force from 23 July 2025 [110]. These five jurisdictions would be natural early participants in the convergence architecture proposed in Section 8. Korea and Singapore in particular, as Maturity Level 4 authorities with dedicated CGT legislation, would qualify for the highest participation tier without transitional support.

**Table 2:** Comparative CGT regulatory framework characteristics across the BRIC-M jurisdictions (Brazil, Russia, India, China, and Mexico), organized across six regulatory domains and stated as of 30 June 2026. Maturity descriptors are defined in the footnote below.

Regulatory Dimension	China (NMPA/CDE)	Brazil (ANVISA)	Russia (Minzdrav/Roszdravnadzor)	India (CDSCO/DBT/ICMR)	Mexico (COFEPRIS)	Legal Status
**(A) Legislative and Institutional Foundation**						
**Primary regulatory authority**	NMPA (est. 2018); technical review by CDE; reference standards by NIFDC [53]	ANVISA; framework modeled on EU ATMP Regulation 1394/2007 [64,72]	Minzdrav (Ministry of Health); enforcement via Roszdravnadzor [76,77]	CDSCO (trials and marketing authorization); DBT/RCGM (gene-therapy R&D); ICMR (ethics and standards) [87,88]	COFEPRIS (Secretaría de Salud) [97,98]	National legislation (binding)
**Dedicated CGT legislative category**	Established distinct biologics subcategory under DAL 2019; MAH framework introduced [53,54]	Established ATMP category defined (RDC 204/2017), superseded by RDC 505/2021 [65,66]	Developing Biomedical Cell Products (BCPs) under Law 180-FZ; gene therapy regulated separately under Law 61-FZ (2015 amendment) [76,111]	Developing CGTs classified as “New Drugs” under NDCT Rules 2019; no dedicated sub-category [87,88]	None; regulated under the general biologics framework (Art. 222 Bis, General Health Law; NOM-257-SSA1-2014) [97,98]	National legislation (binding)
**Primary enabling instrument(s)**	DAL 2019; NMPA Order No. 27 (2020); CDE guidance series (2017–2025) [53,54,55]	RDC 505/2021 (registration); RDC 506/2021 (clinical trials); RDC 836/2023 and IN 270/2023 (GMP) [66,67,68,69]	Law 180-FZ (BCPs); Law 61-FZ (gene therapy); EAEU Council Decision No. 78 [76,80,111]	Drugs and Cosmetics Act 1940; NDCT Rules 2019; sequential CDSCO–DBT–RCGM–GTEAC approval [87,88,89]	General Health Law; NOM-257-SSA1-2014; Vía Abreviada (DOF, 18 July 2025; effective 1 September 2025) [98,100]	National legislation (binding)
**Dual track/parallel oversight**	Established—IIT (NHC) + formal IND (NMPA) tracks; both can lead to commercial approval [54]	Developing—CEP/CONEP ethics + CTNBio biosafety (GMOs) + ANVISA: multi-institution but consolidated [66,67]	Absent-unified BCP track under Law 180-FZ; gene therapy via the separate Law 61-FZ route [76,111]	Established—CDSCO (MA) + DBT/RCGM (early development) + GTEAC (gene editing) + ethics committees: highly fragmented [88,89]	Absent single COFEPRIS authorization route; no parallel biosafety or advanced-therapy oversight body [97,98]	National legislation (binding)
**(B) Expedited Approval Pathways**						
**Breakthrough/priority designation**	Established—BTD (2020) + Priority Review + Conditional Approval: 8 CAR-T + 2 AAV-GT products received BTD by end of 2022 [53,56]	Developing—Priority Review for rare diseases without alternatives; no PRIME-equivalent iterative advice [66,71]	No expedited designation; standard review only [111]	No RMAT/PRIME/Sakigake equivalent; accelerated pathway under NDCT 2019 not yet applied to any CGT [87,93]	None; 2025 Vía Abreviada not CGT-specific and requires existing standard RRA approval [100,101]	Administrative scheme (non-binding)
**Conditional/accelerated approval mechanism**	Established—Conditional Approval on surrogate endpoints with mandatory confirmation; Axi-cel + Relma-cel conditionally approved 2021 [30,53]	Established—accelerated regulatory analyses for rare diseases; emergency-use risk-control mechanisms [66]	No conditional -approval mechanism; unified full-MA requirement [111]	Developing—NDCT 2019 accelerated pathway for serious/unmet-need conditions; untraversed by any CGT [80,86]	Developing—Vía Abreviada: 45-business-day review, requires prior standard RRA authorization [100,102]	National legislation (binding)
**Reference-country reliance mechanism**	Developing—MRCT provisions; foreign data accepted with domestic bridging; orphan framework underdeveloped [53]	Developing—ICH CTD format accepted; no formal reliance abridgement [66,68]	Absent at national level; no reference-country reliance pathway; regional mutual-recognition and decentralized procedures operate only within the EAEU common pharmaceutical market [80,82]	Developing—NDCT 2019 waiver of Indian Phase I data for products approved by FDA/EMA/MHRA/Health Canada/TGA/PMDA; full dossier review still required [80,87]	Established—Vía Abreviada codifies reliance on FDA/EMA/WHO-listed RRAs; replaces informal Equivalence Route [91,93,94]	National legislation (binding)
**(C) Clinical Trial Environment**						
**First-in-human (FIH) authorization**	Established—NMPA IND- or NHC-regulated IIT; large GCP-certified network (public/private) [53,54]	Established—under RDC 506/2021; CEP/CONEP + CTNBio (GMOs) [63]	Developing—Minzdrav-accredited institutions only; narrows eligible site pool [76]	Developing—CDSCO authorization required; historical phase-lag policy reforming; multi-institutional sequential approval [80,87]	No CGT FIH framework; access via individual compassionate use [88,90]	National legislation (binding)
**CGT clinical trial activity**	Largest BRIC-M portfolio. An analysis of 1580 CAR-T trials registered on ClinicalTrials.gov through April 2024 found China leading in cumulative registrations, although Chinese registrations declined between 2022 and 2023 while United States registrations rose steadily; because that analysis excludes the Chinese Clinical Trial Registry, it is likely to understate Chinese activity. Activity is concentrated in hematological malignancies and, increasingly, solid tumors [61,62].	Emerging; academic-led CAR-T; growing national viral vector/plasmid platform [71,75]	Limited; early-stage academic pipeline under BCP constraints [76,83]	Low but growing; NexCAR19 (talicabtagene autoleucel) and anti-CD19 CAR-T (NHL) received CDSCO MA in 2023; India’s first indigenously developed CAR-T trials [89]	Very limited; primarily named-patient access; no established trial ecosystem [97,99]	Not applicable
**(D) CMC Requirements and Manufacturing Infrastructure**						
**Dedicated CGT CMC guidance**	Established—CAR-T GMP, viral vector characterization, in vivo GT CMC guidance; 2025 draft (3-tier risk classification, benchmarked to ICH Q5E/FDA 2023/EMA ATMP Q&A) [54,55]	Established—GMP for human cells: RDC 836/2023; supplementary ATMP GMP: IN 270/2023 [68,69]	Developing—EAEU GMP standards (EU-modeled) for BCPs; no CGT-specific CMC guidance [80,81]	None; GMP facility approval/inspection required under NDCT 2019 [80,82]	None; general NOM-257-SSA1-2014 biologic requirements apply [98]	Final guidance (non-binding)
**Commercial-scale CGT manufacturing infrastructure**	Established—major CDMOs (e.g., WuXi Advanced Therapies); GMP-certified viral vector + cell-therapy manufacturing at commercial scale [30,53]	Developing—accredited academic centers; emerging national viral vector/plasmid platform; no commercial-scale GMP yet [37,66]	Developing—state biomedical enterprises, EAEU GMP-certified; limited viral vector inspection capacity; geopolitical constraints since 2022 [34,111]	Nascent; early-stage academic viral vector/cell-therapy capability only; none at commercial GMP scale [81,82]	Effectively absent; no GMP-certified viral vector manufacturing; cell therapy confined to basic academic settings [88,89]	National legislation (binding)
**(E) Post-Marketing Surveillance and Long-Term Follow-Up (LTFU)**						
**LTFU and PMS requirements**	Developing—LTFU required (variable duration); general PMS regulations, lack CGT-specific guidance primary gap vs. EU/US/Japan [54]	Established—mandatory LTFU + adaptive pharmacovigilance integrated for all ATMPs; traceability obligations (materials, products, patients) [41,66]	No CGT-specific LTFU; general EAEU pharmacovigilance framework applies [80,81]	No CGT-specific LTFU/PMS guidance; general drug PV applies; guidance under development [87,88]	No CGT-specific LTFU or PMS framework [88]	National legislation (binding)
**CGT products approved (to mid-2026)**	Established—≥6 domestic commercial CGTs (incl. Relma-cel; BMS-licensed Axi-cel); 2 conditionally approved 2021 [30,50]	Developing—ATMP MA framework operational; no domestically developed CGT fully authorized as of mid-2026 [56,66]	No BCP has completed full registration under Law 180-FZ to date [76,83]	Established—talicabtagene autoleucel (NexCAR19), the first domestically developed CGT, authorized by CDSCO on 13 October 2023 for relapsed/refractory CD19-positive B-cell lymphoma and B-cell acute lymphoblastic leukemia; hemophilia and thalassemia gene-therapy trials ongoing [94,95,96]	Developing—tisagenlecleucel and axicabtagene ciloleucel accessible via reference-country mechanism; no domestically authorized CGT [97,100]	Statute (binding)
**(F) International Harmonization and Convergence Readiness**						
**ICH membership/status**	Regulatory Member (2017); ICH Management Committee 2018, 2021 and 2024; 46 guidelines implemented by 2021 [53,63]	Regulatory Member (November 2016; first cohort of new members following the 2015 ICH governance reform) [72,73]	Observer participates through Roszdravnadzor; no Regulatory Member status; selectively references ICH standards via the EAEU GMP framework [73,80]	Observer—CDSCO listed among Legislative or Administrative Authorities; ICH quality guidelines not yet transposed as binding domestic standards [73,88]	Regulatory Member—COFEPRIS holds full ICH Regulatory Member status; NOM-257-SSA1-2014 is CTD-aligned, but domestic transposition of ICH quality guidelines remains incomplete [73,98]	(Binding on transposition)
**Regional harmonization framework**	IPRP participation; FDA-NMPA bilateral exchange (Jan 2025); WHO collaboration [30,63]	ICH Forum; Brazil–EU Regulatory Cooperation Agreement; PANDRH [72,103]	EAEU common pharmaceutical market (Russia, Belarus, Kazakhstan, Armenia, Kyrgyzstan); IPRP member; no PIC/S participation [80,82,85]	7th Asia Partnership Conference on Regenerative Medicine (Apr 2024); ICMR capacity-building program; partial IPRP participation [87]	PANDRH participation; 2025 Vía Abreviada expands recognized RRAs (FDA-, EMA-, WHO-listed authorities) [100,103]	Administrative scheme
**Overall convergence readiness and recommended GCRC-F role**	High—full-convergence partner: ICH member; explicit 2025 CMC alignment with FDA/EMA; largest BRIC-M pipeline; primary gap is PMS maturity [30,53,54,55]	High—full convergence partner: full ICH member; most structurally complete non-China ATMP framework; primary gaps are manufacturing scale and technical workforce [56,66,69,71]	Low—observer via EAEU proxy: dedicated BCP statute; EAEU harmonization layer; no expedited pathways; not an ICH member; geopolitical constraints limit bilateral engagement [76,80,82,83]	Medium—convergence candidate (structural reform support needed): ICH observer; first approved CGT (2024); critical reform priority is CDSCO-DBT jurisdictional consolidation [87,88,89,93]	Low—technical-assistance recipient: full ICH Regulatory Member but no dedicated CGT framework and limited independent CMC review capacity; the 2025 Vía Abreviada is a pragmatic reliance step; the EU ATMP framework is the principal reference for pending legislation [73,98,99,100]	Administrative scheme

Legal status classification: Statute or Regulation denotes binding primary legislation (for example, the FD&C Act, Regulation (EC) No. 1394/2007, or the PMD Act); CFR denotes binding US federal regulation; Final guidance denotes non-binding agency guidance representing current thinking, which under 21 CFR 10.115(d) does not establish legally enforceable responsibilities; Draft guidance denotes guidance issued for comment and not yet operative; Reflection paper denotes a non-binding scientific position. ICH guidelines are not directly binding and acquire effect only upon transposition by each regional authority.

## 4. Core Product-Level Scientific and Regulatory Challenges in CGT Development

**Figure 3:**
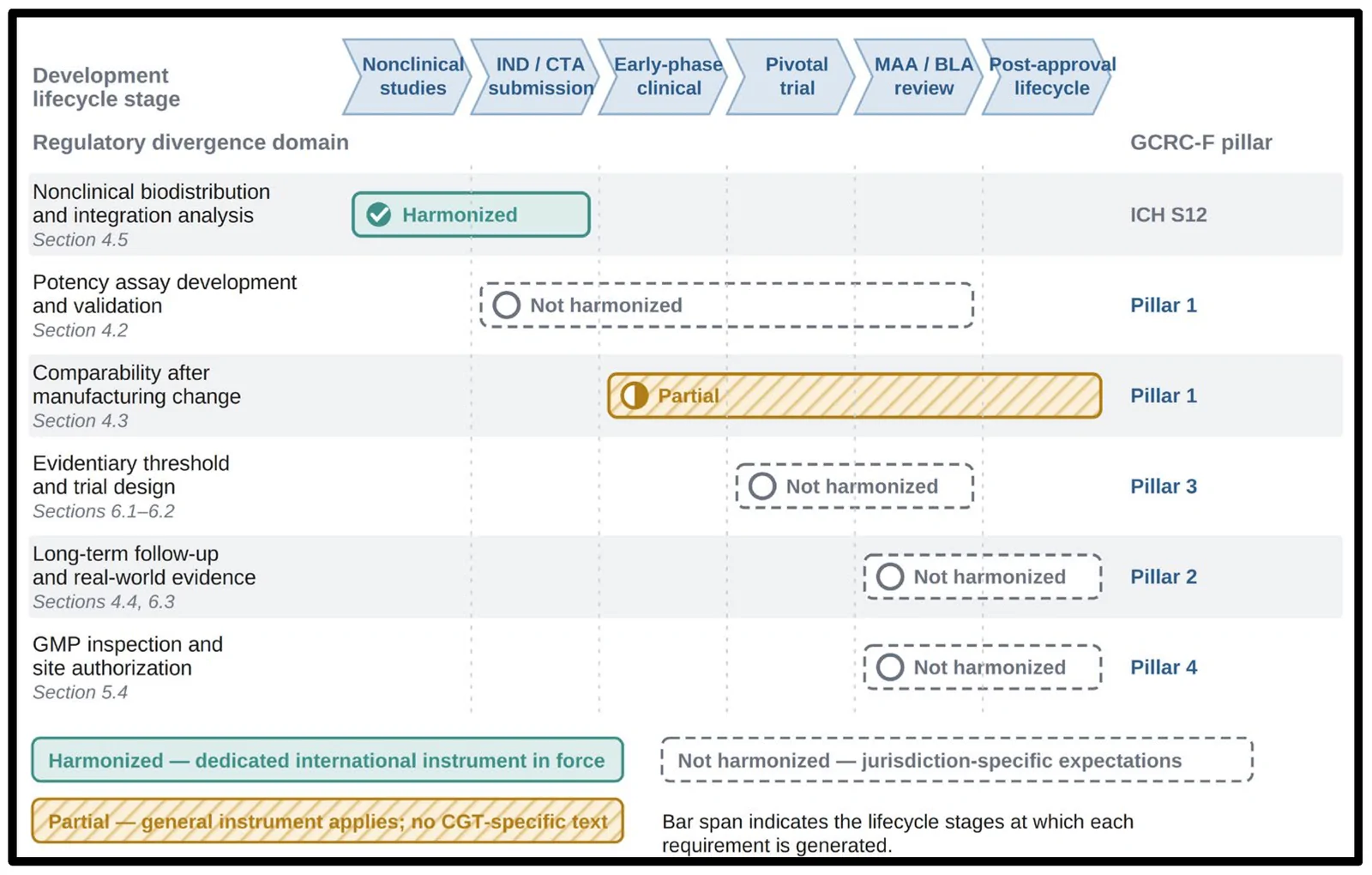
Regulatory divergence points mapped across the cell- and gene-therapy development lifecycle. Each horizontal bar spans the development stages at which the corresponding requirement is generated, and its fill state indicates the extent of international harmonization achieved to date: harmonized (solid), partial (half-toned), or not harmonized (outlined). Nonclinical biodistribution is the only domain for which a dedicated harmonized instrument exists (ICH S12, Step 5, 2023); every domain arising at or after first-in-human administration remains subject to jurisdiction-specific expectations. The right margin maps each divergence domain to the pillar of the proposed Global CGT Regulatory Convergence Framework that addresses it (Section 8). Agency-level detail for each domain is given in Table 3. BLA, Biologics License Application; CTA, clinical trial application; GMP, good manufacturing practice; IND, Investigational New Drug; LTFU, long-term follow-up; MAA, marketing authorization application; RWE, real-world evidence.

Despite remarkable clinical advances, the regulatory oversight of CGTs remains burdened by a set of intrinsic technical and scientific challenges that do not arise, or arise with lesser complexity, in the development of conventional small-molecule drugs or standard biologics. These challenges span the entire product lifecycle, from early-phase CMC characterization through post-approval safety surveillance, and have a direct bearing on the pace, cost, and global harmonization of regulatory review. The five domains addressed below represent the most consequential and least-resolved areas for developers operating across multiple jurisdictions (Figure 3), and Table 3 compares agency-level expectations across each of these five domains.

### 4.1. CMC Complexity and Batch-to-Batch Variability in Autologous Products

CGTs impose CMC challenges that are qualitatively different from those of recombinant protein biologics. Autologous products (e.g., CAR-T) are manufactured patient by patient, so starting material inherently varies in cellular composition, activation state, and functional capacity with disease stage, treatment history, and immune status [112,113]. Downstream manufacturing processes, cell activation, viral transduction, expansion, and formulation must each be sufficiently robust to absorb this upstream variability without propagating it into the final drug product. In autologous manufacturing, batch-to-batch inconsistency is an expression of donor biology rather than a manufacturing deviation; release specifications must nonetheless define meaningful thresholds for identity, purity, viability, and potency, and the regulatory difficulty lies precisely in setting limits that discriminate process failure from biological range [113]. For viral vector-based gene therapies, whether administered in vivo (e.g., AAV serotypes) or used ex vivo to transduce hematopoietic stem cells or T cells, the manufacturing challenges are similarly multifactorial. Scale-up of viral vector production from research-grade to GMP-compliant commercial batches introduces changes in cell culture conditions, transfection efficiency, and downstream purification that affect critical quality attributes (CQAs), including genome titer, vector genome integrity, empty-to-full capsid ratio, host-cell protein (HCP) content, and residual DNA [114,115]. High raw-material and process variability, difficulty characterizing complex biological materials, and gaps in understanding critical process parameters are the defining manufacturing challenges, with multivariate data analysis offering an underutilized framework for process understanding [113].

Regulatory agencies require that CQAs be defined and controlled within validated release specifications, in accordance with ICH Q8(R2) [116] and ICH Q9 principles and the FDA’s 2020 CMC guidance for gene-therapy INDs [17]. However, the inherent biological complexity of CGT products, particularly autologous cell therapies in which the “active ingredient” is a dynamic, heterogeneous cell population, means that standard analytical method validation frameworks developed for biologics cannot always be directly transposed. The GMP manufacture of personalized ATMP batches, as illustrated by Kropp et al. in the context of a non-virally engineered cell product for age-related macular degeneration [112], underscores the need for extended qualification strategies that specifically address low batch numbers, short production timelines, and batch-to-batch variability driven by donor heterogeneity. Regulatory alignment on acceptable variability thresholds and their statistical treatment across the FDA, EMA, and PMDA remains incomplete.

### 4.2. Potency Assay Development, Validation, and the Matrix Approach

Potency, defined by all major regulatory agencies as a quantitative measure of biological activity linked to the intended mechanism of action (MOA) of the product, is widely regarded as the most scientifically and technically demanding CQA for CGT products [117]. The FDA’s 2011 guidance on potency tests for cellular- and gene-therapy products [118] and the EMA’s 2016 guideline on potency testing of cell-based immunotherapy products [119] both mandate that potency assays be MOA-reflective, quantitative, and validated for use as a lot-release criterion. In practice, however, the biological mechanisms underpinning many CGT products are incompletely understood at the time of initial clinical development, rendering the design of a definitive MOA-based assay an aspirational rather than an immediately achievable standard [117].

The field has responded pragmatically through matrix or surrogate potency strategies, in which a panel of orthogonal assays encompassing functional, biochemical, and cell-based endpoints collectively represents product potency in the absence of a single validated MOA assay. For CAR-T products, as illustrated by Torrents et al. in their analysis of approved ATMPs, including tisagenlecleucel (Kymriah^®^), such matrices typically combine antigen-specific cytotoxicity assays, cytokine-release measurements, and CAR transduction efficiency, with each component contributing to a composite potency assessment [120]. For AAV-based gene-therapy products, in vivo potency assays assessing vector DNA delivery, transgene mRNA expression, and functional protein output in a rodent model remain the regulatory standard where validated in vitro correlates are unavailable, as demonstrated by De et al. [121] using an AAVrh.10-based model.

A critical unresolved challenge is the absence of internationally harmonized standards for potency assay development, qualification, and validation. A 2023 regulatory-science review of potency testing across cell- and gene-therapy products identifies embedding potency assays into early process development, and linking analytical outputs to demonstrable clinical relevance, as defining priorities for the field [122]. FDA-EMA divergence persists in the timing and stringency of validation expected across clinical phases. FDA permits greater early-phase flexibility, whereas potency-assay validation adequacy has been identified as a recurrent objection within the EMA-centralized review of ATMPs, consistent with the more prescriptive validation expectations set out in the EMA potency-testing guideline [119] and with published comparative analyses of ATMP approval pathways in the European Union and the United States [123]. A systematic quantification of objection categories across European Public Assessment Reports for authorized ATMPs has not, to our knowledge, been published, and would be a valuable contribution to the evidence base for potency harmonization. The PMDA’s expectations align broadly with those of the EMA but impose additional qualification specificity for biological raw materials used within potency assay systems, consistent with the adventitious-agent and human-derived-material controls set out in PMDA Notification No. 0709-2 on ensuring the quality and safety of gene-therapy products [45].

### 4.3. Comparability Following Manufacturing Change: The Limits of ICH Q5E

Manufacturing changes are inevitable in the lifecycle of any CGT product, spanning process scale-up from clinical to commercial production, equipment platform transitions, site transfers, and post-approval optimizations. For conventional biologics, comparability following such changes is evaluated within the framework established by ICH Q5E [5], which requires a risk-stratified comparative assessment of pre- and post-change product quality, supported by analytical, functional, nonclinical, and, where necessary, clinical data. CGT products present comparability challenges that strain the limits of this framework.

For AAV gene therapies, scale-up from transiently transfected HEK293 cell systems at research scale to baculovirus–insect cell platforms or suspension bioreactor systems at commercial scale can alter the stoichiometry of full versus empty capsids, the ratio of genome-containing to genome-deficient particles, and the pattern of capsid protein post-translational modifications, all attributes with potential functional relevance that may not be fully captured by standard release testing [114,115]. Hayes and Dobnik [115] have highlighted the inadequacy of simple empty: full capsid ratios as a sole integrity metric, recommending multiplex digital PCR (dPCR) and sedimentation velocity analytical ultracentrifugation (SV-AUC) as fit-for-purpose assays that more robustly characterize AAV drug product integrity, a perspective increasingly reflected in FDA reviewer expectations.

For cell-therapy products, phenotypic comparability, confirming that the post-change cell product retains the differentiation status, functional repertoire, and exhaustion profile of the pre-change material, cannot be fully addressed by the biochemical and physicochemical panels appropriate for protein biologics. Blümel et al. [124] describe a risk-based, patient-centric framework for biopharmaceutical comparability assessment that encompasses holistic data evaluation and regulatory submission strategy, but acknowledge that its application to living cell drug substances requires further methodological development. Webster and Woollett [125] have similarly argued for a global reference comparator framework to reduce duplicative comparability exercises across jurisdictions, a proposal directly relevant to CGT developers managing multi-regional programs.

Cross-jurisdictional divergence in comparability expectations adds regulatory complexity: the FDA’s change notification framework, categorized under post-approval supplements (prior approval, changes being effected in 30 days, and annual reports), differs meaningfully from the EMA’s variation classification (Type IA, IB, II variations) under Regulation (EC) No. 1234/2008 [46]. The PMDA’s change-management requirements for regenerative medical products under the PMD Act impose site-specific validation obligations that extend beyond, and are not fully reconciled with, the comparability principles of ICH Q5E [5,45]. In the absence of internationally harmonized comparability guidance specific to CGTs, developers must design jurisdiction-tailored comparability packages, a redundancy that increases development costs without proportionate safety or scientific benefits.

### 4.4. Long-Term Follow-Up: Divergent Durations, Endpoints, and Data Platforms

The novel and, in many cases, permanent nature of CGT interventions involving integration of therapeutic genetic material into the patient genome, long-term transgene expression, or durable modification of hematopoietic or immune-cell populations mandates post-approval long-term follow-up (LTFU) of treated patients to characterize the delayed safety profile of these modalities. Regulatory LTFU frameworks impose significant operational and financial obligations on developers and diverge substantially in their structure and evidentiary requirements across agencies.

The FDA’s 2020 LTFU guidance defines risk-stratified follow-up periods of up to 15 years for integration-capable products (retroviral/lentiviral vectors, AAV) or genomic editing, with minimum observations spanning survival, disease status, new malignancies, autoimmune sequelae, neurological events, and (for germline-capable modalities) reproductive outcomes [37]. The Strimvelis registry for ADA-SCID patients established an early operational model for 15-year LTFU via a patient-centric electronic registry, demonstrating both the feasibility of long-term monitoring in small orphan populations and the logistical challenge of patient retention over extended windows [126]. The longest published systemic AAV LTFU dataset, a 12–15-year follow-up of the first intravascular AAV2-hFIX16 hemophilia B trial, showed no hepatocellular carcinoma, sustained hepatic toxicity, or other major delayed adverse events, providing early reassurance on long-term AAV safety [127]. However, low-frequency insertional mutagenesis in neonatal mouse models, potential clonal expansion of transduced hepatocytes over decades, and detection of AAV sequences in human tumor specimens in large epidemiological studies [128] have maintained regulatory vigilance around long-term oncogenic risk, a concern ICH S12’s biodistribution/integration-site framework addresses but cannot fully resolve with currently available follow-up data [8].

The EMA embeds LTFU within product-specific RMPs rather than a universal time-defined framework, yielding heterogeneous protocols across approved ATMPs and complicating cross-product evidence aggregation [38]. The PMDA’s post-conditional confirmatory study requirements under the ASRM function as LTFU mechanisms but remain product-specific and unaligned with FDA/EMA frameworks. The resulting lack of harmonized LTFU standards, inconsistent endpoint definitions, observation intervals, and data platforms represents a significant missed opportunity for aggregating safety signals across the global CGT-exposed patient population [3,30,41].

### 4.5. Genotoxicity and Delayed Safety Monitoring of Integrating Vectors

Retroviral and lentiviral vectors achieve durable transgene expression through stable genomic integration, a property that confers therapeutic durability while introducing the risk of insertional mutagenesis via proto-oncogene activation or tumor-suppressor disruption near the integration site [129,130]. Genotoxic events in early gammaretroviral vector trials for X-linked SCID and chronic granulomatous disease resulting in T-cell leukemia in several patients have fundamentally shaped the regulatory science and risk-management paradigm for integrating vectors and continue to define evidentiary expectations for contemporary lentiviral programs [130]. Self-inactivating (SIN) lentiviral vector designs, which delete LTR enhancer elements upon integration, substantially reduce without eliminating enhancer-mediated proto-oncogene activation risk [129]. A comprehensive review of vector-mediated genotoxicity risk factors (integration profile, target cell type, vector dose, patient age, disease context) and current preclinical testing strategies concludes that no universally applicable approach exists; testing must be tailored to the specific vector–cell combination [129].

For AAV products, integration risk is qualitatively different: rAAV DNA exists predominantly episomally, with low-frequency integration lacking retroviral vector site preferences. An integrated scientific/regulatory perspective on AAV integration concludes that while insertional mutagenesis has been reported in neonatal murine models, no confirmed human genotoxic events attributable to therapeutic rAAV have been documented, and theoretical oncogenesis risk must be contextualized against the natural epidemiology of AAV sequences in human tissue [128,131]. ICH S12 addresses integration-site analysis and biodistribution study design, representing the most substantive international consensus on nonclinical safety assessment for integrating and non-integrating vectors [8]. Novel approach methodologies (NAMs) for genome safety assessment, next-generation sequencing-based integration analysis, unbiased whole-genome off-target detection, and in vitro clonogenic assays are expanding across AAV, lentiviral, and nuclease-based modalities [132], but regulatory acceptance criteria for NAM-derived genotoxicity data, and whether they can supplement or replace traditional in vivo studies, remain incompletely defined across the FDA, EMA, and PMDA, a gap that is particularly consequential for CRISPR- and base-editor-based products entering pivotal development, and a tractable priority for ICH-led harmonization.

**Table 3:** Comparative summary of the five core CGT regulatory challenge domains examined in Section 4, cross-referenced to their corresponding pillar within the Global CGT Regulatory Convergence Framework (GCRC-F) proposed in Section 8.

Challenge Domain	FDA	EMA	PMDA	Legal Status	Primary Gap	Pillar
**CMC Complexity & Batch Variability (Section 4.1)**	2020 CMC IND guidance; CQAs defined within validated release specifications per ICH Q8(R2) and ICH Q9(R1) [17,116,133]	Risk-based provisions under Regulation 1394/2007 permit adapted requirements for autologous products [32]	J-GMP, ICH Q10-aligned; no CGT-specific batch-variability guidance issued [43,45]	Final guidance (non-binding) & EU legislation (binding)	No harmonized statistical treatment of acceptable variability thresholds	Pillar 1
**Potency Assay Standardization (Section 4.2)**	2011 potency guidance for cellular- and gene-therapy products; greater early-phase validation flexibility [118,134]	2016 guideline on potency testing of cell-based immunotherapy products (EMA/CHMP/BWP/271475/2006 Rev.1); assessment reports frequently cite validation inadequacy [46,119]	Broadly aligned with EMA expectations; imposes additional qualification specificity for biological raw materials used within potency assay systems under PMDA Notification No. 0709-2 [45]	Final guidance (non-binding) & Scientific guideline (non-binding)	No harmonized validation timing/stringency standard across phases	Pillar 1
**Comparability After Manufacturing Change (Section 4.3)**	Post-approval supplement categories (prior approval supplement, changes being effected in 30 days, annual report); 2023 CGT comparability draft guidance [19]	Type IA/IB/II variation classification under Regulation (EC) No. 1234/2008; comparability expectations set out in the EMA/CAT questions and answers (EMA/CAT/499821/2019) [47,135]	Site-specific validation obligations under the PMD Act; not fully reconciled with ICH Q5E [5,45]	Draft guidance (not operative) & EU legislation (binding)	No CGT-specific harmonized comparability guidance; jurisdiction-tailored packages required	Pillar 1
**Long-Term Follow-Up (Section 4.4 )**	Risk-stratified LTFU of up to 15 years, implemented through REMS and post-marketing commitments [37]	Product-specific Risk Management Plans; no universal time-defined framework [38]	7-year post-CTL confirmatory period; all-case surveillance for approximately 79% of approved products [30,41]	Federal regulation (binding) & Statute (binding)	Inconsistent LTFU endpoint definitions, intervals, data platforms	Pillar 2
**Safety Monitoring of Integrating Vectors (Section 4.5)**	Vigilance anchored in the ICH S12 biodistribution and integration-site framework; risk-based genotoxicity assessment [8,129]	ICH S12 adopted at Step 5 (EMA/CHMP/ICH/318372/2021, effective 30 September 2023); integration-site analysis assessed within the ATMP quality and non-clinical guideline [8,46]	ICH S12 implemented via PMDA notification (October 2023); additional biodistribution expectations under PMDA Notification No. 0709-2 [8,45]	ICH guideline (binding on transposition)	Off-target and oncogenic risk not fully resolvable with currently available clinical follow-up data; no harmonized acceptance criteria for NAM-derived genotoxicity evidence [128,132]	Pillars 1 & 2

Legal status classification: Statute or Regulation denotes binding primary legislation (for example the FD&C Act, Regulation (EC) No. 1394/2007, or the PMD Act); CFR denotes binding US federal regulation; Final guidance denotes non-binding agency guidance representing current thinking, which under 21 CFR 10.115(d) does not establish legally enforceable responsibilities; Draft guidance denotes guidance issued for comment and not yet operative; Reflection paper denotes a non-binding scientific position. ICH guidelines are not directly binding and acquire effect only upon transposition by each regional authority.

## 5. Manufacturing and CMC Bottlenecks in Global CGT Development

Manufacturing complexity is a central regulatory bottleneck determining the feasibility, cost, timeline, and global accessibility of CGT products. Unlike conventional biologics, CGTs require manufacturing systems calibrated to each product class’s biological idiosyncrasies: living cells sensitive to process perturbations, viral vectors whose CQAs are sensitive to scale and platform, donor- and disease-dependent starting materials, and QC panels substituting for the physical uniformity of well-defined small molecules. The four domains below—manufacturing model architecture, viral vector production, raw material/QC variability, and cross-regional GMP inconsistency—define the principal manufacturing–regulatory interface where convergence gaps impose the greatest practical burden.

### 5.1. Centralized Versus Decentralized Manufacturing Architectures

The manufacturing model, centralized at a single GMP facility or distributed across point-of-care (POC)/regional sites, has direct implications for quality consistency, supply-chain oversight, site authorization, and cross-site comparability. Autologous products impose unique logistics: leukapheresis material must be collected, transported under chain-of-identity conditions, processed, and returned within an operationally constrained window while meeting GMP release criteria. The centralized model, currently dominant for commercial CAR-T products (tisagenlecleucel, axicabtagene ciloleucel, lisocabtagene maraleucel), concentrates GMP expertise and oversight but strains supply chains through cryopreservation, international cold-chain logistics, and vein-to-vein timelines exceeding three to four weeks [136].

Decentralized/POC manufacturing addresses these constraints by eliminating long-distance logistics, reducing vein-to-vein time, and enabling fresh-product administration, but introduces commensurate regulatory challenges: multiplied GMP-authorized sites, network-wide harmonization of procedures and quality systems, and cross-site comparability demonstration [137]. A comparative analysis of centralized vs. decentralized CAR-T manufacturing trade-offs concludes that decentralization does not reduce the cost of goods (lost economies of scale) but offers substantive patient-access, supply-chain, and vein-to-vein advantages directly relevant to equitable-access discussions [137]. A tiered QC framework for decentralized manufacturing addressing assay harmonization, rapid-release analytics, and digital QMS integration emphasizes that autologous QC systems require validated rapid-testing platforms assessing identity, potency, sterility, and safety within hours rather than days [138]; neither the principal FDA CMC guidance for gene-therapy investigational applications [20] nor the EU ATMP-specific GMP guidelines [45] establishes a framework for cross-site comparability in multi-site decentralized manufacturing networks, and our review of publicly available guidance from the FDA, EMA, and PMDA current to June 2026 identified no dedicated instrument addressing this manufacturing model.

Within the EU, the hospital exemption (HE) pathway under Article 28 of Regulation 1394/2007 permits ATMP production/use at individual hospital centers without centralized authorization for non-routine, individual-patient preparations [32], enabling institutional CAR-T programs such as ARI-0001 (CART19-BE-01), authorized by Spain’s AEMPS [139]. However, HE implementation varies significantly across Member States, with divergent quality, pharmacovigilance, and reimbursement requirements creating a fragmented landscape [140,141]. A comparative legal analysis across seven Member States documents that such variation creates both patient-safety risks and competitive distortions undermining ATMP framework coherence [141]; the absence of a direct FDA or PMDA equivalent further limits the transferability of European institutional-manufacturing experience.

### 5.2. Viral Vector Production: Scalability, Purification, and Platform Qualification

Viral vectors (rAAV, lentiviral) serve as delivery vehicles for in vivo and ex vivo gene therapies, and their production is among the most technically demanding, capacity-constrained manufacturing domains, with hundreds of active IND/CTA-stage programs competing for finite GMP capacity across internal facilities and CDMOs [142]. For rAAV, dominant upstream platforms are transient triple transfection of HEK293 cells (scalable via bioreactor systems) and baculovirus–insect cell systems (higher yield, distinct capsid glycosylation/PTM profiles) [142,143]. Comprehensive reviews document innovations in perfusion cultures, dual-plasmid transfection, next-generation reagents, and serotype-agnostic affinity/ion-exchange purification, improving capsid enrichment and empty-capsid clearance [142], while critical evaluations identify process variability, low per-batch yields, and insufficiently sensitive process analytical technologies (PATs) as the primary constraints on rAAV scalability and regulatory compliance [143].

Downstream purification to specification, removing empty/partially filled capsids, residual HCP, and host-cell DNA to agency-defined limits, is regulatorily significant. Affinity-resin sanitization protocols enabling multi-cycle reuse without carryover or purity compromise, and affinity-chromatography platforms for adenoviral vector purification, achieving high HCP/DNA clearance with reproducible yields, both demonstrate that downstream process decisions directly determine CQA consistency and regulatory compliance [144,145]. The FDA’s 2020 CMC guidance specifies required characterization parameters (genome titer, capsid ratio, integrity, HCP, residual DNA, sterility, potency) without prescribing specific analytical platforms, with flexibility at the cost of inter-laboratory/inter-jurisdictional standardization [17].

For lentiviral vectors, transient four-plasmid transfection in HEK293T cells remains dominant clinically but is associated with batch variability and high GMP-plasmid costs [146]. A stable producer cell line (SCL) platform eliminating the transfection step demonstrates reproducible production with reduced cost and improved robustness [146], conceptually analogous to stable cell lines in conventional biologics and aligned with the FDA/EMA preference for defined, characterized production substrates [17,46]. However, novel LVV platform qualification (master cell bank characterization, genetic stability, adventitious agent testing) adds development time that may offset near-term savings.

Regulatory expectations are anchored in ICH Q7 and Q10 [42,43], supplemented by CBER [17], EMA/CAT [46], and PMDA [45] product-class guidance, but specificity regarding upstream culture, packaging efficiency, downstream purification, and fill–finish varies substantially. The EMA’s 2021 gene-therapy quality guideline is more prescriptive on impurity characterization than the FDA’s CMC IND guidance, creating divergent dossier architectures for simultaneous IND/CTA programs [46]; the PMDA imposes additional raw-material TSE qualification obligations administered through a distinct Science Board consultation timeline [45].

### 5.3. Raw-Material Qualification and Quality-Control Variability

Raw-material variability is an underappreciated but operationally critical CMC complexity source. Unlike recombinant protein biologics with well-characterized cell banks, CGT products depend on multiple biologically active raw materials, such as viral vectors, plasmid DNA, cytokines, growth factors, serum-derived supplements, and (for autologous products) patient leukapheresis, with each introducing variability requiring qualification and specification systems poorly served by frameworks developed for conventional starting materials [17,42].

Plasmid DNA, used both as a viral vector production input and as a direct drug substance in plasmid-based gene therapies, is a well-characterized variability source with direct regulatory implications: residual bacterial genomic DNA contamination (immunostimulatory CpG motifs) produces Th1-related inflammatory responses in pulmonary gene-transfer models, substantially reduced but not eliminated by CpG-free plasmid sequences [147], establishing the mechanistic basis for regulatory limits on residual genomic DNA, though accepted analytical methods and acceptance criteria differ across the FDA and EMA [17,46].

For autologous cell therapies, the primary raw material, the patient’s own cells, is inherently variable in composition, functional capacity, and viability depending on disease status, prior treatment, and leukapheresis conditions, propagating through every downstream step and constraining tight release specifications without unacceptable out-of-specification rates [136,138]. Agencies have accepted risk-based specification strategies calibrated to biologically achievable quality-attribute ranges rather than absolute conventional-biologic specifications, but specific thresholds and risk-tolerance frameworks differ across the FDA, EMA, and PMDA, requiring jurisdiction-specific release-specification matrices for global programs.

### 5.4. GMP Divergence and the Absence of CGT Mutual Recognition

GMP regulations exist across all major jurisdictions but are neither uniformly codified nor mutually recognized, such that authorization from one agency evidences compliance for another, imposing redundant inspection, qualification, and documentation burden on multi-regional developers. The most comprehensive comparative GMP gap analysis, across the US, the EU, Japan, and South Korea, finds shared core principles (sterility assurance, contamination prevention, batch records, equipment qualification) but substantive differences in: (1) dossier structure (site master file mandatory in the EU/Japan/South Korea but not the US, where the BLA incorporates facility information); (2) risk-based inspection approaches (more formal in the EU/Japan than the US); and (3) personnel qualification standards (the PMDA’s qualified-person concept reflecting EU origin within a distinct national framework) [148].

In the US, CGT manufacturing is governed by 21 CFR Part 1271 [15] supplemented by cGMP under 21 CFR Parts 210/211 and CBER gene-therapy CMC guidance [17]. In the EU, ATMP-specific GMP guidelines adapt the general EudraLex Volume 4 framework to ATMP manufacturing challenges [32,44]. The PMDA implements GMP oversight through the PMD Act/ASRM framework with supplementary notifications [45]. These three frameworks are not mutually recognized: an FDA GMP inspection does not satisfy EU requirements and vice versa, requiring multi-regional sites to manage simultaneous or sequential independent national inspections.

Emerging-market agencies (NMPA, ANVISA, CDSCO, MFDS) are developing GMP frameworks variably aligned with ICH Q7 and Q10 [42,43]. A comparative analysis of safety, toxicity, and GMP heterogeneity across the US, the EU, India, China, and Australia identifies the absence of unified global approaches for complex, dynamically transforming biological materials as a major shared gap [149], while divergent national implementation of hospital-exemption and compassionate-use routes creates parallel pathways undermining GMP coherence for institutional ATMP manufacturing [150].

No structural GMP mutual-recognition solution analogous to conventional pharmaceutical GMP mutual-recognition agreements exists for CGT products. ICH Q10 provides a technically applicable, broadly endorsed quality-system framework [43], but implementation at the inspection-protocol and site-authorization level remains jurisdiction-specific. A CGT-specific GMP mutual-recognition or reliance framework, potentially via the ICMRA or bilateral FDA/EMA/PMDA collaboration, represents a tractable harmonization priority that would directly reduce operational burden on multi-regional programs and lower access barriers in markets lacking independent complex-biologics GMP inspection capacity.

## 6. Clinical Development and Evidentiary Requirements

CGT clinical evidence generation confronts a structural tension absent in conventional pharmaceuticals: target diseases are rare/ultra-rare, and treatment effects are often large and mechanistically plausible, yet the marketing-authorization standard was historically built around adequately powered randomized designs for common conditions. This section examines four interrelated challenges: small-population trial design, single-arm versus randomized designs, real-world evidence (RWE) integration, and pediatric pathways, each reflecting a distinct facet of the same underlying problem of generating rigorous, achievable evidence within epidemiological constraints. Table 4 summarizes these four challenges together with the regulatory anchor applicable to each.

### 6.1. Trial Design Under Small-Population Constraints

Most approved and investigational CGT products target diseases with global prevalence measured in the hundreds to low thousands of patients, and in some cases, fewer than one hundred. This epidemiological reality directly constrains the feasibility of conventional trial designs: adequately powered two-arm superiority trials with pre-specified alpha and beta error rates may require sample sizes that simply do not exist within the treatable population, particularly when a proportion of that population is excluded by trial eligibility criteria, geographic accessibility, or competing enrollment in other investigational programs. Orfali et al. [151] provide empirical confirmation of this constraint, demonstrating in a comparative analysis of FDA-approved orphan and non-orphan drugs (2001–2011) that orphan drug approvals were supported by significantly fewer clinical trials and substantially smaller total enrolled populations (mean 390 vs. 2566 participants) than non-orphan approvals, while proportions of randomized, blinded, and placebo-controlled designs remained statistically similar between the two groups, indicating that regulators have historically accepted smaller, but not categorically less rigorous, evidence packages for rare-disease indications.

The EMA’s regulatory guidance directly addressing Clinical Trials in Small Populations (CHMP/EWP/83561/2005) [152] establishes that no methodological approach is exclusive to small populations, but acknowledges that less conventional statistical and design strategies may be justified where they improve the interpretability of results obtained from an inherently limited patient pool. The FDA’s guidance on rare diseases [153] similarly states that the agency does not apply a different statutory standard of evidence for orphan indications, while explicitly encouraging innovative trial design, natural history study integration, and flexible statistical approaches to meet that standard within small-population constraints.

Several methodological innovations have been developed specifically to address small-population constraints. Chow and Huang [154] propose a two-stage adaptive seamless trial design integrated with real-world data principles, alongside a “demonstrating not-ineffectiveness” framework as an alternative to conventional effectiveness demonstration when subject availability is severely constrained. The EU-funded ASTERIX project, evaluated retrospectively by Mitroiu et al. [155] against 26 European Public Assessment Reports for orphan medicinal products, found that methods including extrapolation frameworks, sample size re-estimation, Bayesian dynamic borrowing through power priors, and optimal sequential designs for small samples were applicable in over 40% of evaluated cases when design flexibility within the broader development program was permitted, though direct applicability using the originally planned trial design was considerably more limited. Harmatz et al. [156] demonstrate a practical implementation of one such innovation, a Blind Start crossover design combined with a Multi-Domain Responder Index, in a 12-subject trial of enzyme replacement therapy for an ultra-rare lysosomal disease, illustrating how composite responder endpoints and within-subject crossover comparisons can enhance statistical power in settings where conventional parallel-group randomization is not feasible. While developed for an enzyme replacement product, this design logic is directly transferable to CGT trials facing comparable population constraints.

### 6.2. Single-Arm Versus Randomized Designs and External Controls

Most pivotal CGT trials to date (tisagenlecleucel, axicabtagene ciloleucel, voretigene neparvovec in initial indications) have relied wholly or substantially on single-arm designs assessed against historical/literature benchmarks, reflecting disease severity/progression (ethical objections to withholding plausibly transformative therapy), small population constraints, and the one-time administration nature of most CGTs precluding crossover designs. The most comprehensive empirical analysis, examining all 92 novel FDA cancer-drug approvals (2000–2016) across 127 pivotal trials, found only 51.2% randomized and 74.8% open-label, with a median absolute overall-survival benefit of only 2.40 months, establishing the broader oncology-approval context within which single-arm CGT approvals occur, with correspondingly limited comparative-effectiveness data available at approval [157].

Where feasible, innovative randomized designs preserve statistical efficiency: optimal predictive-probability designs for randomized biomarker-guided oncology trials incorporating futility monitoring achieved average sample sizes 23–48% smaller than an originally realized single-arm trial while still generating comparative data [158], showing that the single-arm/randomized choice is not a strict feasibility binary, and that adaptive/Bayesian designs represent a genuine regulatory-science opportunity where randomization is ethically defensible. ICH E10 governs the conditions under which externally controlled trials using historical/natural-history comparators can provide adequate effectiveness evidence, emphasizing well-characterized disease course, large treatment effects, and closely matched comparators [159]. The FDA and EMA have both accepted single-arm pivotal evidence meeting the ICH E10 conditions [159], but our review identified no published CGT-specific quantitative threshold from either agency defining when externally controlled evidence is sufficient, leaving such determinations to case-by-case scientific advice, which is a continued source of uncertainty for global pivotal-trial strategy.

### 6.3. Real-World Evidence Integration Across the CGT Lifecycle

Real-world evidence (RWE) from registries, EHRs, claims, and other non-trial sources has become central across the CGT evidence lifecycle: pre-approval natural-history characterization, post-approval confirmatory evidence, long-term safety surveillance, and cross-product comparative effectiveness. The US regulatory foundation derives from the 21st Century Cures Act (2016) and the FDA’s 2018 RWE Framework, operationalized through 2024 final guidance on EHR/claims-derived real-world data [48]; the EMA’s parallel 2021 guideline on registry-based studies is directly relevant given the centrality of registries (CIBMTR, EBMT) to hematopoietic cell-therapy LTFU [49]. Data reliability is a foundational challenge: the FDA-funded TRUST demonstration project across 58 US hospitals found that combining structured claims data with AI-extracted unstructured EHR data achieved substantially higher accuracy (93.4% vs. 59.5% F1), completeness (96.6% vs. 46.1%), and source traceability (77.3% vs. 11.5%) than claims-only approaches [160], directly relevant given the multi-domain, longitudinal nature of CGT LTFU data requiring comparably sophisticated RWE infrastructure.

For CAR-T therapies, RWE has already demonstrated substantial value: an inverse-probability-weighting analysis comparing lisocabtagene maraleucel and axicabtagene ciloleucel in 160 real-world large B-cell lymphoma patients over three years found comparable survival despite differential toxicity, a comparison never conducted, and unlikely ever to be conducted, as an RCT given both products’ availability [161]. The German GLA/DRST registry analysis (356 patients, 21 centers) provided real-world risk-factor identification (bridging-therapy success, LDH level, product selection) directly informing risk-adapted treatment strategies unattainable within the original pivotal trials [162]. A real-world longitudinal cohort further extended RWE’s evidentiary role to patient-reported and neurocognitive outcomes, finding quality-of-life improvements after axicabtagene ciloleucel generally consistent with or exceeding pivotal-trial results [163]. Despite this expanding evidence base, no FDA, EMA, or PMDA guidance specifies harmonized minimum data standards, registry interoperability requirements, or statistical methodology expectations for CGT RWE submissions, a direct extension of the Section 4.4 LTFU harmonization gap, underscoring that RWE standardization spans pre-approval contextualization, post-approval label expansion, and cross-product comparative research.

### 6.4. Pediatric CGT Development: Regulation, Extrapolation, and Access

Pediatric CGT development presents a distinctive dynamic: unlike most drug classes following adult-to-pediatric extrapolation, several major CGTs, notably CD19-directed CAR-T for B-cell precursor ALL, were developed and approved first, or nearly simultaneously, in pediatric/young-adult populations, reflecting target-disease epidemiology. This pattern benefited from a well-characterized target antigen (CD19), a tractable pediatric leukemia population, and strong pediatric oncology trial-network infrastructure not uniformly present across other modalities [164]. The FDA’s RMAT guidance imposes no CGT-specific pediatric requirements beyond the standard Pediatric Research Equity Act (PREA) [33], while the EU Pediatric Regulation (EC) No. 1901/2006 mandates a Pediatric Investigation Plan (PIP) for most new medicinal products, including ATMPs, absent a waiver [165]. ICH E11(R1) provides the international extrapolation framework, but its CGT applicability is complicated by the frequent absence of an adult indication to extrapolate from, and by fundamental pediatric–adult differences in immune ontogeny, hematopoietic reserve, and vector pharmacokinetics limiting simple dose-scaling [166]. Multi-stakeholder initiatives address these gaps directly: the seventh Pediatric Strategy Forum (ACCELERATE, with EMA and FDA participation) comprehensively reviewed pediatric CAR-T development, identifying priorities including early regulatory engagement, alignment of scientific/HTA requirements from trial inception, and standardized frameworks for evaluating alternative targets in very small pediatric populations, directly reflecting the small-population and single-arm challenges of Section 6.1 and Section 6.2, compounded by even smaller pediatric populations and heightened control-arm ethical sensitivity [167].

A further pediatric-specific challenge concerns sustaining access to academically developed genetically modified cell therapies (GMCTs) and the lack of commercial sponsorship for full-registration trials, which disproportionately affects pediatric/ultra-rare populations with limited commercial ROI. The ASTCT “ACT To Sustain” task force documents the “valley of death” facing academic GMCT products post-proof of concept and proposes aligning FDA designations (e.g., RMAT) with payer coverage determinations and exploring alternative access pathways, a structural tension that existing frameworks (RMAT, PRIME, Sakigake) do not fully resolve [168].

**Table 4:** Summary of the four principal clinical-evidence generation challenges for CGT development discussed in Section 6, with the corresponding regulatory anchor in each case.

Evidentiary Challenge	Core Constraint	Regulatory Anchor
**Small-population trial design (Section 6.1)**	Orphan CGT indications too small for conventionally powered two-arm trials [151]	EMA CHMP/EWP/83561/2005; FDA rare-disease guidance [152,153]
**Single-arm vs. randomized trials (Section 6.2)**	Ethical and feasibility barriers to withholding a plausibly transformative therapy from a control arm [157]	ICH E10 (choice of control group; externally controlled trials) [159]
**Real-world evidence integration (Section 6.3)**	No harmonized minimum data standards, registry interoperability requirements, or statistical methodology expectations for CGT real-world evidence submissions across the FDA, EMA, and PMDA; compounded by demonstrated variability in the accuracy, completeness, and traceability of real-world data sources [42,160]	21st Century Cures Act; FDA 2018 RWE Framework and 2024 guidance; EMA guideline on registry-based studies (EMA/426390/2021) [48,49]
**Pediatric approval pathways (Section 6.4)**	Pediatrics-first development for several CGTs, reversing the conventional adult-to-pediatrics translation sequence [164]	Pediatric Research Equity Act (PREA) and FDA-expedited program guidance for regenerative medicine therapies; EU Pediatric Regulation (EC) No. 1901/2006 requiring a Pediatric Investigation Plan for most ATMPs; ICH E11(R1) extrapolation framework [36,166,167]

## 7. Global CGT Regulatory Convergence Efforts: Progress and Limitations

The preceding sections document substantial divergence across CMC expectations, accelerated access, and clinical evidence standards between the FDA, EMA, PMDA, and BRIC-M agencies. This section examines the institutional mechanisms pursuing convergence: ICH technical guideline development, multilateral collaboration through ICMRA and regional work-sharing consortia, bilateral FDA-EMA parallel scientific advice, and the structural limitations constraining these instruments’ pace and depth.

### 7.1. ICH Guidelines: Achievements, Membership Constraints, and Implementation Gaps

The ICH remains the principal harmonization vehicle underpinning virtually all CMC, nonclinical, and clinical expectations discussed elsewhere. ICH S12, reaching Step 5 in 2023, is the single most consequential CGT harmonization achievement, providing aligned biodistribution/shedding/germline-integration-risk expectations; its implementation across the FDA, the EMA, the PMDA, and Health Canada has materially reduced jurisdiction-specific nonclinical biodistribution packaging requirements [8]. ICH Q5E, though pre-dating CGTs as a distinct class, remains the default comparability framework applied by all three agencies (Section 4.3), but its biochemical/physicochemical paradigm imperfectly accommodates cell-therapy phenotypic/functional comparability; the ICH’s ongoing Q5 revision concept paper reflects a preliminary effort to extend coverage to advanced-therapy characterization, illustrating the ICH’s deliberately incremental, consensus-prioritizing pace [5,169].

The ICH’s membership architecture is tiered rather than restrictive, and the constraint it imposes is subtler than exclusion. Founding Regulatory Members, the European Commission, the US FDA, and Japan’s MHLW/PMDA hold permanent Management Committee seats, as do the Standing Regulatory Members, Health Canada and Swissmedic. Ten further agencies hold elected Regulatory Member status, among them Brazil’s ANVISA, China’s NMPA, and Mexico’s COFEPRIS, all of which are formally expected to implement all ICH guidelines; India (CDSCO) and Russia (Roszdravnadzor) participate only as Observers. The operative constraint for CGT convergence is therefore not formal exclusion from the ICH but asymmetry in implementation capacity and in influence over guideline drafting: three of the five BRIC-M agencies carry full implementation obligations without proportionate representation in the technical working groups that set the standards they are obliged to adopt [73]. Even between the two most ICH-aligned major jurisdictions (EU/US), guideline implementation timing and interpretive emphasis differ meaningfully [123], a gap widening considerably for more recently or partially engaged BRIC-M agencies; EU ATMP guideline adoption is further filtered through EMA/CAT-specific implementation guidance, introducing interpretive divergence even where the underlying ICH text is formally adopted [170], underscoring that ICH-level harmonization does not automatically translate to implementation-level harmonization.

### 7.2. Work-Sharing, Reliance, and Multilateral Coordination Mechanisms

Beyond the ICH’s guideline function, a broader ecosystem addresses harmonization needs that guideline development alone cannot resolve. The Access Consortium (Australia’s TGA, Health Canada, Singapore’s HSA, Swissmedic, the UK’s MHRA) operates the New Active Substance Work-Sharing Initiative (NASWSI), through which member agencies jointly review new active substance applications, including advanced therapies. An industry survey across all five jurisdictions found predominantly positive sponsor experience but identified persistent procedural unpredictability, transparency gaps, and multi-agency interface complexity requiring sustained harmonized-guidance investment rather than one-time reform [171].

For BRIC-M and other constrained-capacity agencies, reliance and work-sharing substitute mutual recognition of an originating agency’s assessment for full independent review. A survey of 21 African National Regulatory Authorities found risk-based work-sharing and unilateral reliance to be the most commonly adopted mechanisms, citing lack of formal reliance guidelines and limited access to originating-agency assessment reports as primary barriers [172]; though addressing African rather than BRIC-M regulators, these constraints parallel those documented for ANVISA (Section 2.4), suggesting similar barriers across resource-constrained systems generally. ICMRA provides further collaborative infrastructure convening ICH and non-ICH regulators around shared priorities, including CGT oversight innovation [173]. Proceedings from a 2021 Global Annual Meeting document ICMRA’s pandemic-era coordination role and highlight parallel scientific advice and global collaboration as recurring cross-stakeholder priorities [174].

### 7.3. Bilateral Alignment: FDA-EMA Parallel Scientific Advice and Designation

The FDA-EMA Parallel Scientific Advice (PSA) program enables simultaneous, coordinated bilateral input within a single consultative process rather than sequential independent interactions [175], offering CGT developers a practical avenue to reconcile diverging expectations (e.g., potency-assay validation timing, natural-history comparator selection, LTFU design) before they manifest as costly late-stage amendments.

A systematic comparison of FDA and EMA outcomes for 115 new active substances approved by either/both agencies (2014–2016) found general conditional-approval concordance (8 of 52 simultaneously submitted substances by both agencies) but notable divergence in expedited-pathway use (13 both vs. 18 FDA-only) and orphan designation (17 both vs. 10 FDA-only), plus a timeline gap (median 319 days FDA vs. 409 EMA) [176], demonstrating that even simultaneous submission and PSA engagement does not eliminate substantive divergence between the two most closely aligned major systems. Examining FDA Breakthrough Therapy and EMA PRIME applications specifically, developers frequently pursue both, but designation outcomes, timing, and feedback substance are not systematically aligned, requiring sponsors to manage two parallel, only partially convergent relationships [177]. Comparing BTD, PRIME, and Sakigake directly, no product had, at analysis time, received all three designations concurrently, and the systems continue operating largely independently despite shared objectives and ICH/ICMRA interconnection [36].

### 7.4. Why Current Harmonization Instruments Fall Short

Current harmonization mechanisms remain insufficient to resolve the CMC, pathway, and evidentiary divergences structurally defining global CGT regulation. First, harmonization operates asymmetrically: ICH guideline development, Access Consortium work-sharing, and FDA-EMA PSA concentrate convergence effort among the most ICH-aligned agencies, while BRIC-M agencies participate asymmetrically: India and Russia only as Observers, and Brazil, China, and Mexico as Regulatory Members carrying full guideline-implementation obligations but exercising limited influence over the technical working groups in which those guidelines are drafted. This asymmetry is consequential given the scale of China’s and Brazil’s CGT markets and China’s manufacturing significance (Section 3.1, Section 3.2 and Section 3.6).

Second, formal alignment does not reliably produce concordant outcomes even among closely coordinating agencies: a retrospective analysis of 814 FDA orphan drug approvals (2011–2023) found that only 29% received corresponding EMA authorization with orphan designation, 38% EMA authorization without orphan status, and 33% no EMA authorization at all, with discordance widening (not narrowing) in 2017–2023 versus 2011–2016 (OR 0.66 for EMA authorization), disproportionately affecting non-cancer and small/medium-sponsor approvals [178], Though not CGT-specific, given substantial CGT orphan-designation overlap, widening discordance over a period of significant ICH S12/PSA maturation strikingly indicates existing instruments have not kept pace with the divergence they address.

Third, harmonization instruments concentrate on procedural/designation-level alignment (parallel advice scheduling, simultaneous designation applications, work-sharing logistics) rather than the substantive technical/evidentiary standards of potency bridging, platform CMC recognition, LTFU data standardization, and single-arm/randomized thresholds (Section 4, Section 5 and Section 6) that primarily drive duplicative burden. Even within a single ostensibly harmonized area (EU ATMP regulation), national implementation variability persists at the member-state level [179], as reflected in ATMP clinical trial regulation in Germany within the broader EU framework established by Regulation (EC) No. 1394/2007 [32], illustrating that formal supranational instruments do not eliminate implementation-level divergence even within their own jurisdictional scope.

Taken together, ICH guidelines, work-sharing consortia, ICMRA coordination, and bilateral PSA have achieved meaningful but partial, unevenly distributed progress; ICH S12 shows that guideline-level consensus is achievable among closely aligned agencies within several years, but translating that into concordant outcomes, substantive (not observer-level) BRIC-M participation, and technical rather than procedural harmonization will require more structurally ambitious mechanisms.

## 8. The Global CGT Regulatory Convergence Framework (GCRC-F): A Modular, Maturity-Tiered Architecture

### 8.1. Rationale, Design Principles, and Relationship to Existing Proposals

**Figure 4:**
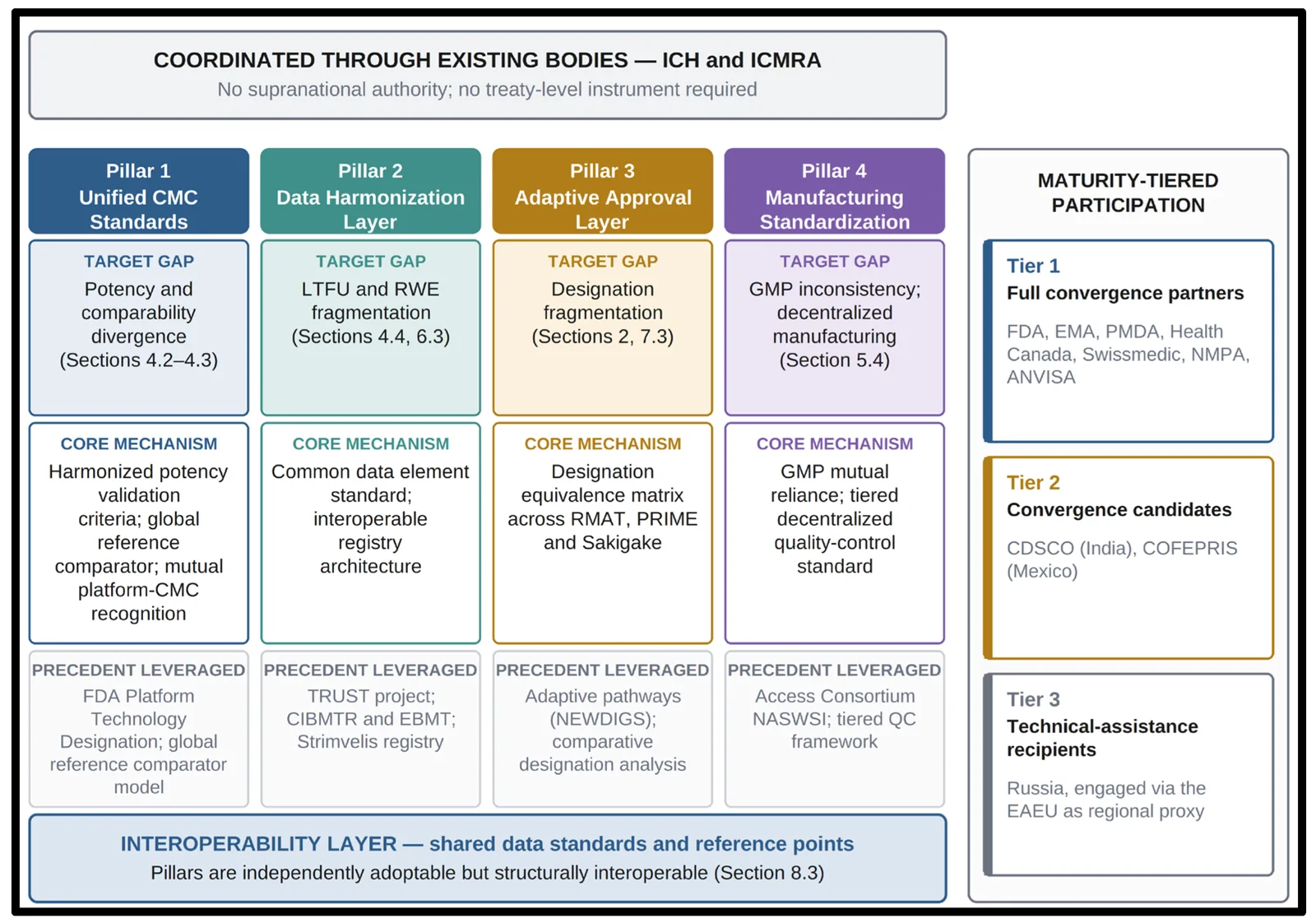
Architecture of the proposed Global CGT Regulatory Convergence Framework (GCRC-F). The framework comprises four independently adoptable pillars, each addressing a specific divergence domain identified in Section 4, Section 5, Section 6 and Section 7 and each built upon an existing regulatory precedent rather than a novel instrument. Coordination is proposed through existing international bodies (ICH and ICMRA); the framework does not require a supranational authority or treaty-level agreement. The shared data standards described in Section 8.3 provide interoperability between pillars for agencies electing to adopt more than one. Participation is tiered by demonstrated regulatory-science maturity rather than extended uniformly, as set out in the right-hand panel and discussed in Section 8.3 and Section 11.2. CMC, Chemistry, Manufacturing, and Controls; EBMT, European Society for Blood and Marrow Transplantation; GMP, good manufacturing practice; ICMRA, International Coalition of Medicines Regulatory Authorities; LTFU, long-term follow-up; NASWSI, New Active Substance Work-Sharing Initiative; QC, quality control; RWE, real-world evidence.

Section 4, Section 5, Section 6 and Section 7 of this review have documented a consistent pattern: the harmonization mechanisms presently available to cell- and gene-therapy (CGT) regulation. International Council for Harmonization (ICH) guidelines, multilateral work-sharing consortia, and bilateral parallel scientific advice have achieved meaningful but partial and unevenly distributed convergence, concentrated predominantly at the procedural and designation level rather than at the level of the substantive technical and evidentiary standards that most directly drive duplicative development burden. Based on this evidence, we propose a Global CGT Regulatory Convergence Framework (GCRC-F) comprising four interdependent pillars: Unified CMC Standards, a Data Harmonization Layer, an Adaptive Approval Layer, and a Manufacturing Standardization Layer. The GCRC-F is not proposed as a supranational regulatory authority, a model with no realistic near-term political feasibility given the sovereign basis of pharmaceutical regulation in every jurisdiction reviewed, but rather as a modular technical reference architecture that individual agencies, coordinating through existing bodies, including the ICH and the International Coalition of Medicines Regulatory Authorities (ICMRA), could adopt incrementally and non-exclusively (Figure 4).

The GCRC-F is not the first proposal to address regulatory fragmentation in cell and gene therapy, and the framework advanced here is best understood in relation to that existing body of work. Hashmi et al. have recently reviewed the leading jurisdictional frameworks and proposed a harmonized global blueprint built upon adaptive trial design, contemporary endpoints, and modernized manufacturing approaches [180], while a multi-stakeholder workshop convened in September 2024 by the Reagan-Udall Foundation for the FDA, in collaboration with the FDA and the Gates Foundation, set out convergence priorities for gene therapies, with sickle-cell disease as the model disorder and equitable access in low- and middle-income countries as the organizing concern [181]. Regional analyses have mapped convergence pathways across six Asian markets, including the role of the ASEAN Pharmaceutical Product Working Group and the APEC Regulatory Harmonization Steering Committee [182], and comparative work has situated the Brazilian advanced-therapy framework against those of the United States, the European Union, and Japan [183]. Earlier, the Global Gene Therapy Initiative established that regulatory infrastructure development and capacity building, rather than technical standards alone, condition whether harmonization translates into access [184]. What distinguishes the framework proposed here is therefore neither the objective of convergence nor the modular design principle, both of which have precedent, but its scope and its treatment of emerging markets: to our knowledge, this is the first proposal to integrate the full BRIC-M set into a single technical architecture, and to tier participation according to demonstrated regulatory-science maturity rather than treating emerging markets as a homogeneous class of reliance recipients.

The rationale for a four-pillar structure, rather than a single omnibus harmonization instrument, follows directly from the gap analysis presented earlier in this review. CMC divergence (Section 4), evidentiary standard divergence (Section 6), accelerated pathway divergence (Section 2 and Section 7.3), and manufacturing/GMP divergence (Section 5.4) each involve distinct technical communities, distinct regulatory instruments, and, in several cases, distinct legal bases within each agency’s governing statute. A single unified instrument attempting to harmonize all four simultaneously would face the same consensus-building constraints that have limited the pace of the ICH’s own guideline development [5,8]. A modular architecture, by contrast, allows each pillar to progress at a pace commensurate with its technical readiness and regulatory consensus, while remaining structurally interoperable with the others through shared data standards and reference points described in Section 8.3. Table 5 situates the proposed framework relative to the international convergence initiatives already in operation, identifying for each what it covers, whether it is binding, and whether it extends to cell and gene therapy and to emerging-market regulators.

A precedent for the modular, incremental harmonization logic underlying the GCRC-F is provided by Kirchlechner and Cohen [185], whose descriptive review of biosimilar development harmonization hurdles, including locally sourced reference product requirements, redundant comparative toxicology studies, divergent clinical study design expectations, and ethnic sensitivity data requirements, proposes targeted, hurdle-specific recommendations rather than a single unified biosimilar approval standard. While biosimilars and CGTs are regulated under distinct statutory frameworks in most jurisdictions, the biosimilar harmonization experience is instructive: incremental, technically specific harmonization proposals addressed to identified hurdles have demonstrated more tractable uptake among regulatory agencies than calls for wholesale regulatory unification, and the GCRC-F’s pillar structure is designed to follow this same evidentiary logic.

### 8.2. Pillar 1: Unified CMC Standards

Pillar 1 addresses divergence in potency assessment and comparability. It does not propose unified potency assays. Potency in cell and gene therapy is irreducibly product-specific: the relevant biological activity of an autologous CAR-T product, a gene-edited hematopoietic stem-cell product and an adeno-associated virus vector are not measurable by any common method, and the FDA has stated that “due to the diversity of CGT products and the product-specific nature of potency assays,” its own recommendations on assay selection and design are “necessarily general” [134].

What can be harmonized is the framework within which product-specific assays are developed, qualified and validated. Three elements are proposed. First, common validation principles anchored in ICH Q2(R2) and ICH Q6B, specifying what constitutes adequate demonstration of specificity, accuracy, precision and linkage to the mechanism of action, applied to whatever assay a given product requires. Second, a shared expectation as to when a matrix of complementary assays is required rather than a single measure, and how the elements of such a matrix should relate to identified critical quality attributes. Third, alignment on the risk-based potency assurance strategy that the FDA’s 2023 draft guidance introduces, so that a sponsor constructing such a strategy is not required to reconstruct it differently for each jurisdiction.

On reference materials, the proposal is for collaborative international standard development rather than for a single global comparator. Progress here is real but partial. A WHO reference reagent for lentiviral vector copy number determination by digital PCR has been established [186], and adeno-associated virus reference materials have been developed through a multi-center collaborative study. No established international reference standard for CAR-T products yet exists. Extending this model product class by product class, through the existing WHO and national standards infrastructure, is more tractable than any attempt at a universal comparator.

Four product classes resist harmonization to differing degrees, and the framework does not claim otherwise. Autologous products are manufactured individually, and their process is inseparable from the product, limiting what comparability data can demonstrate. Gene-edited products raise off-target and integration-site questions that ICH S12 does not address. Viral vectors differ in characterization requirements by serotype and production platform. Decentralized and point-of-care manufacture remains without a settled international framework: the European Union addresses multi-site ATMP manufacture through EudraLex Volume 4 Part IV [187], the United Kingdom introduced the first dedicated point-of-care and modular manufacture framework in July 2025 [110], and the FDA has published a discussion paper but no final guidance [188]. Pillar 1 is accordingly scoped to validation principles and reference-standard collaboration, which are tractable, rather than to assay-level harmonization, which is not.

### 8.3. Pillar 2: Data Harmonization Layer

Pillar 2 addresses the long-term follow-up (LTFU) and real-world evidence (RWE) standardization gaps documented in Section 4.4 and Section 6.3 by proposing a common data element standard and interoperable registry architecture specific to CGT long-term follow-up. Nagel and Roman’s narrative review of RWE’s expanding regulatory role [189] documents concrete precedents for RWE serving as a primary or supplementary evidentiary foundation in FDA regulatory decisions, including tumor-agnostic approval extensions and rare-disease indication expansions supported by patient registry data, establishing that the regulatory acceptability of well-governed RWE is no longer the primary barrier to its expanded use; rather, the data quality and interoperability infrastructure remain the binding constraints.

The data-reliability infrastructure demonstrated by Riskin et al.’s FDA-funded TRUST demonstration project [160], which achieved substantially higher accuracy, completeness, and source traceability through combined structured-and-unstructured data curation relative to claims-only approaches, provides a template for the technical specifications the CDE standard should incorporate: multi-domain longitudinal data capture (oncologic, immunologic, quality of life), source traceability sufficient for regulatory audit, and interoperability across the registry infrastructure already in productive use for CGT LTFU, including CIBMTR, EBMT, and product-specific registries modeled on the Strimvelis precedent described by Stirnadel-Farrant et al. [126]. A CDE standard adopted jointly by the FDA and EMA, with reference to their respective RWE and registry-based study guidance [48,49], would allow a single LTFU protocol and registry infrastructure to satisfy both agencies’ post-approval evidence requirements, directly resolving the protocol duplication burden identified in Section 4.4.

Extending this data layer to BRIC-M jurisdictions presents a distinct implementation challenge: unlike the FDA and EMA, most BRIC-M agencies have not yet published CGT-specific RWE or registry guidance, meaning the CDE standard must be designed for forward compatibility with agencies whose regulatory science capacity is still developing, rather than assuming symmetric technical infrastructure across all participating jurisdictions from the outset. A phased data layer in which BRIC-M agencies initially participate as registry data recipients under a reliance-style arrangement before progressing toward full CDE contribution offers a more realistic implementation path than requiring simultaneous full participation.

### 8.4. Pillar 3: Adaptive Approval Layer

Pillar 3 addresses the accelerated pathway fragmentation documented in Section 2 and Section 7.3, the independent, only partially convergent operation of the FDA’s RMAT and Breakthrough Therapy Designation; the EMA’s PRIME and Conditional Marketing Authorization; and the PMDA’s Sakigake and Conditional and Time-Limited Approval (CTLA) systems. The conceptual foundation for this pillar is the adaptive pathways paradigm articulated by Eichler et al. [190] in a multi-stakeholder analysis developed under the NEWDIGS initiative of the MIT Center for Biomedical Innovation, in which regulators, including the EMA, participated alongside industry, payer, and patient stakeholders. The concept was subsequently tested by the EMA through its own adaptive pathways pilot between 2014 and 2016, a trajectory from multi-stakeholder technical proposal to regulatory pilot that the present framework deliberately seeks to replicate, which proposes a life-span approach to drug development: iterative, staged evidence generation and regulatory engagement spanning from early conditional access through confirmatory real-world data collection, replacing the single-point-in-time, all-or-nothing licensing decision that has historically characterized marketing authorization. This life-span logic maps directly onto the existing but disconnected accelerated pathway architecture already in place across the FDA, EMA, and PMDA. The adaptive approval layer’s function is not to create a new regulatory authority but to formally interconnect designation, evidence generation, and confirmatory review stages that currently operate as parallel, independently negotiated tracks in each jurisdiction.

Concretely, the adaptive approval layer proposes a harmonized designation-equivalence matrix building on the direct comparative analysis of RMAT, PRIME, and Sakigake conducted by Kondo et al. [36], under which a developer receiving RMAT designation from the FDA would be entitled to an expedited, reduced-burden PRIME or Sakigake eligibility assessment from the EMA or PMDA, respectively, rather than undergoing an independent designation determination from first principles. Hanaizi et al.’s empirical analysis of parallel BTD/PRIME applications [177] demonstrates that developers already pursue simultaneous designation-seeking as a practical strategy; formalizing designation-equivalence recognition would convert this ad hoc developer practice into a structured, agency-endorsed pathway, reducing the duplicative regulatory engagement burden documented in Section 7.3.

The confirmatory evidence component of the adaptive approval layer directly interfaces with Pillar 2: post-conditional-approval confirmatory obligations, whether structured as FDA-accelerated approval confirmatory trials, EMA CMA renewal obligations, or the PMDA’s post-CTLA commercial-phase evidence generation [33,35,191], would draw on the common CDE registry infrastructure proposed under Pillar 2, allowing a single confirmatory evidence package to satisfy the distinct but overlapping post-approval obligations imposed by each agency, rather than requiring independently designed confirmatory study protocols for each jurisdiction. Table 6 sets out the legal basis, eligibility criteria, evidentiary threshold, regulatory benefits and post-authorization obligations of each mechanism, and distinguishes the three designations from the two approval pathways with which they are frequently conflated.

### 8.5. Pillar 4: Manufacturing Standardization Layer

Pillar 4 addresses the GMP inconsistency and manufacturing model fragmentation documented in Section 5 through a two-component structure: a GMP mutual reliance mechanism and a decentralized manufacturing-quality framework. The GMP mutual reliance mechanism builds directly on the comparative gap analysis of Shin and Kim [148], who demonstrated that GMP dossier requirements, facility inspection approaches, and personnel qualification standards differ substantively in structure across the FDA, EMA, PMDA, and South Korea’s MFDS despite sharing common ICH Q7 and Q10 [43] quality system foundations. Rather than proposing full mutual recognition, which would require legislative change in most jurisdictions, the GCRC-F proposes a reliance-based model in which a GMP inspection conducted by any ICH Founding or Standing Regulatory Member is accepted as primary evidence by a second such agency, subject to a reduced-scope confirmatory desk review rather than an independent full inspection, analogous in structure to the Access Consortium’s New Active Substance Work-Sharing Initiative model described in Section 7.2.

The decentralized manufacturing-quality framework component responds directly to the point-of-care and hospital exemption manufacturing challenges documented in Section 5.1. McCann et al.’s economic and operational analysis of centralized versus decentralized CAR-T manufacturing [137] establishes that decentralization offers genuine access and vein-to-vein time advantages that a harmonization framework should preserve rather than regulate out of existence through excessive standardization burden; accordingly, Pillar 4 proposes adoption of a tiered quality-control standard modeled on the framework developed by Nahum et al. [138], under which distributed manufacturing sites operating within a single sponsor’s network are held to a harmonized rapid-release analytical standard rather than site-specific, independently negotiated release criteria, directly enabling the cross-site product comparability that Section 5.1 identified as the principal quality challenge of decentralized manufacturing models. Table 7 summarizes the four pillars, the divergence gap each address, its core mechanism, and the existing regulatory precedent on which it builds.

**Figure 5:**
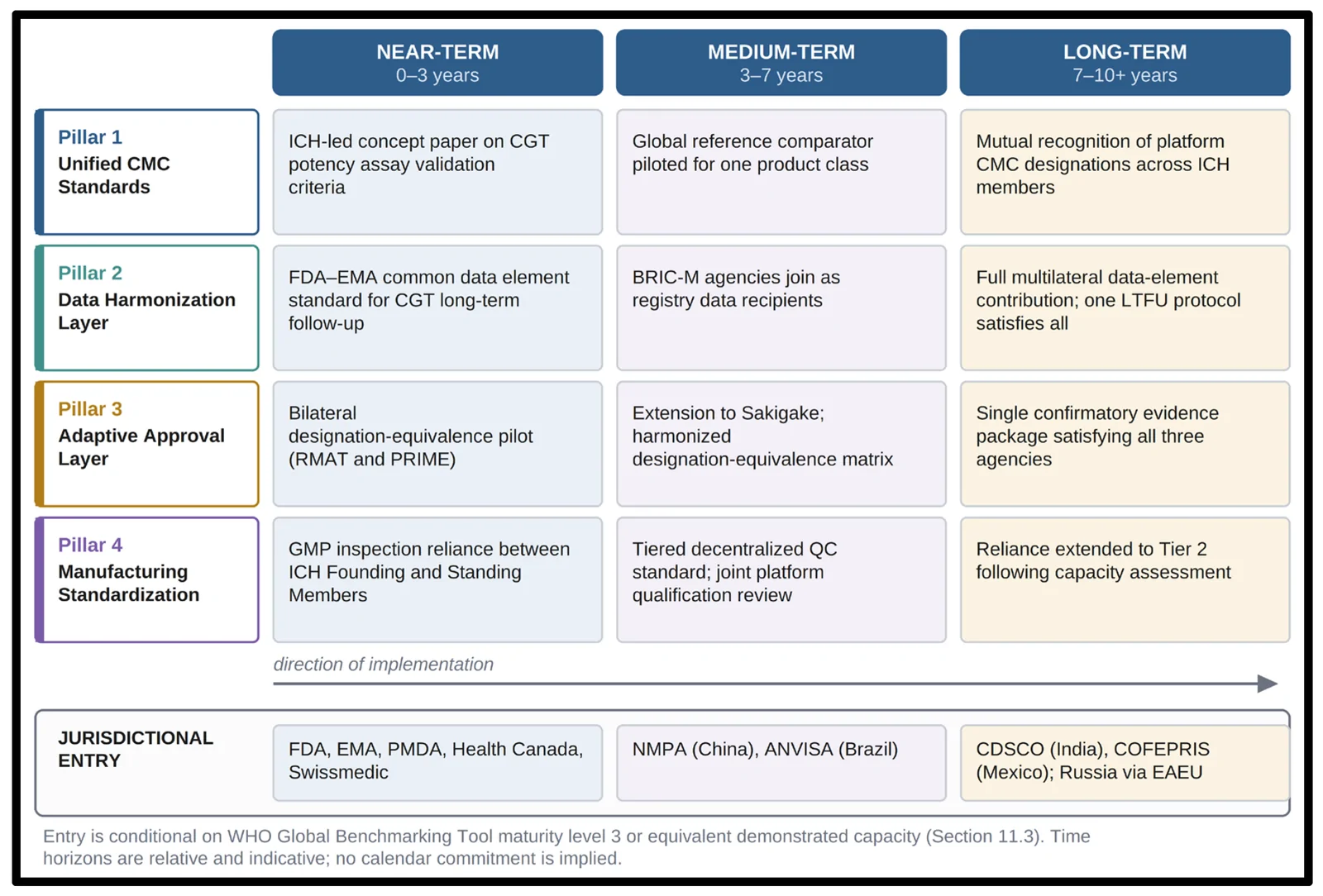
Phased implementation roadmap for the Global CGT Regulatory Convergence Framework, showing indicative milestones for each pillar across three-time horizons and the corresponding sequence of jurisdictional entry. The four pillars are designed to advance in parallel rather than sequentially, and each near-term milestone is achievable within existing ICH and ICMRA governance without new legal instruments. Jurisdictional entry is tiered by demonstrated regulatory-science maturity and is conditional on capacity thresholds rather than scheduled, consistent with the WHO Global Benchmarking Tool framework discussed in Section 11.3. Time horizons are relative and indicative; no calendar commitment is implied. CMC, chemistry, manufacturing, and controls; EAEU, Eurasian Economic Union; GMP, good manufacturing practice; LTFU, long-term follow-up; QC, quality control; RMAT, Regenerative Medicine Advanced Therapy.

For viral vector production specifically, the manufacturing standardization layer endorses accelerated regulatory qualification pathways for validated platform manufacturing innovations such as the SCL system for lentiviral vector production demonstrated by Arrasate et al. [146], recognizing that manufacturing platform innovations reducing batch-to-batch variability serve the harmonization agenda directly by reducing the raw material and process variability burden documented in Section 4.1 and Section 5.3. A jointly reviewed platform qualification package, submitted once to a lead agency and made available for expedited secondary review by other participating agencies, would reduce the current requirement for independent platform requalification in each jurisdiction where a developer seeks marketing authorization (Figure 5).

### 8.6. Legal and Institutional Constraints on Implementation

The mechanisms proposed in Section 8.2, Section 8.3, Section 8.4 and Section 8.5 extend arrangements that already operate between regulators, but each faces constraints that must be stated plainly if the framework is to be treated as anything other than aspirational. The most significant constraint concerns manufacturing oversight. Mutual reliance on good manufacturing practice inspections is established between the European Union and the United States under the Mutual Recognition Agreement, whose operational phase began on 1 November 2017 and was extended to veterinary products in 2023 [193,194]. However, the Agreement expressly excludes advanced-therapy medicinal products from its scope, alongside human blood, plasma, tissues and cells [194]. ATMP-specific GMP mutual recognition consequently does not exist between any pair of regulators at present. The 56 participating authorities of the Pharmaceutical Inspection Co-operation Scheme share inspection reports and harmonized GMP standards, but PIC/S is a voluntary association without treaty status and does not compel recognition of GMP certificates [84]. Pillar 4 therefore proposes an extension of an existing model into a product class from which that model has so far been excluded, and the exclusion is a deliberate regulatory judgement reflecting the process-dependent nature of ATMP manufacture rather than an oversight.

A second constraint concerns sovereignty over benefit–risk determination. Every work-sharing arrangement currently in operation preserves independent national decision-making. Under the Access Consortium New Active Substance Work-Sharing Initiative, participating agencies share assessment work, but each issues its own decision [171]. The WHO Good Reliance Practices framework states this explicitly: the relying authority “remains independent, responsible and accountable for the decisions taken” [195]. Nothing in the framework proposed here alters that position, and the acceptance of a common evidence package should be understood as convergence in what is submitted rather than in what is decided.

A third constraint concerns data. Shared long-term follow-up datasets would engage data protection law that differs materially across the jurisdictions examined, including the General Data Protection Regulation in the European Union and distinct regimes in China, Russia and Brazil. The interoperable registry architecture proposed in Pillar 2 assumes federated analysis of data held nationally rather than transfer of individual-level records, and this distinction is essential to its feasibility.

Finally, regulatory convergence should not be equated with improved patient access. Analysis of products reviewed through Project Orbis found that faster regulatory approval did not translate into faster reimbursement or availability across participating countries [196]. Pricing, health technology assessment, and health system capacity operate independently of regulatory alignment, and the framework proposed here addresses only the regulatory component of a wider access problem.

## 9. Emerging Technologies Outpacing Regulatory Science: Artificial Intelligence, Digital Twins, Genome Editing, and Non-Viral Delivery

The regulatory frameworks examined throughout this review were largely developed to address the CMC, clinical, and manufacturing characteristics of first- and second-generation cell- and gene-therapy (CGT) products, CAR-T-cell therapies, AAV-based in vivo gene replacement, and ex vivo lentiviral gene modification. A distinct set of emerging technologies is now advancing more rapidly than the regulatory science required to evaluate them: artificial intelligence (AI) applied to regulatory decision-making itself, digital twin technology for manufacturing process control, precision gene-editing modalities extending beyond first-generation CRISPR-Cas9 nucleases, and mRNA/non-viral delivery platforms that increasingly compete with viral vectors for both ex vivo and in vivo CGT applications. This section examines the regulatory implications of each technology in turn, with particular attention to where existing frameworks described in Section 4, Section 5, Section 6 and Section 7 are being extended, strained, or rendered structurally inapplicable.

### 9.1. Computational Methods in CGT Development: Regulatory Science Gaps

Artificial intelligence and digital twin modeling are increasingly applied in CGT development, and both create regulatory-science gaps relevant to the convergence agenda. Their relevance here is narrow and specific: neither is yet accommodated within CGT quality assessment frameworks in any of the jurisdictions examined, and each raises questions about evidentiary standards that will require harmonized answers if divergence is not to be recreated in a new domain. For CGT specifically, AI applications concentrate on bioprocess optimization and analytics rather than on submission content, which places them within the manufacturing-control space that Pillar 1 addresses. Digital twin approaches to process modeling raise the question of what constitutes adequate qualification of an in silico model used to support a release decision, a question no agency has yet answered for advanced therapies. We note these as prospective convergence requirements rather than examining them in depth, since neither is yet the subject of binding regulatory expectation in any jurisdiction studied.

### 9.2. Genome Editing: Off-Target Assessment and Regulatory Uncertainty

The regulatory science addressing CRISPR-based and related precision gene-editing modalities remains substantially less mature than the frameworks governing viral vector-based gene addition therapies discussed in Section 4 and Section 5, notwithstanding the landmark 2023–2024 approval of exagamglogene autotemcel (Casgevy), the first CRISPR-Cas9-based therapy authorized by the FDA, the EMA, and the UK’s MHRA for severe sickle-cell disease and transfusion-dependent β-thalassemia [197]. The multi-stakeholder GenE-HumDi network, established under the European Cooperation in Science and Technology (COST) Action framework and reported by Cavazza et al. [198], directly addresses this maturity gap: convening pharmaceutical companies, academic institutions, regulatory agencies, and patient advocacy groups across working groups focused on technology enhancement, delivery system assessment, safety characterization, clinical translation, and explicitly regulatory guideline development, with a stated objective of establishing standard procedures to harmonize scientific practice ahead of, rather than in reaction to, an expanding gene-editing clinical pipeline. The existence of a dedicated pan-European regulatory harmonization working group for gene editing, distinct from the broader ATMP and CGT regulatory apparatus discussed in Section 7, itself signals that existing ICH and EMA/CAT frameworks are viewed by the field as insufficiently tailored to gene-editing-specific regulatory science needs.

Off-target editing characterization remains the most consequential unresolved regulatory science gap for CRISPR-based products. As discussed in Section 4.5 with respect to genotoxicity monitoring more broadly, the acceptance criteria for novel approach methodologies used to detect off-target editing events, including unbiased genome-wide detection platforms, have not been harmonized across the FDA, EMA, and PMDA. Lopes and Prasad [199] provide a comprehensive evaluation of off-target risk mitigation strategies across the CRISPR development pipeline, concluding that while substantial technical progress has been made in both computational prediction and experimental detection of off-target events, no single methodology has achieved the status of a universally accepted regulatory standard, a conclusion consistent with the ICH S12 [8] biodistribution and integration framework’s continued gaps with respect to gene-editing-specific safety endpoints, as noted in Section 4.5.

Regulatory uncertainty is most acute for gene-editing modalities that extend beyond first-generation somatic nuclease editing. Shchukina et al. [200] provide a comprehensive technical review spanning CRISPR nuclease editing, base editing, prime editing, and emerging DNA polymerase-based editors, concluding that while foundational technological capability has been established across these modalities, clinical translation requires systematic resolution of manufacturing scalability, long-term safety assessment, and delivery challenges alongside development of regulatory frameworks specifically structured to accommodate rapid technological innovation, a call directly analogous to the anticipatory regulatory science argument developed in Section 7.4 of this review. At the furthest boundary of regulatory uncertainty, Shah et al. [201] examine CRISPR/Cas9 applications in human germline and reproductive contexts, concluding that heritable genome editing remains fundamentally a research tool rather than a viable near-term clinical modality, constrained by unresolved off-target mutagenesis, embryo mosaicism, and, critically for this review’s global convergence focus, substantial international governance disparities regarding the permissibility of heritable genome modification, a divergence in kind rather than merely in degree from the CMC and evidentiary harmonization gaps addressed elsewhere in this review, since it reflects differing national ethical and legal positions rather than differing technical regulatory science maturity.

### 9.3. mRNA and Lipid Nanoparticle Delivery: CMC and Regulatory Considerations

The clinical and manufacturing success of mRNA–lipid nanoparticle (LNP) platforms during the COVID-19 vaccine effort has directly accelerated the application of mRNA and non-viral delivery technologies to CGT product design [202]. Offering a potential resolution to several of the viral vector manufacturing bottlenecks documented in Section 5.2, including production capacity constraints, batch-to-batch variability, and the integration-related genotoxicity risks discussed in Section 4.5, Wang et al. [203], reviewing non-viral and mRNA-based strategies for next-generation CAR-T-cell therapy, describe the integration of mRNA engineering with lipid nanoparticle, electroporation, and exosome-based delivery platforms as a route toward both safer ex vivo CAR-T manufacturing and, more consequentially, in vivo CAR-T generation directly within the patient, eliminating reliance on patient-derived autologous starting material and its associated batch-variability challenges discussed throughout Section 4.1.

De la Mota et al. [204], in a comparative analysis of viral and non-viral vector platforms for in vivo CAR-T therapy, directly address the regulatory implications of this shift: non-viral platforms, including LNP and polyplex-based delivery, offer advantages in packaging capacity, redosing potential, and manufacturing scalability validated at the population scale by mRNA-LNP COVID-19 vaccine production, a manufacturing precedent with no equivalent in the viral vector space, as discussed in Section 5.2, while introducing distinct regulatory evaluation questions, including transient-versus-stable transgene expression duration, in vivo biodistribution and off-target cell transduction characterization, and, for DNA-based LNP platforms employing transposon systems for stable CAR transgene integration, genotoxicity assessment considerations that partially converge with, but are not identical to, those developed for viral-integrating vectors, as discussed in Section 4.5.

A specific regulatory classification challenge arises from the diversity of non-viral delivery mechanisms now under clinical investigation. Rallis et al. [205], reviewing the expansion beyond autologous CAR-expressing cell therapies toward allogeneic and nucleated cell-free CAR delivery systems, describe targeted lipid nanoparticles, synthetic DNA nanocarriers, and hybrid allogeneic-cell-as-carrier platforms, each of which presents a distinct combination of gene therapy, cell therapy, and, in some configurations, combination product regulatory characteristics that do not map cleanly onto the product classification frameworks established under the EU ATMP Regulation [32] or the FDA’s CBER product jurisdiction determinations. Existing EMA classification guidance for ATMPs [206] and FDA gene-therapy CMC guidance [207] were both developed with viral vector-based and ex vivo cell-based products as the primary reference case; their applicability to transient, non-integrating, cell-free mRNA-LNP products administered for in vivo cell engineering, which may not meet the traditional ATMP or biologic product definitions in every jurisdiction, remains an open regulatory science and classification question that this review identifies as a priority area requiring dedicated agency guidance, extending beyond the CMC and evidentiary harmonization gaps addressed in Section 4, Section 5, Section 6 and Section 7.

## 10. Ethical, Equity, and Access Considerations

Regulatory convergence is necessary but not sufficient for achieving equitable global access to CGT. Even where CMC, evidentiary, and pathway harmonization is achieved, extraordinary acquisition cost combined with required delivery infrastructure creates a distinct and largely independent access barrier that regulatory harmonization alone cannot resolve, and one that stands in tension with the accelerated-approval mechanisms examined in Section 2 and Section 7.3, which shorten time to authorization without addressing affordability at the point of delivery.

### 10.1. Pricing Disparities and Affordability

Approved CGT products carry unprecedented list prices: exagamglogene autotemcel (Casgevy), the first CRISPR-based therapy, was priced at $2.2 million per US patient. An ethics analysis of this pricing decision argues that the extremely high cost of Casgevy and comparable products constitutes a major global-health-equity concern, compelling an obligation of justice to maximize affordable access for patients with serious genetic disease grounded in the same unmet-medical-need logic underlying RMAT/PRIME/Sakigake (Section 2) while acknowledging manufacturing complexity, small populations, and development risk as legitimate but insufficient justification for pricing that structurally excludes the majority of the global patient population [208]. This position finds institutional expression in the WHO Guideline on Country Pharmaceutical Pricing Policies, establishing affordable, equitable access as the explicit objective Member States should pursue through price transparency, value-based pricing, and coordinated international mechanisms [209].

Novel payment models have emerged, recognizing that conventional volume-based pricing/reimbursement is poorly suited to one-time, potentially curative interventions. Analysis of innovative payment models for sickle-cell gene therapies within US Medicaid examines CMMI’s Cell and Gene Therapy Access Model, employing outcome-based, multi-year payments tied to real-world clinical response rather than upfront lump-sum reimbursement [210,211]. A systematic review of personalized-medicine financing/reimbursement identifies installment payments, outcome-based managed-entry agreements, and annuity-style reimbursement as dominant emerging categories, concentrated in a small number of high-income systems with the administrative/data infrastructure to operationalize multi-year outcome tracking, an infrastructure requirement directly limiting exportability to BRIC-M health systems (Section 10.3) [212].

China illustrates an alternative, domestically driven affordability pathway: domestically manufactured CAR-T products have achieved clinical outcomes comparable to global benchmarks (79–89% overall response rates in B-cell malignancies), while a multi-tiered “1+3+N” payment system combining national basic insurance, supplementary commercial insurance, and regional pilot programs has reduced patient out-of-pocket costs by approximately 50% [213]. Complementing this, Fosun Kite Biotechnology’s 2024 outcome-based payment plan for its domestically manufactured, China-licensed CD19-directed CAR-T product refunds patients up to approximately US$84,000 if complete remission is not achieved within three months, financed through city-level supplementary commercial insurance now underwritten by multiple insurers across more than 75 commercial products offering CAR-T coverage nationally [214], a domestic manufacturing/public risk-sharing/manufacturer-led pricing combination structurally distinct from US/EU models, reflecting China’s expanding domestic development base (Section 3.1).

### 10.2. Rare-Disease Equity in BRIC-M Populations

Resolution WHA78.11, adopted by the Seventy-eighth World Health Assembly on 24 May 2025, records that more than 7000 known rare diseases affect over 300 million people globally, that approximately 70% of these conditions have pediatric onset, and that the majority remain without effective treatment. Critically for the present analysis, the resolution identifies cost as a determinant of delayed and inequitable access even where treatments exist, particularly in low- and middle-income countries, and calls for strengthened international cooperation to secure equitable and affordable access to rare-disease care [215]. The translational pathway from CGT discovery to patient access is structurally biased toward high-income countries, compounding rather than merely paralleling Section 2.4’s regulatory divergence. An analysis of the LMIC gene-therapy translational gap documents that the overwhelming majority of clinical development, approval, and access remains concentrated in a small number of high-income jurisdictions, identifying insufficient local regulatory/manufacturing capacity, limited trial infrastructure, and absent adapted reimbursement as compounding barriers [216], directly relevant to BRIC-M, since China possesses commercial-scale CGT biomanufacturing capacity and India an expanding, if still pre-commercial, domestic base [53,88,89] (Section 3.1.3, Section 3.4.3 and Section 5), while Russia, Brazil and Mexico face more acute translational gaps [75,83,97], indicating that BRIC-M is analytically useful but not homogeneous in actual access capacity. This heterogeneity has direct equity implications for rare-disease populations specifically, since rare/ultra-rare genetic diseases, the predominant CGT target indications (Section 6.1), affect small absolute numbers per national population, a constraint compounded where the health system lacks specialized rare-disease diagnostic/referral infrastructure. An analysis of global access to multiple myeloma therapies, including CAR-T, documents substantial geographic disparities attributable not only to cost and approval status but also to specialized diagnostic, apheresis, and post-infusion monitoring infrastructure, which is concentrated in a limited number of accredited centers even within high-income systems (Section 5.1), let alone across BRIC-M populations, which have fewer specialized centers per capita [217].

### 10.3. Access Barriers in BRIC-M Healthcare Systems

Beyond approval status and drug cost, BRIC-M health systems face structural CGT-delivery barriers not captured by regulatory-harmonization analysis alone. An examination of CAR-T access across Latin America identifies a multi-layered barrier structure: regulatory approval delays relative to the FDA/EMA (documented for ANVISA in Section 3.2 and Section 7.4), absent national reimbursement pathways for cell therapies in most systems, insufficient accredited apheresis/administration centers, and shortages of personnel trained in CAR-T-specific toxicity management (cytokine release syndrome, ICANS), highlighting the need for regional regulatory cooperation analogous to Section 7.2’s reliance mechanisms and phased infrastructure investment prioritizing regional centers of excellence over simultaneous national-scale capacity building [218].

Mexico’s experience illustrates targeted clinical-program development improving access absent comprehensive CGT infrastructure: a modified adolescent/young-adult ALL treatment protocol, tailoring internationally validated protocols to local capacity rather than direct high-income-country importation, achieved meaningful outcome improvements despite distant full infrastructure parity [219], consistent with phased, context-adapted implementation strategies [218], suggesting that BRIC-M access strategies succeed more with incremental adaptation than aspirational full-parity timelines.

The EU hospital exemption pathway (Section 5.1), enabling institutional access outside centralized authorization [32], offers an instructive structural parallel: just as HE implementation varies substantially across Member States, BRIC-M health systems show comparable within-group heterogeneity (Brazil’s SUS incorporates advanced-therapy HTA through CONITEC [220], while comparable formal pathways remain less developed elsewhere), reinforcing that BRIC-M is a useful comparative category but not a homogeneous bloc, directly relevant to the phased Data Harmonization Layer participation model proposed for BRIC-M under the GCRC-F.

## 11. Future Directions: Institutional, Geographic, and Technological Determinants of Convergence

The regulatory convergence gaps documented throughout this review, and the GCRC-F proposed in response, raise a forward-looking question: what institutional, technical, and geographic developments will most determine whether meaningful CGT convergence is achieved over the coming decade? This section considers four developments: unified global agency feasibility, mutual-recognition expansion to BRIC-M, capacity-building as a convergence precondition, and AI integration into regulatory review itself, each assessed against the evidence established in Section 2, Section 3, Section 4, Section 5, Section 6, Section 7, Section 8, Section 9 and Section 10.

### 11.1. Is a Global CGT Regulatory Agency Feasible?

A single, unified global CGT regulatory authority merits explicit consideration primarily to establish why this review, consistent with its GCRC-F proposal, does not recommend it in the near term. No existing international health-governance instrument confers binding marketing-authorization authority across sovereign jurisdictions, and CGT regulation in every jurisdiction examined—the FD&C Act, the EU centralized authorization regulation, Japan’s PMD Act, and BRIC-M national drug laws—remains constitutionally domestic. A supranational agency would require a treaty-level agreement with no pharmaceutical-regulation precedent and no realistic near-term political feasibility.

A more tractable, already partially operational alternative is the WHO-Listed Authority (WLA) framework: a qualitative study describes how the WHO Global Benchmarking Tool provides an evidence-based methodology designating national authorities meeting defined performance standards as WLAs, promoting reliance among NRAs, the WHO prequalification program, and procurement agencies without requiring sovereign-authority cession, with substantial progress documented among transitional authorities, including several former Stringent Regulatory Authorities, though resource constraints and multi-stakeholder consensus-building complexity remain limiting [221,222]. This reliance-based architecture aligns with the GCRC-F’s modular design and echoes the biosimilar-harmonization precedent (Section 8.1): technically specific instruments have shown more durable uptake than unification proposals [185]. ICMRA already coordinating regulatory science priorities across ICH and non-ICH agencies (Section 7.2) represents the most plausible vehicle for extending WLA-style reliance principles to CGT-specific technical domains (potency standards, platform CMC recognition, LTFU data standards) rather than through new supranational infrastructure [173].

### 11.2. Expansion of Mutual Recognition to BRIC-M

Section 7.2’s mutual recognition/work-sharing mechanisms, principally the Access Consortium’s New Active Substance Work-Sharing Initiative (NASWSI) [171,192], currently operate among agencies sharing comparable regulatory-science maturity and domestic manufacturing-oversight capacity. Extending equivalent architecture to BRIC-M confronts a structural precondition this review’s evidence indicates is not yet uniformly satisfied: reliability of the underlying manufacturing/quality-assurance base that any mutual-recognition arrangement must rest upon. A qualitative study of tiered pharmaceutical manufacturing, based on 31 semi-structured interviews with predominantly India-based industry/procurement/regulatory experts, documents that manufacturers commonly apply differentiated production standards by destination market: products for WHO-Listed (formerly “Stringent”) Regulatory Authority jurisdictions are held to more rigorous documentation, stability, and impurity standards than products for other markets, framing this as a commercial determinant of health with direct implications for medicine quality/equity and identifying strengthened reliance, WHO prequalification, and procurement conditionality as the primary counteracting levers [223]. This bears directly on the extension of mutual recognition to BRIC-M for CGTs: reliance presupposes a verifiable, harmonized manufacturing standard that tiered practices, where present, would undermine. Within BRIC-M, substantial heterogeneity in mutual-recognition readiness exists (Section 3.6 and Section 7.2): China’s NMPA and Brazil’s ANVISA, both ICH participants [73], are more tractable candidates than Russia, India, or Mexico. A phased extension beginning with the most ICH-aligned agencies, structured around the WLA pathway [221] rather than uniform simultaneous expansion, is consistent with both the tiered-manufacturing evidence and the phased participation model summarized in Figure 5 (Section 8.3).

### 11.3. BRIC-M Capacity Building as a Convergence Prerequisite

The World Health Assembly formally recognized regulatory capacity as a global health priority in Resolution WHA67.20, urging sustained national investment as a precondition for reliable, quality-assured medical-product access directly underpinning the WHO Global Benchmarking Tool and WLA framework (Section 11.1) [224]. Neither a unified global agency nor expanded mutual recognition can substitute for underlying capacity where not yet established: a WHO global-competency-framework pilot across three implementation scenarios reports that fewer than 30% of countries globally have achieved WHO regulatory maturity level three, the generally understood prerequisite for reliable independent decision-making, with low- and middle-income countries facing particular difficulty attracting/retaining qualified personnel [225]; the competency-based approach explicitly supports not only national performance but also regional harmonization, shared assessment, and joint GMP inspection directly relevant to Section 11.2’s expansion.

Two case studies illustrate both feasibility and extended timelines: over four decades (1978–2020), Tanzania progressed from limited effectiveness to regional East African harmonization leadership through sustained multi-decade investment across legal-framework evolution, stakeholder engagement, capacity building, and organizational leadership, supported by the Global Fund, WHO, and UNDP [226]; conversely, Pakistan’s substantial domestic manufacturing industry has not translated into regulatory maturity, with a near-total absence of published medicine-quality data leaving the true scope of its substandard/falsified medicine problem uncharacterized [227], reinforcing that manufacturing scale alone does not equate to regulatory maturity, and that capacity-building requirements are not uniform across BRIC-M (Section 3.6 and Section 10.2). A 2024 pharmacovigilance-maturity survey across Developing Countries’ Vaccine Manufacturers Network members, including Brazil-based manufacturers, found markedly heterogeneous readiness, with some meeting WHO Global Benchmarking Tool/ICH standards with AI-supported case management, while others were constrained across case management, signal management, and risk management domains [228]—concrete, product-class-adjacent evidence (directly relevant to CGT post-approval surveillance, Section 4.4 and Section 6.3) that BRIC-M capacity building cannot be assumed to progress uniformly even within a single jurisdiction’s manufacturing base, reinforcing this review’s recommendation for phased, differentiated GCRC-F Data Harmonization Layer integration.

Two evidence gaps limit what can currently be claimed about the consequences of regulatory divergence. Differential approval timing between jurisdictions is quantified systematically for medicines as a whole [229], but we identified no published quantification of the cost or time impact of divergent CMC and comparability requirements specific to cell and gene therapy, and no robust measure of CGT access inequity between high- and middle-income settings. The associations described in Section 4 and Section 10 are therefore reasoned inferences from the general medicine literature rather than quantified CGT-specific relationships. Establishing these quantitatively, ideally through sponsor-reported data on duplicative requirements across jurisdictions, would materially strengthen the case for convergence and would be a valuable contribution to the field.

### 11.4. Integration of AI-Driven Regulatory Review Systems

Section 9.1, on AI-adoption implications, extends directly into the regulatory review process itself. The narrative review identifying ML, NLP, and RPA as regulatory-workflow tools specifically notes AI-assisted dossier review and signal detection’s potential to compress timelines that have historically diverged across agencies, which is directly relevant to the FDA-EMA timeline divergence documented in Section 7.4 [230]. Pharmacovigilance offers the most operationally mature illustration: Biogen’s internal comparison of a proprietary machine-learning regression decision-tree model against five industry-standard disproportionality-based signal-detection algorithms across seven marketed biologics found superior ML performance in time-to-detection and case capture, though derived from a single company’s internal database without external validation [231], which is directly relevant to Section 4.4 on the LTFU/surveillance harmonization gap and Section 6.3 and Section 8.3 on RWE data-quality infrastructure, since validated, cross-agency-qualified AI signal-detection tools could materially strengthen Pillar 2 shared post-approval evidence infrastructure.

The trajectory toward harmonized AI qualification is already visible bilaterally, extending Section 7.3’s FDA-EMA PSA coordination into AI-specific regulatory science: FDA’s January 2025 AI draft guidance [232] establishes a risk-based credibility framework, with a parallel FDA-EMA joint guiding-principles initiative reported to be under development. For CGT products specifically, the International Society for Cell and Gene Therapy (ISCT) guidance [233] identifies the absence of standardized terminology and validated qualification methodologies as primary barriers within CGT manufacturing and, by extension, any future AI-assisted CGT dossier review, a domain where Section 4’s biological complexity and manufacturing heterogeneity pose materially greater AI-qualification challenges than for conventional products. Whether AI-driven review accelerates or further complicates convergence depends substantially on whether emerging bilateral FDA-EMA credibility standards are extended, through the ICH or ICMRA, to PMDA and BRIC-M agencies before divergent national AI regulatory-science standards become entrenched as a genuine, time-sensitive harmonization priority.

The framework proposed in Section 8 has not been evaluated empirically, and its adoption would require validation before implementation could reasonably be considered. Three methods would be appropriate. First, a modified Delphi consensus process involving regulators from the participating tiers, manufacturers, patient organizations, and clinical developers would test whether the four pillars are perceived as workable and correctly prioritized. Second, retrospective case-study application to products that have already undergone multi-jurisdictional review, such as those assessed under Project Orbis or the Access Consortium, would indicate whether the framework would have altered timelines or evidentiary requirements. Third, a legal feasibility assessment conducted jurisdiction by jurisdiction would establish which elements could be implemented under existing statutory authority and which would require legislative change. Until such work is undertaken, the implementation sequence set out in Section 8 should be read as a structured hypothesis rather than a validated roadmap.

### 11.5. Limitations

This review has limitations that qualify its conclusions. Source selection was restricted to material available in English or in an official English translation. Regulatory instruments in Portuguese, Russian, Mandarin, and Spanish were accessed through official translations where available; where they were not, secondary analyses were used. Nuances of legal drafting may not survive translation, and this limitation applies most acutely to the Russian and Chinese frameworks. The regulatory environment described here is changing rapidly. Positions are stated as of 30 June 2026. Several instruments discussed were in draft form on that date. Any comparative regulatory analysis is a snapshot, and readers should verify current status before relying on specific statements.

The public availability of official documents is uneven. The FDA, EMA and PMDA publish assessment reports, guidance and inspection outcomes systematically; several of the other authorities examined do not. Where the public record is thinner, our characterization rests on fewer sources, and the depth of analysis is correspondingly unequal across jurisdictions. For the same reason, data exclusivity and enforcement mechanisms could not be compared systematically and are omitted from the comparative tables. The systems compared are heterogeneous in ways that resist direct comparison. They differ in legal tradition, in the division of authority between national and regional bodies, and in whether CGT products are regulated under dedicated instruments or general pharmaceutical law. Comparative tables necessarily simplify these differences.

This is an interpretive synthesis conducted by two authors. Source selection, the maturity descriptors in Table 2, and the convergence-readiness scoring all involve judgement. The rubric in Supplementary Table S1 is provided so that these judgements can be inspected and contested, but no independent adjudication, multi-rater scoring or sensitivity analysis was performed, and the classification should be read as an illustrative heuristic rather than a validated index. We further note that no regulatory authority holds a CGT-specific maturity designation, as the WHO Global Benchmarking Tool assessment covers medicines and vaccines streams only.

The proposed framework has not been evaluated by regulators, manufacturers, patient organizations, or clinical developers. Its feasibility is untested, and the implementation sequence in Section 8 should be read as a structured hypothesis rather than a validated roadmap. Finally, we identified no published quantification of the cost or time impact of divergent CGT-specific CMC and comparability requirements, and no robust measure of CGT access inequity between high- and middle-income settings. Where this review describes associations between regulatory divergence and delayed or unequal access, these are reasoned inferences from the general medicines literature rather than quantified CGT-specific relationships.

## 12. Conclusions

Cell and gene therapies have moved decisively from experimental proof of concept to established, and in several disease areas curative, clinical practice. Yet the regulatory architecture governing their development, manufacture, and lifecycle management remains organized around jurisdictional boundaries that predate this transformation. This review has traced that misalignment across CMC/manufacturing standards (Section 4 and Section 5), clinical evidence requirements (Section 6), the mechanics and limits of existing harmonization instruments (Section 7), the ethical/access consequences of unresolved divergence for BRIC-M populations (Section 10), and emerging technologies poised to further strain incomplete frameworks (Section 9). The consistent finding is not that harmonization has failed, but that it has been achieved unevenly, procedurally rather than substantively, and disproportionately among the agencies already closest in regulatory-science maturity, leaving CMC comparability, potency bridging, LTFU standards, and platform manufacturing recognition as the areas where duplicative burden and, consequently, delayed patient access, remain most acute.

Treating this as a global regulatory-ecosystem problem, rather than a series of bilateral or agency-specific gaps, is reinforced by evidence that formal alignment mechanisms do not reliably produce concordant outcomes even among the most closely coordinating agencies: widening rather than narrowing FDA-EMA orphan drug discordance [178] (Section 7.4) is difficult to reconcile with an incremental, agency-by-agency approach, indicating that CMC, evidentiary, and manufacturing standards must be addressed as an interconnected system, the premise underlying the four-pillar GCRC-F (Section 8), rather than through isolated bilateral fixes. Extending this ecosystem view to BRIC-M is not a peripheral equity add-on: unresolved pricing, capacity, and manufacturing-reliability gaps [208] (Section 10 and Section 11) mean convergence achieved only among the FDA, EMA, and PMDA would leave the majority of the world’s CGT-eligible population outside its practical benefit.

The GCRC-F Unified CMC Standards, a Data Harmonization Layer, an Adaptive Approval Layer, and a Manufacturing Standardization Layer are this review’s central actionable response, deliberately modular rather than an omnibus instrument, reflecting the evidence (Section 7 and Section 11) that technically specific, incrementally adoptable proposals achieve more durable uptake than unification calls. Consistent with this, the framework builds on the WHO-Listed Authority reliance precedent [221] (Section 11.1) rather than proposing a supranational authority, achieving convergence through structured recognition and coordinated technical standards without requiring sovereign-authority cession. Each pillar corresponds to a specific, evidenced gap: potency bridging and platform CMC recognition (Section 4); LTFU/RWE standardization (Section 4.4 and Section 6.3); designation comparison across RMAT, PRIME, and Sakigake (Section 2 and Section 7.3); and GMP mutual reliance calibrated to BRIC-M manufacturing-reliability concerns (Section 5.4 and Section 11.2). Most notably, extracellular vesicles, exosome therapeutics, and conditioned secretome products currently confront parallel regulatory ambiguity, lack of class-specific pharmacopeial standards, and divergent regional oversight across North American, European, and Asian regulatory agencies. Extending the maturity-tiered reliance mechanisms, standardized potency-testing matrices, and multilateral data-sharing repositories established in the GCRC-F to the burgeoning sector of extracellular vesicle biologics constitutes a logical and imperative horizon for subsequent regulatory science research [234].

Implementation requires no new treaty-level authority or additional bureaucracy: its foundational components already exist in partial form—ICH S12 demonstrates that CGT-specific nonclinical harmonization is achievable within existing ICH governance [8], the Access Consortium’s work-sharing model demonstrates that multilateral review coordination functions operationally, and the FDA’s Platform Technology Designation demonstrates that agency-level manufacturing-platform recognition is already practiced in at least one major jurisdiction. The GCRC-F proposes deliberately interconnecting these proven components into a coordinated architecture, extended through the phased BRIC-M integration pathway (Section 8.3, Section 11.2 and Section 11.3) to jurisdictions not yet positioned for equal technical footing. Realizing it will require sustained ICH/ICMRA coordination, BRIC-M capacity investment as a precondition rather than an afterthought, and explicit attention to the pricing/access dimensions documented in Section 10, since regulatory harmonization alone cannot resolve the affordability barriers determining whether harmonized approval ultimately translates into patient access [208].

The transformative clinical potential of cell and gene therapies is no longer in question. Whether that potential is realized equitably and efficiently across the global patient population is principally a regulatory science and policy question, one that this review has argued is answerable through deliberate, modular, technically grounded convergence rather than incremental bilateral fixes or aspirational unification calls. The GCRC-F is offered as a concrete starting point: a roadmap whose value will ultimately be measured not in publication, but in the degree to which regulatory agencies, industry, and international coordinating bodies adopt, test, and refine its individual pillars in the years following this review.

## Figures and Tables

**Table 1:** Comparative regulatory features for cell- and gene-therapy products across the FDA, EMA, and PMDA. Regulatory positions are stated as of 30 June 2026 and were verified against the primary legislative instruments and agency guidance cited in each cell. BRIC-M comparables are given in Table 2.

Regulatory Feature	FDA (USA)	EMA (European Union)	PMDA (Japan)	Legal Status	Harmonized?
**Key legislation**	Public Health Service Act 351(42 U.S.C. §262); FD&C Act §506(g); 21st Century Cures Act (2016); 21 CFR Part 1271 [15,16]	Regulation (EC) No. 1394/2007 on ATMPs; Directive 2001/83/EC Regulation (EU) No. 536/2014 (clinical trials) [27,32]	PMD Act (2014); Act on Safety of Regenerative Medicine (ASRM, 2014) [29]	Statute (binding)	Partial convergence through ICH framework
**Expedited designation pathway**	RMAT designation (21st Century Cures Act §3033); Breakthrough Therapy; Accelerated Approval [16,20,33]	PRIME scheme; Conditional Marketing Authorization (CMA) [34,35]	Sakigake designation; Conditional and Time-Limited Approval (CTL) [20,30]	Administrative scheme (non-binding)	No formal cross-agency alignment; shared conceptual origin only. No product has held RMAT, PRIME and Sakigake concurrently [36].
**Approval philosophy**	Substantial evidence of effectiveness required; surrogate endpoints eligible for accelerated approval with confirmatory post-marketing commitment [16,20]	Positive benefit–risk balance with conditionality codified as an explicit approval state; confirmatory studies obligatory [27,28]	Time-limited early market access on limited efficacy evidence; mandatory post-marketing confirmation; full approval may be withheld (HeartSheet non-conversion, July 2024) [30]	Statute (binding)	Directionally convergent; diverges in pre-approval evidentiary threshold
**Long-term follow-up (LTFU) requirement**	Risk-stratified follow-up of up to 15 years for integration-competent vectors and genome-edited products; duration set per product—specifically through REMS and post-marketing commitments [37]	Product-specific Risk Management Plan (RMP) under the ATMP safety and efficacy follow-up guideline (EMEA/149995/2008); clinical follow-up of gene-therapy recipients addressed separately under EMEA/CHMP/GTWP/60436/2007; no universal time-defined framework [27,38,39,40]	7-year post-marketing confirmation period under CTL scheme; all-case surveillance applied to ~79% of approved products [30,41]	Federal regulation (binding)	Not harmonized; duration, mechanism, and enforcement differ across agencies
**GMP standard**	21 CFR Parts 210/211/600-680 and Part 1271; ICH Q7/Q10 adopted; CBER 2026 flexible CMC approach announced [15,26,42,43]	EudraLex Volume 4 including Annex 1 (sterile manufacture); ATMP-specific GMP guidelines (2017) issued under Regulation 1394/2007; ICH Q10 adopted [32,43,44]	J-GMP (ICH Q10-aligned); PMDA inspection framework; PMDA Notification No. 0709-2 (2019); post-marketing GMP surveillance [43,45]	National legislation (binding)	ICH Q7/Q10 provide shared foundation; product-specific standards diverge
**CMC comparability framework**	FDA 2023 draft guidance on manufacturing changes and comparability for CGT products; ICH Q5E adopted; platform CMC leveraging under the 2026 genome-editing draft guidance and the Platform Technology Designation Program [5,19,24,25]	ICH Q5E adopted (Step 5); risk-based provisions under Regulation 1394/2007; variation classification under Regulation (EC) No 1234/2008; EMA/CAT Q&A on ATMP comparability (EMA/CAT/499821/2019) [5,32,46,47]	ICH Q5E adopted; conditional approval scheme structured around post-marketing manufacturing data collection; additional site-specific validation obligations under the PMD Act [5,30,45]	Scientific guideline (non-binding)	ICH Q5E adopted by all three; directional alignment in lifecycle approach
**Real-world evidence (RWE) framework**	21st Century Cures Act framework; FDA 2018 RWE Framework and 2024 EHR/claims guidance; RMAT post-approval RWE pathway for confirmatory requirements [16,48]	Guideline on registry-based studies (EMA/426390/2021); RWE increasingly accepted for ATMP post-marketing obligations [49]	All-case surveillance following CTL approval; limited dedicated RWE guidance for regenerative medical products [30,41]	Final guidance (non-binding)	Not harmonized; registry design, endpoint acceptance, and data standards differ

Legal status classification: Statute or Regulation denotes binding primary legislation (for example, the FD&C Act, Regulation (EC) No. 1394/2007, or the PMD Act); CFR denotes binding US federal regulation; Final guidance denotes non-binding agency guidance representing current thinking, which under 21 CFR 10.115(d) does not establish legally enforceable responsibilities; Draft guidance denotes guidance issued for comment and not yet operative; Reflection paper denotes a non-binding scientific position. ICH guidelines are not directly binding and acquire effect only upon transposition by each regional authority. § indicates the section number.

**Table 5:** Existing international convergence initiatives relevant to cell and gene therapy, and the position of the proposed framework relative to them. The four pillars of the GCRC-F build on mechanisms already demonstrated by these initiatives rather than introducing novel regulatory instruments. Its distinguishing feature is the combination of a binding effect on participants, scope specific to cell and gene therapy beyond oncology, and structured inclusion of emerging-market regulators, a combination not offered by any existing mechanism.

Initiative	Scope	Participants	Binding?	CGT-Specific?	Emerging Markets?	Documented Limitation
ICH	Technical guidelines across quality, safety, efficacy	Members and Observers	Only on regional transposition	Partial (Q5A(R2), Q5E, S12)	Limited	Q5A(R2) excludes cell-based ATMPs; S12 omits integration and off-target testing; no common ATMP quality standard
CoGenT Global	Concurrent review of rare-disease gene therapies	FDA with ICH-region regulators	No	Yes	No	Announced January 2024; no documented completed collaborative review
Access Consortium NASWSI	Work-sharing for new active substances	Australia, Canada, Singapore, Switzerland, UK	No—sovereign decisions retained	No	No	25 approvals by June 2023; excludes US, EU, Japan and all emerging markets
Project Orbis	Concurrent oncology review	FDA plus 7 partners	No	No—oncology only	Partial (Brazil, Singapore)	Oncology-only by the FDA’s own statement; approval speed did not translate into faster reimbursement
APACRM	Regional regenerative medicine harmonization	Asian industry associations; regulators as panelists	No	Yes	Partial (Asia)	Industry dialogue forum; no work-sharing pathway or binding mechanism
WHO reliance/WLA	General regulatory reliance and authority listing	Global	No	No	Yes	General medicines and vaccines framework; no ATMP-specific assessment scope
GCRC-F (proposed)	CGT quality, CMC and approval convergence	Tiered, including pharmerging regulators	Proposed as binding on participants	Yes	Yes	Not yet validated by stakeholders (Section 11.5)

**Table 6:** Comparison of expedited designations and conditional approval pathways across the FDA, EMA and PMDA. The five mechanisms fall into two distinct categories that are not interchangeable: RMAT, PRIME and Sakigake are designations conferring development support and expedited interaction, whereas EU conditional marketing authorizations and Japan’s conditional and time-limited approval are approval pathways permitting marketing on less complete data. Differences in legal basis are material: RMAT and the two conditional approval pathways rest on statute, whereas PRIME operates as an administrative scheme without a dedicated legal instrument. Sakigake uniquely requires that the marketing application be filed in Japan first or simultaneously with other jurisdictions. Japan’s conditional and time-limited approval carries a real possibility of non-conversion: of the products approved under the scheme, HeartSheet was refused full approval in July 2024, and the application for beperminogene perplasmid was withdrawn during review, with the approval expiring [30,31].

Mechanism	Type	Legal Basis	Legal Status	Eligibility	Evidentiary Threshold	Key Benefit	Post-Authorization Obligation
RMAT (US)	Designation	FD&C Act §506(g), added by 21st Century Cures Act §3033 (2016)	Statute (binding)	Regenerative medicine therapy for a serious condition addressing unmet need	Preliminary clinical evidence	Breakthrough-equivalent interactions; early surrogate endpoint discussion; possible accelerated approval	Confirmatory studies if accelerated approval granted; registries or RWD may satisfy
PRIME (EU)	Designation	Administrative scheme, EMA/CHMP/57760/2015; launched 7 March 2016; no dedicated statute	Administrative scheme (non-binding)	Unmet medical need with potential major therapeutic advantage	Proof of concept; academic and SME applicants may enter on proof of principle	Early rapporteur appointment; iterative scientific advice; scientific coordinator	Noninherent—development support only
Sakigake (Japan)	Designation	Pilot April 2015; statutory under revised PMD Act from 1 September 2020	Statute (binding)	Innovative principle; serious disease; prominent expected efficacy; filing in Japan first or simultaneously	Non-clinical and early-phase clinical data	Prioritized consultation; pre-application review; target review ~6 months	Noninherent—development support only
Conditional MA (EU)	Approval pathway	Regulation (EC) No. 726/2004 Art. 14-a; Commission Regulation (EC) No. 507/2006	EU legislation (binding)	Positive benefit–risk; unmet need; comprehensive data likely later; benefit of immediate availability outweighs risk	Less complete data than a standard MA	Marketing authorization on incomplete data	Specific obligations under Art. 5; valid one year, renewable annually; convertible to standard MA
Conditional and time-limited approval (Japan)	Approval pathway	PMD Act, framework effective November 2014; MHLW guidance PSB/MDED Notification No. 0329-3 (March 2024)	Statute (binding)	Regenerative medical products only; probable benefit and proven safety	Probable benefit from exploratory trials; full-quality data still required	Time-limited marketing approval	Maximum 7 years; must reapply for full approval on post-marketing data or approval expires

Legal status classification: Statute or Regulation denotes binding primary legislation (for example the FD&C Act, Regulation (EC) No. 1394/2007, or the PMD Act); CFR denotes binding US federal regulation; Final guidance denotes non-binding agency guidance representing current thinking, which under 21 CFR 10.115(d) does not establish legally enforceable responsibilities; Draft guidance denotes guidance issued for comment and not yet operative; Reflection paper denotes a non-binding scientific position. ICH guidelines are not directly binding and acquire effect only upon transposition by each regional authority. § indicates the section number.

**Table 7:** The four pillars of the proposed Global CGT Regulatory Convergence Framework (GCRC-F), their target gaps, core mechanisms, and existing regulatory precedents. Each pillar is designed to be adoptable independently; the shared data standards described in Section 8.3 provide interoperability between pillars where agencies elect to adopt more than one.

Pillar	Target Gap (Section)	Core Mechanism	Existing Precedent Leveraged
1. Unified CMC Standards	Potency/comparability divergence (Section 4.2 and Section 4.3)	Harmonized potency validation criteria; global reference comparator; mutual platform-CMC recognition	FDA Platform Technology Designation Program [25]; Webster and Woollett global reference comparator model [125]
2. Data Harmonization Layer	LTFU/RWE fragmentation (Section 4.4 and Section 6.3)	Common data element (CDE) standard; phased BRIC-M registry participation	FDA-funded TRUST demonstration project [160]; CIBMTR and EBMT registries [49]; Strimvelis patient-centric registry model [126]
3. Adaptive Approval Layer	Designation fragmentation (Section 2 and Section 7.3)	Designation-comparison matrix across RMAT, PRIME and Sakigake	Eichler et al. adaptive pathways under the NEWDIGS initiative [190]; Kondo et al. comparative designation analysis [36]
4. Manufacturing Standardization Layer	GMP inconsistency (Section 5.4)	GMP mutual reliance; tiered decentralized QC standard	Access Consortium New Active Substance Work-Sharing Initiative [171,192]; Nahum et al. tiered QC framework [138]; Shin and Kim comparative GMP analysis [148]

## Data Availability

No new data were created or analyzed in this study.

## Supplementary Materials

The following supporting information can be downloaded at: https://www.mdpi.com/article/10.3390/biotech15040079/s1, Table S1: Convergence-readiness scoring rubric.

## Abbreviations

The following abbreviations are used in this manuscript:

AAV	Adeno-Associated Virus
AI	Artificial Intelligence
ANVISA	Agência Nacional de Vigilância Sanitária (Brazilian Health Regulatory Agency)
ATMP	Advanced-Therapy Medicinal Product
BCP	Biomedical Cell Product (Russian Federation)
BLA	Biologics License Application
BRIC-M	Brazil, Russia, India, China, and Mexico
BTD	Breakthrough Therapy Designation
CAR-T	Chimeric Antigen Receptor T Cell
CAT	Committee for Advanced Therapies (EMA)
CBER	Center for Biologics Evaluation and Research (FDA)
CDE	Center for Drug Evaluation (NMPA, China)
CDMO	Contract Development and Manufacturing Organization
CEP	Comitê de Ética em Pesquisa (Research Ethics Committee, Brazil)
CFR	Code of Federal Regulations (United States)
CGT	Cell And Gene Therapy
CHMP	Committee for Medicinal Products for Human Use (EMA)
CIBMTR	Center for International Blood and Marrow Transplant Research
CMC	Chemistry, Manufacturing, and Controls
COFEPRIS	Comisión Federal para la Protección contra Riesgos Sanitarios (Mexico)
CONEP	Comissão Nacional de Ética em Pesquisa (National Research Ethics Commission, Brazil)
CQA	Critical Quality Attribute
CRISPR	Clustered Regularly Interspaced Short Palindromic Repeats
CTA	Clinical Trial Application
CTD	Common Technical Document
CTL	Conditional and Time-Limited (approval, Japan)
CTLA-4	Cytotoxic T-Lymphocyte-Associated Protein 4
CTNBio	Comissão Técnica Nacional de Biossegurança (National Biosafety Technical Commission, Brazil)
DAL	Drug Administration Law (China)
DBT	Department of Biotechnology (India)
EAEU	Eurasian Economic Union
EBMT	European Society for Blood and Marrow Transplantation
EC	European Commission
EHR	Electronic Health Record
EMA	European Medicines Agency
EU	European Union
EWP	Efficacy Working Party (EMA)
FD&C Act	Federal Food, Drug, and Cosmetic Act (United States)
FDA	Food and Drug Administration (United States)
FIH	First-In-Human
FZ	Federalny Zakon (Federal Law, Russian Federation)
GCP	Good Clinical Practice
GCRC-F	Global CGT Regulatory Convergence Framework
GMCT	Genetically Modified Cell Therapy
GMO	Genetically Modified Organism
GMP	Good Manufacturing Practice
GTEAC	Gene Therapy Advisory and Evaluation Committee (India)
ICH	International Council for Harmonization
ICMR	Indian Council of Medical Research
ICMRA	International Coalition of Medicines Regulatory Authorities
IIT	Investigator-Initiated Trial
IN	Instrução Normativa
IND	Investigational New Drug
IPRP	International Pharmaceutical Regulators Program
ISCT	International Society for Cell and Gene Therapy
LNP	Lipid Nanoparticle
LTFU	Long-Term Follow-Up
MA	Marketing Authorization
MAH	Marketing Authorization Holder
MFDS	Ministry of Food and Drug Safety
MHLW	Ministry of Health, Labour and Welfare (Japan)
MHRA	Medicines and Healthcare products Regulatory Agency
ML	Machine Learning
MOA	Mechanism Of Action
MRCT	Multiregional Clinical Trial
NAM	Novel Approach Methodology
NASWSI	New Active Substance Work-Sharing Initiative
NDCT	New Drugs and Clinical Trials Rules (India)
NEWDIGS	New Drug Development ParadigmS
NHC	National Health Commission (China)
NIFDC	National Institutes for Food and Drug Control (China)
NMPA	National Medical Products Administration (China)
NOM	Norma Oficial Mexicana (Official Mexican Standard)
PANDRH	Pan American Network for Drug Regulatory Harmonization
PMD Act	Pharmaceuticals and Medical Devices Act (Japan)
PMDA	Pharmaceuticals and Medical Devices Agency (Japan)
PMS	Post-Marketing Surveillance
PRIME	PRIority MEdicines scheme (EMA)
PSA	Parallel Scientific Advice (FDA–EMA)
RCGM	Review Committee on Genetic Manipulation (India)
RDC	Resolução da Diretoria Colegiada (Collegiate Board Resolution, ANVISA, Brazil)
REMS	Risk Evaluation and Mitigation Strategy (United States)
RMAT	Regenerative Medicine Advanced Therapy
RMP	Risk Management Plan
RRA	Reference Regulatory Authority
RWE	Real-World Evidence
SCL	Stable Producer Cell Line
TGA	Therapeutic Goods Administration (Australia)
WHO	World Health Organization
WLA	WHO-Listed Authority
