# Supplementary material for: Regulatory Convergence in Cell and Gene Therapy: Harmonizing Quality, CMC, and Approval Pathways Across the FDA, EMA, PMDA, and Emerging Markets
Source: BioTech (Basel). 2026 Sep 16;15(4):79. doi: 10.3390/biotech15040079 (PMC13600107; doi:10.3390/biotech15040079)
Supplement: Supplementary file 1 [file biotech-15-00079-s001.zip › biotech-4514252-supplementary.pdf]

Supplementary Materials:

Regulatory Convergence in Cell and Gene Therapy: Harmonizing Quality, CMC, and Approval Pathways Across the FDA, EMA, PMDA, and Emerging Markets

Nagendra Verma and Swati Arora

Table S1. Convergence-readiness scoring rubric. Each jurisdiction is scored 0–2 against five indicators, all derived from published designations rather than authorial judgement.

Jurisdiction	ICH Status	WLA Status	PIC/S	Dedicated CGT Instrument	Domestic CGT Experience	Total /10
United States (FDA)	Founding Regulatory Member (1990) – 2	Designated WLA (May 2024) – 2	Participating (2011) – 2	PHS Act 351; 21 CFR 1271; RMAT – 2	Extensive – 2	10
European Union (EMA)	Founding Regulatory Member (1990) –2	EMRN-designated WLA (May 2024) – 2	Member States participating – 2	Regulation (EC) 1394/2007 – 2	Extensive – 2	10
Japan (PMDA)	Founding Regulatory Member (1990) – 2	Designated WLA (Aug 2025) – 2	Participating (2014) – 2	PMD Act 2014; ASRM – 2	Extensive – 2	10
China (NMPA)	Regulatory Member (2017) – 1	Transitional WLA – 1	Pre-accession applicant (2021) – 1	DAL 2019; CDE guidance series – 2	Domestic approvals; commercial GMP – 2	7
Brazil (ANVISA)	Regulatory Member (2016) – 1	Transitional WLA – 1	Participating (2021) – 2	RDC 505/506/2021; RDC 836/2023 – 2	Framework operational; limited scale – 1	7
Mexico (COFEPRIS)	Regulatory Member – 1	Transitional WLA – 1	Participating (2018) – 2	None dedicated – 0	None – 0	4
Russia (Minzdrav)	Observer – 0	Not listed – 0	Not participating – 0	Law 180-FZ (BCPs); Law 61-FZ – 2	Limited domestic activity – 1	3
India (CDSCO)	Observer – 0	Not listed – 0	Not participating – 0	NDCT Rules 2019; no dedicated category – 1	One domestic approval – 1	2
Republic of Korea (MFDS)*	Regulatory Member (2016) – 1	Designated WLA (Oct 2023) – 2	Participating (2014) – 2	ARMAB 2019/2020 – 2	Multiple approvals – 2	9
Singapore (HSA)*	Regulatory Member (2017) – 1	Designated WLA (Oct 2023) – 2	Participating (2000) – 2	CTGTP Regulations 2021 – 2	Framework operational – 1	8

ICH status: 2 = Founding or Standing Regulatory Member; 1 = Regulatory Member; 0 = Observer or non-participant. WLA status: 2 = designated WHO-Listed Authority; 1 = transitional WLA; 0 = not listed. PIC/S: 2 = participating authority; 1 = pre-accession applicant; 0 = not participating. Dedicated CGT instrument: 2 = dedicated instrument in force; 1 = general instrument applied to CGT, no dedicated category; 0 = none. Domestic CGT experience: 2 = multiple domestic approvals and commercial-scale GMP manufacturing; 1 = limited approvals or emerging capacity; 0 = none. Rows marked with an asterisk are reference jurisdictions outside the study frame (Section 3.7), included for comparison. The WHO Global Benchmarking Tool maturity level was deliberately excluded as a scored indicator. The FDA, EMA, and PMDA do not appear on the WHO-published ML3/ML4 list, having entered the WHO-Listed Authority framework through the legacy Stringent Regulatory Authority

route rather than through GBT benchmarking; scoring on maturity level would therefore misrepresent them. For context, published maturity designations among the jurisdictions studied are China ML3 (vaccines, producing, 2022) and India ML3 (vaccines, producing) only. No authority holds any CGT-specific maturity rating, as GBT benchmarking covers medicine and vaccine streams only. This rubric is an illustrative heuristic, not a validated index. No sensitivity analysis or independent multi-rater scoring was performed (Section 11.5)
